# Non-homologous isofunctional enzymes: A systematic analysis of alternative solutions in enzyme evolution

**DOI:** 10.1186/1745-6150-5-31

**Published:** 2010-04-30

**Authors:** Marina V Omelchenko, Michael Y Galperin, Yuri I Wolf, Eugene V Koonin

**Affiliations:** 1 National Center for Biotechnology Information, National Library of Medicine, National Institutes of Health, Bethesda, Maryland 20894, USA

## Abstract

**Background:**

Evolutionarily unrelated proteins that catalyze the same biochemical reactions are often referred to as analogous - as opposed to homologous - enzymes. The existence of numerous alternative, non-homologous enzyme isoforms presents an interesting evolutionary problem; it also complicates genome-based reconstruction of the metabolic pathways in a variety of organisms. In 1998, a systematic search for analogous enzymes resulted in the identification of 105 Enzyme Commission (EC) numbers that included two or more proteins without detectable sequence similarity to each other, including 34 EC nodes where proteins were known (or predicted) to have distinct structural folds, indicating independent evolutionary origins. In the past 12 years, many putative non-homologous isofunctional enzymes were identified in newly sequenced genomes. In addition, efforts in structural genomics resulted in a vastly improved structural coverage of proteomes, providing for definitive assessment of (non)homologous relationships between proteins.

**Results:**

We report the results of a comprehensive search for non-homologous isofunctional enzymes (NISE) that yielded 185 EC nodes with two or more experimentally characterized - or predicted - structurally unrelated proteins. Of these NISE sets, only 74 were from the original 1998 list. Structural assignments of the NISE show over-representation of proteins with the TIM barrel fold and the nucleotide-binding Rossmann fold. From the functional perspective, the set of NISE is enriched in hydrolases, particularly carbohydrate hydrolases, and in enzymes involved in defense against oxidative stress.

**Conclusions:**

These results indicate that at least some of the non-homologous isofunctional enzymes were recruited relatively recently from enzyme families that are active against related substrates and are sufficiently flexible to accommodate changes in substrate specificity.

**Reviewers:**

This article was reviewed by Andrei Osterman, Keith F. Tipton (nominated by Martijn Huynen) and Igor B. Zhulin. For the full reviews, go to the Reviewers' comments section.

## Background

The recent efforts in genome sequencing of organisms that inhabit a variety of environments, from deep-sea hydrothermal vents to Antarctic ice, revealed a surprising biochemical unity of these organisms, that is, the uniformity of the key gene expression mechanisms and metabolic pathways, and the enzymes that catalyze them. However, in certain cases, the same biochemical reaction is known to be catalyzed by two or more enzymes that share no detectable sequence similarity with each other [1,2]. Although this apparent lack of sequence similarity often can be attributed to the rapid divergence of homologous protein sequences during evolution [3], some of the alternative enzymes catalyzing the same biochemical reaction have been found to adopt different structural folds and therefore must have evolved independently. Enzymes that catalyze the same reaction are often referred to as analogous, as opposed to homologous [4-6]; it is probably more accurate to explicitly denote them Non-homologous ISofunctional Enzymes (NISE), and hereinafter we adopt this notation. One of the best-known cases of NISE is superoxide dismutase whose 3 principal forms, Cu/Zn-, Mn/Fe-, and Ni-dependent, are all structurally distinct [2,7,8] and there is evidence for the existence of yet another, fourth form [9].

In a previous study, in the early days of genome sequencing, we performed a systematic search for potential NISE by identifying all protein sequences listed in GenBank that, although assigned the same 4-digit Enzyme Commission (EC) numbers, had no detectable sequence similarity with each other [6]. Wherever possible, the independent origin of candidate NISE was validated by assigning them to distinct structural folds. As a result, apparently unrelated sequences were found for 105 EC nodes out of the 1709 nodes represented at that time in GenBank (see Additional file 1, Table S1). However, pairs of crystal structures confirming that two or more distinct forms of an enzyme indeed had different 3D folds were available only for 16 EC nodes. In 18 more cases, distinct folds for different enzyme isoforms were inferred on the basis of their sequence similarity to proteins with known 3D structures. For the rest of the EC nodes identified as potential NISE on the basis of the lack of detectable sequence similarity to each other, the structural relationships remained inaccessible, so there was no definitive proof of their evolutionary independence.

Our 1998 study suggested that NISE could be far more widespread than previously thought and paved the way to further recognition of candidate NISE catalyzing a variety of metabolic reactions [3,10,11]. However, subsequent structural studies showed that some of the pairs of enzymes, initially predicted to be NISE, actually included distantly related proteins. For example, the apparent lack of sequence similarity between bacterial and eukaryotic glutathione synthetases proved to be due to a circular permutation in the latter structure [12]. In addition, improved methods for protein sequence comparison made it possible to identify subtle sequence similarities between some of the candidate NISE that appeared to be indicative of their common origin.

The goal of the present study was to make use of the vastly expanded sequence and structural data that are currently available, to generate a comprehensive list of NISE and to obtain insights into the evolution of alternative solutions for the same reaction through comparison of the phyletic patterns of their distribution. In the years elapsed since our 1998 analysis, several studies explored various groups of alternative enzymes catalyzing the same biochemical reaction, including those belonging to the same protein (super)families [13,14]. Here, we focus on enzyme variants that possess (or could be inferred to possess) different structural folds and therefore appear to be evolutionarily unrelated (bona fide NISE).

## Results

### Update of the original list of non-homologous isofunctional enzymes

The previous analysis [6] identified 105 EC nodes (individual biochemical reactions) that included predicted analogous enzymes (NISE, under the present notation). Of these, previously characterized different folds were available for 16 EC nodes, thus validating 16 sets of NISE. For 13 of these 16, there were two isoforms with distinct structural folds. The remaining 3 enzymes, namely chloroperoxidase, cellulase and lichenase, were represented by 3 distinct folds each [6]. During the past 12 years, progress in structural genomics resulted in a rapid growth of the number of solved protein structures [15,16] including structures of many candidate NISE. As a result, comparison of many putative NISE pairs could be put on a solid structural footing. A re-analysis of the previously defined set of 105 EC nodes revealed 61 additional nodes of *bona fide *NISE that were represented by two or more versions with distinct folds (Additional file 1, Table S1). These sets of NISE included 17 cases with 3 distinct folds, 4 cases with 4 folds, and one instance where the same activity (protein-tyrosine-phosphatase, EC 3.1.3.48) was represented by 5 structurally distinct forms (Table 1).

**Table 1:** Distribution of non-homologous isofunctional enzymes among enzyme classes

Enzyme class	Enzyme nodes in ENZYME^a^	Enzyme nodes in KEGG^a^	Sequences with EC numbers	EC nodes with analogous enzymes		Fraction of the EC nodes
					
				Two classes of enzymes	Three or more classes	
Oxidoreductases (EC 1)	1343 (625)	575	98,166	31	5	5.8%
Transferases (EC 2)	1296 (683)	625	150,596	24	2	3.8%
Hydrolases (EC 3)^c^	930^c ^(441)	427^c^	81,538	76	11	19.7%^d^
Lyases (EC 4)	469 (235)	210	45,074	15	3	7.7%
Isomerases (EC 5)	177 (106)	100	30,429	15	2	16.0%^e^
Ligases (EC 6)	148(97)	104	44,829	1	0	1.0%

Total	4,363 (2,187)	2,041	450,632	185		4.4%

a - Only the EC nodes containing all 4 digits were considered; the numbers of EC nodes with at least one assigned protein sequence are shown in parentheses

b - From the in KEGG database, based on a set of 718 complete genomes

c - Peptidases (EC 3.4.x.x) were excluded from the analyzed set

d - EC nodes with analogous enzymes are overrepresented (p < 1 × 10^-12^)

e - Overrepresentation of EC nodes with analogous enzymes is not statistically significant (p ~ 0.08).

Of the previously reported 16 EC nodes corresponding to apparent NISE, where the three-dimensional (3D) structures were available for both forms, one case, β-galactosidase, EC 3.2.1.23, proved to be in error as catalytic domains of both forms (PDB entries 1BGL and 1GOW, respectively) had the TIM-barrel [βα)_8_] fold. Among the 18 additional cases of candidate NISE, prediction of distinct structural folds for two enzyme forms proved correct for 15 pairs. In two instances, the two isoforms turned out to possess the same fold, and one case (protochlorophyllide reductase, EC 1.3.1.33) had to be eliminated because the light-dependent and light-independent forms of this enzyme (PDB entries 3MIN and 1HDU, respectively) employ different electron donors and so, technically, catalyze different reactions.

Altogether, in 28 cases from the original list of 105 (predicted) NISE, the purported unrelated enzymes proved to belong to the same fold and even the same structural superfamily (Additional file 1, Table S1). Some of these cases revealed interesting evolutionary histories, such as, for example, the aforementioned circular permutation in the glutathione synthase (EC 6.3.2.3) structure [12] or the early divergence of the prokaryotic and eukaryotic versions of the FAD synthetase (EC 2.7.7.2) that was recently analyzed in detail by Grishin and coworkers [17]. For these enzymes, despite their apparent common origin, different isoforms showed little sequence similarity to each other, so their homology could be recognized only through structural comparisons. For the rest of the 28 "failed" cases, thanks to the improvements in sequence analysis methods and the growth in the number of diverse protein sequences in the public databases, sequence searches revealed significant similarity and common motifs suggesting a common origin for the respective isoforms.

Several additional pairs of enzymes failed to qualify as NISE after they were found to catalyze different reactions. For example, HAD1_PSEUC and HADD_PSEPU, two 2-haloalkanoate dehalogenases from *Pseudomonas *sp. (PDB entries 1QQ5 and 3BJX, respectively), although initially assigned to the same EC 3.8.1.2, exhibit different stereo-specificities, which prompted assignment of the latter form to the new EC 3.8.1.9. Likewise, two ubiquitin thiolesterases, represented by UBP5_HUMAN and STALP_HUMAN (PDB entries 2G43 and 2ZNR, respectively), despite having the same EC 3.1.2.15, actually possess distinct activities, cleaving polyubiquitin chains linked, respectively, through Lys-48 and Lys-63 residues of ubiquitin [18]. In two instances, erroneous assignments of enzyme pairs as NISE were due to the heteromeric, multi-subunit structures of one or both isoforms. For example, yeast sulfate adenylyltransferase MET3_YEAST (EC 2.7.7.4) is structurally unrelated to the CysN subunit of the sulfate adenylyltransferase from *E. coli*, but is closely related to the CysD subunit of the same enzyme. Finally, despite all the effort to include only proteins with proven enzymatic activity [6], one of the 105 cases included a wrong entry, putative catechol oxidase DXA2_DROME [19], which was later re-annotated as the 26S proteasome regulatory subunit 3 (UniProt entry P25161, PSMD3_DROME). Erroneous assignments of the last two kinds, those caused by the presence of multiple subunits and those caused by experimental errors, are a common problem complicating any search for NISE; the only remedy seems to be a careful case by case analysis (see below).

### New approaches to the identification of non-homologous isofunctional enzymes

To obtain a comprehensive list of NISE (Additional file 2, Table S2), we combined several search strategies to connect protein sequences with the reactions they catalyze. First, all Swiss-Prot entries with the same EC number were clustered by sequence similarity and those EC nodes that yielded more than one cluster were further analyzed. As part of this approach, we examined the EC nodes that have been assigned two or more profiles in the PRIAM database [13].

**Figure 1:**
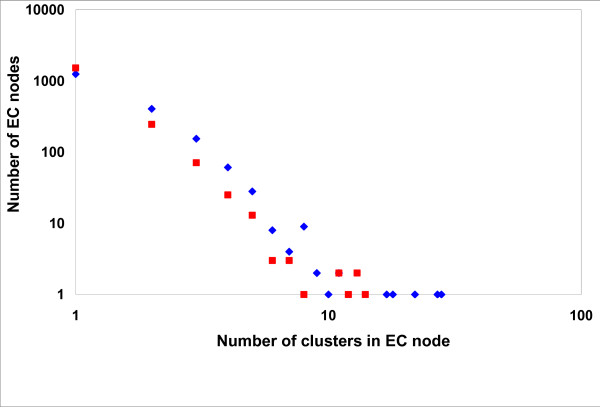
**Clustering results for the enzymes with four-digit EC numbers represented in the ENZYME (squares) and KEGG (diamonds) data sets**.

To cover new enzyme sequences emerging from whole-genome sequencing efforts, the same approach was applied to the KEGG database [20], which assigns EC numbers to genome-derived protein sequences using a custom algorithm [21,22]. The statistics of protein clustering are presented in Figure 1 and Tables S3 and S4 in Additional File 3. The second approach used the KEGG collection of enzymatic reactions to identify enzymes that, while having different substrate specificities and hence different EC numbers, are capable of catalyzing the same reaction. To this end, we examined the clustered protein sequences assigned to any reaction that corresponded to two or more EC nodes. All cases where the same reaction was associated with more than one EC node were manually analyzed.

### The new compendium of non-homologous isofunctional enzymes

The final list of NISE, obtained after a detailed case-by-case analysis, includes 140 confirmed EC nodes where the presence of two or more distinct forms is supported by a comparison of their 3D structures that revealed different folds (Additional file 2, Table S2). In additional 45 EC nodes, pairs of candidate NISE were inferred to be structurally distinct on the basis of the analysis of their predicted structures (Additional file 2, Table S2). In one instance, neither of the two forms of 4-methyl-5-(2-hydroxyethyl)thiazole phosphate synthase (commonly referred to as thiazole synthase) described in the literature (bacterial ThiG and the yeast THI4) had been assigned an EC number [11].

In addition, we identified 26 tentative cases for which the NISE status, although likely, could not be confirmed (see Additional file 2, Table S2). Again, one case came from literature searches: the EC number 2.7.1.31, initially assigned to glycerate 3-kinases, was found to include also glycerate kinases that produce 2-phosphoglycerate [23,24]. Finally, there were 10 cases where two structurally unrelated enzymes could catalyze the same biochemical reaction but had been assigned different EC numbers, usually based on differences in substrate specificity. These pairs of enzymes are listed in a separate section of the Table S2 (Additional file 2) but were not included in any further analyses.

**Figure 2:**
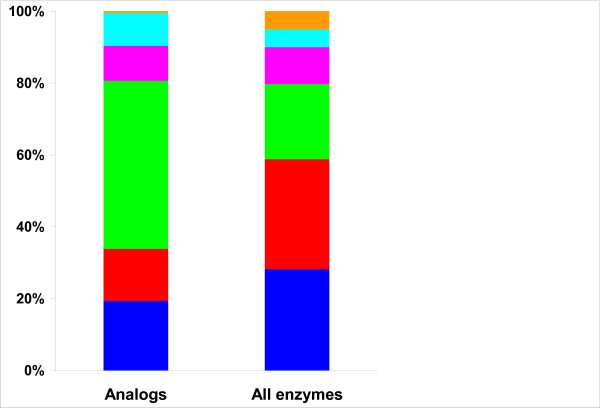
**Distribution of non-homologous isofunctional enzymes among various enzyme classes**. The fraction of EC nodes of each class that contain NISE (left column) as compared with the fraction of EC nodes from each class in the complete set of 2181 EC nodes that containing more than one protein sequence (right column). The EC classes are ordered from the bottom: EC1, blue; EC2, red; EC 3, green; EC4, magenta; EC5, cyan; EC6, orange.

The 186 EC nodes with confirmed or predicted NISE represent approximately 8.5% of all analyzed EC nodes; only 73 of these were present in the previously published list [6]. As noted previously [6], NISE could be found in all 6 classes of enzymes recognized in the EC (Table 1). Hydrolases (EC 3) were overrepresented among NISE whereas transferases (EC 2) were underrepresented (Table 1, Figure 2). The fractions of NISE from the other four enzyme classes were as expected considering the total counts of the EC nodes in those enzyme classes (Additional file 3, Table S5).

The majority of the NISE were represented by two unrelated forms, although some were found (or predicted) to possess three or more distinct forms (Table 2). The greatest number of NISE represented by three or more different structures was found among hydrolases, followed by oxidoreductases (Table 1). Among glycoside hydrolases, catalytic domains of cellulase and licheninase are represented by the same 3 folds which in the SCOP database [25] are referred to as, respectively, TIM beta/alpha-barrel, alpha/alpha toroid and concanavalin A-like lectins/glucanases. Cellulase was also represented by at least two additional folds (Table 2). Other enzymes with multiple non-homologous isoforms include acid phosphatase, protein-tyrosine-phosphatase, adenylate cyclase, and DNA-(apurinic or apyrimidinic site) lyase, as well as such enzymes as catalase, peroxidase, peroxiredoxin, chloride peroxidase, and superoxide dismutase that participate in defense against reactive oxygen species (ROS) (Table 2).

**Figure 3:**
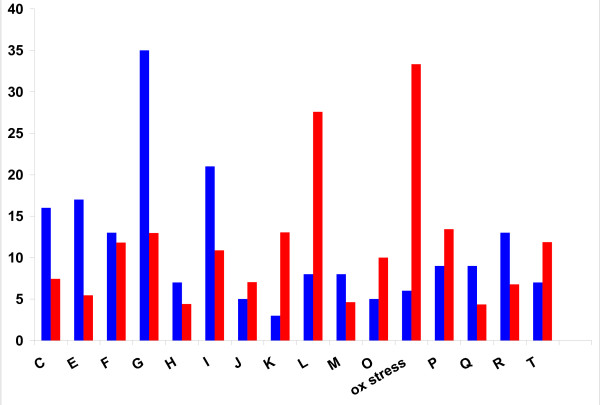
**Distribution of non-homologous isofunctional enzymes by COG functional categories**. For each category, the left (blue) column shows the absolute number of EC nodes with NISE, and the right (red) column shows this number as a percentage of all EC nodes assigned to that category. COG functional categories are as follows: C - energy production and conversion, E - amino acid transport and metabolism, F - nucleotide transport and metabolism, G - carbohydrate transport and metabolism, H - coenzyme metabolism, I - lipid metabolism, J - translation, ribosomal structure and biogenesis, K - transcription, L - DNA replication, recombination and repair, M - Cell envelope biogenesis, outer membrane, O - posttranslational modification, protein turnover, chaperones, ox stress - defense from oxidative stress, P - inorganic ion transport and metabolism, Q - secondary metabolites biosynthesis, transport and catabolism, R - general function prediction only, T - signal transduction.

Functional distribution of the NISE among the COG functional categories [26] reflected the abundance of COGs in each category (Figure 3). The only statistically significant deviation from this pattern was the overrepresentation of enzymes involved in defense against oxidative stress and in DNA repair (Additional file 3, Table S6). The greatest number of confirmed and predicted NISE was associated with carbohydrate metabolism (Figure 3). Other functional classes that were relatively well represented among NISE included amino acid metabolism, lipid metabolism, nucleotide metabolism, and energy production and conversion. Non-homologous isofunctional enzymes were found in many metabolic pathways such as glycolysis/gluconeogenesis, purine biosynthesis, and pentose phosphate pathway. However, the distribution is patchy, that is, NISE are scattered among different metabolic pathways, with not a single pathway identified in which all reactions would be catalyzed by multiple non-homologous isoforms.

**Table 2:** Enzymes with multiple structurally distinct, non-homologous isoforms

Enzyme name (EC)	Example (SwissProt)	Structure (PDB)	Fold names in SCOP (abbreviated)
Acid phosphatase (EC 3.1.3.2)	PPA6_HUMAN	1NDH	Phosphoglycerate mutase-like
	APHA_ECOLI	1N8N	HAD-like
	PPA_ZYMMO	1D2T	Acid phosphatases
	PPA5_HUMAN	1UTE	Metallo-dependent phosphatases
	PPAC_HUMAN	5PNT	Phosphotyrosine protein phosphatases I
	PHOA_PENCH	n/a	n/a

Cellulase (EC 3.2.1.4)	GUNA_CLOCE	1EDG	TIM beta/alpha-barrel
	GUNA_PSEFL	1UT9	Alpha/alpha toroid
	GUN1_STRHA	2BOD	7-Stranded beta/alpha barrel
	GUN_ASPAC	1KS4	Concanavalin A-like lectins/glucanases,
	GUNM_CLOTM	2FVG	Phosphorylase/hydrolase-like
	GUNE_RUMFL	1L0H	*Predicted acyl carrier protein-like*

Superoxide dismutase (EC 1.15.1.1)	SODF_ECOLI	1ISA	Fe,Mn superoxide dismutase (SOD)
	SODC_ECOLI	1ESO	Immunoglobulin-like beta-sandwich
	SODN_STRSO	1Q0D	Four-helical up-and-down bundle
	NEC1_NICLS	2ET7	Double-stranded beta-helix

Phosphoprotein (Ser) phosphatase (EC 3.1.3.16)	PRP1_ECOLI	1G5B	Metallo-dependent phosphatases
	PRPC_BACSU	1TXO	PP2C-like
	CTDS1_HUMAN	1TA0	HAD-like
	DUS19_HUMAN	1M3G	Phosphotyrosine protein phosphatases II

Protein-tyrosine phosphatase (EC 3.1.3.48)	PTPA_STRCO	1U2P	Phosphotyrosine protein phosphatases I-like
	PTPRD_HUMAN	2FH7	Phosphotyrosine protein phosphatases II
	MPIP3_HUMAN	1QB0	Rhodanese/Cell cycle control phosphatase
	YWQE_BACSU	2ANU	*Predicted 7-stranded beta/alpha barrel*
	EYA3_MOUSE	1JUD	*Predicted HAD-like*

DNA-(apurinic or apyrimidinic site) lyase (EC 4.2.99.18)	END3_ECOLI	2ABK	DNA-glycosylase
	APEX1_HUMAN	1E9N	DNase I-like
	APN1_YEAST	1QTW	TIM beta/alpha-barrel
	FPG_ECOLI	1K82	MutM-like DNA repair proteins

Adenylate cyclase (EC 4.6.1.1)	CYA1_HUMAN	1CS4	P-loop NTPases
	O69199_AERHY	2ACA	CYTH-like phosphatases
	CYAA_BORPE	1YRU	EF Hand-like
	CYAA_ECOLI	n/a	n/a

Inorganic pyrophosphatase (EC 3.6.1.1)	IPYR_ARATH	1TWL	OB-fold
	PPAC_BACSU	1WPM	DHH phosphoesterases
	PPAX_BACSU	2HDO	HAD-like
	*AVP1_ARATH*	n/a	*Integral membrane protein, H*^+^*-transporting*

Catalase (EC 1.11.1.6)	CATA_HUMAN	1QQW	Heme-dependent catalase-like
	CATA_ECOLI	2FXG	Heme-dependent peroxidases
	MCAT_LACPL	1JKU	Ferritin-like

Peroxidase (EC 1.11.1.7)	PRDX6_MOUSE	1PRX	Thioredoxin fold
	PERM_HUMAN	1MYP	Heme-dependent peroxidases
	YCDB_ECOLI	2d3q	Ferredoxin-like

Chloride peroxidase (EC 1.11.1.10)	PRXC_PSEPY	1A88	Alpha/beta-hydrolases
	PRXC_CURIN	1VNC	Acid phosphatase
	PRXC_CALFU	2CPO	EF Hand-lik

Peroxiredoxin (EC 1.11.1.15)	TDXH_AERPE	2E2G	Thioredoxin fold
	AHPD_MYCTU	1KNC	AhpD-like
	OSMC_ECOLI	1NYE	OsmC-like

Licheninase (EC 3.2.1.73)	GUB_NICPL	2CYG	TIM beta/alpha-barrel
	GUB_BACSU	1GBG	Concanavalin A-like lectins/glucanases,
	GUB_BACCI	1V5C	Alpha/alpha toroid

The over-representation of NISE in pathways involved in protection against ROS appears to support the idea of Doolittle who referred to some of the NISE as "second edition" enzymes [27] that evolved relatively late as adaptations to new environments such as, for example, the oxygen-rich atmosphere.

### Structural features of non-homologous isofunctional enzymes

Inspection of the structural properties of NISE reveals a large variety of fold combinations that result in structurally distinct isoforms (Additional file 2, Table S2). However, several folds, most conspicuously, the TIM (β/α)_8_-barrel fold that is seen primarily among hydrolases, were statistically over-represented among the NISE (Table 3 and Additional file 3, Table S7). The second most common fold was the NAD(P)-binding Rossmann fold that is seen primarily among oxidoreductases but is not statistically over-represented because of its overall high abundance. In 11 instances, pairs of NISE consisted of representatives of the TIM-barrel and Rossmann folds. The over-representation of TIM-barrels among analogous enzymes is consistent with the extraordinary biochemical versatility of this symmetrical domain resulting in its ability to catalyze a broad variety of biochemical reactions [28,29].

In contrast, other fold combinations rarely form pairs of NISE (Additional file 2, Table S2). Parallel utilization of the same folds is seen mostly when their representatives catalyze similar reactions. For example, non-homologous isofunctional L-lactate dehydrogenases (EC 1.1.1.27) and malate dehydrogenases (EC 1.1.1.37) feature the same fold pairs, namely, the L-sulfolactate dehydrogenase-like fold and a combination of the NAD(P)-binding Rossmann-fold with a LDH C-terminal domain. The same pattern is seen among proteins that participate in defense against oxidative stress. In particular, the heme-dependent peroxidase fold is responsible for one of the two (or three) non-homologous isoforms of catalase, peroxidase and cytochrome c peroxidase, whereas the ferredoxin-like fold is found in a peroxidase and a heme oxygenase. These observations are compatible with our previous conclusion [6] that the most common route for the origin of NISE enzymes is recruitment of an existing enzyme that catalyzes a closely related reaction through a relatively minor change in substrate specificity or the catalytic mechanism (see also [30]).

**Figure 4:**
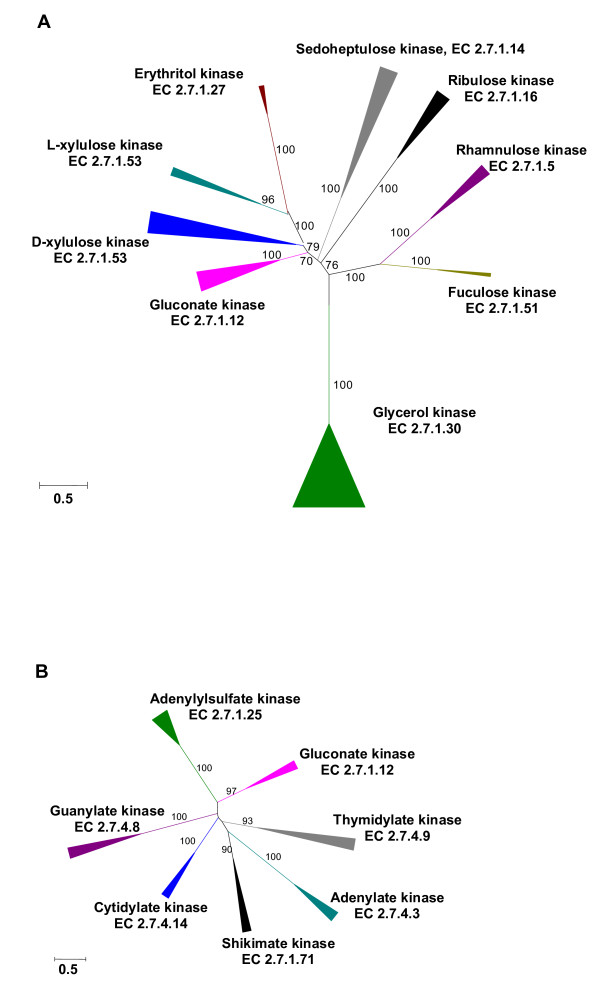
**Phylogenetic trees of two non-homologous gluconate kinases and related enzymes**. A. Carbohydrate kinases of the FGGY family. B. Carbohydrate and nucleoside kinases of the AAA family. Annotation of each group includes the functional assignment (the substrate and EC node) of the experimentally characterized member(s) and the phylogenetic distribution of its close homologs.

An example of such an evolutionary development is seen on Figure 4, which shows the phylogenetic tree of two carbohydrate kinase families that include non-homologous gluconate kinases (EC 2.7.1.12). One of these families (Figure 4A), referred to as the FGGY family of carbohydrate kinases in Pfam [31], includes enzymes with experimentally demonstrated kinase activities towards various C3-C7 substrates, such as glycerol (EC 2.7.1.30), erythritol (EC 2.7.1.27), rhamnulose (EC 2.7.1.5), ribulose (EC 2.7.1.16, 2.7.1.47), D-xylulose (EC 2.7.1.17), L-fuculose (EC 2.7.1.51), L-xylulose (EC 2.7.1.53), and sedoheptulose (EC 2.7.1.14). Glycerol kinase has the simplest substrate in this group and catalyzes a reaction of glycerol metabolism that is common to bacteria, archaea, and eukaryotes; this enzyme is widespread in representatives of all three domains of life. Other kinases of this family participate in various reactions of sugar metabolism and show more narrow phyletic distributions. Glycerol kinase might represent the ancestral form that subsequently evolved to accommodate new substrates while retaining the overall structural fold and the reaction mechanism. The second form of gluconate kinase comes from an even larger, P-loop kinase family [32] (Figure 4B) which includes, among others, shikimate kinase (EC 2.7.1.71, see below), phosphoribulokinase (EC 2.7.1.19), adenylylsulfate kinase (EC 2.7.1.25), and a variety of nucleotide/nucleoside kinases, such as cytidylate kinase (EC 2.7.4.14), guanylate kinase (EC 2.7.4.8), adenylate kinase (EC 2.7.4.3), and thymidylate kinase (EC 2.7.4.9). It appears that in each case, gluconate kinase was recruited from a family of enzymes (kinases) that catalyze the same chemical reaction (phosphorylation) with closely related substrates.

**Table 3:** Protein folds most commonly found among non-homologous isofunctional enzymes

Fold	EC1	EC2	EC3	EC4	EC5	EC6	Total
Total number of folds per EC class	46	39	87	34	36	2	

TIM beta/alpha-barrel	10	2	29	7	3	-	51*
NAD(P)-binding Rossmann-fold	20	-	5	2	1	-	28
Alpha/beta hydrolases	1	3	8	-	-	-	12*
Ribonuclease H-like motif	-	3	9	-	-	-	12*
Metal-dependent phosphatases	-	-	11	-	-	-	11*
Alpha/alpha toroid	-	-	6	3	1	-	10*
Flavodoxin-like	4	1	4	1	-	-	10
P-loop containing NTPases	1	7	-	1	-	-	9
HAD-like	-	-	7	1	-	-	8
Ferredoxin-like	3	3	-	1	1	-	8

* - These folds are over-represented among the NISE (p < 0.05).

EC1, oxidoreductases; EC2, transferases; EC3, hydrolases; EC4, lyases; EC5, isomerases; EC6, ligases.

### Phyletic distribution of non-homologous isofunctional enzymes

The distribution of most NISE across the three domains of life is patchy and devoid of obvious regularity. Nevertheless, several NISE do show complementary phyletic patterns (Table 4), where the two isoforms are restricted to non-overlapping or minimally overlapping subsets of lineages. For example, the recently described "archaeal" form of shikimate kinase, a member of the GHMP kinase family [33], is indeed found exclusively in archaea, whereas the well-known "bacterial" form of this enzyme, a member of the P-loop NTPase superfamily, is found in bacteria and eukaryotes (Table 4).

**Table 4:** Distinct phyletic patterns of non-homologous isofunctional enzymes

Enzyme (EC)	Examples	Instances in bacteria (out of 607)	Instances in archaea (out of 48)	Instances in eukaryotes (out of 63)
Superoxide dismutase (EC **1.15.1.1**)	*SODF_ECOLI*	526	23	62
	*SODC_ECOLI*	249	3	47
	*SODN_STRSO*	32	0	1
	*NEC1_NICLS*	6	0	3

Heme oxygenase (EC **1.14.99.3**)	*HMOX1_HUMAN*	134	0	41
	*ISDI_STAAR*	36	0	0

Shikimate kinase (EC **2.7.1.71**)	*AROL_ECOLI*	553	9	28
	*AROK_METJA*	0	39	0

Diacylglycerol kinase (EC **2.7.1.107**)	*KDGL_ECOLI*	404	0	0
	*DGKG_HUMAN*	23	0	59

Fructose bisphosphatase (EC **3.1.3.11**)	*F16PA_ECOLI*	268	5	54
	*F16P_BACSU*	307	4	2
	*Q8U359_PYRFU*	19	37	0

Carbonic anhydrase (EC **4.2.1.1**)	*CYNT_ECOLI*	414	17	35
	*CAH_METTE*	211	32	10
	*CAH1_HUMAN*	88	0	40

Glucose-6-phosphate isomerase (EC **5.3.1.9**)	*G6PI_HUMAN*	547	10	58
	*G6PI_THELI*	11	13	0

Lysine---tRNA ligase (EC **6.1.1.6**)	*SYK1_ECOLI*	551	15	63
	*SYK_AERPE*	91	36	0

The data are from the ortholog tables in the KEGG database [20], supplemented with the results of iterative BLAST searches against the NCBI's Reference Sequence database [56].

Similarly, the bacterial and eukaryotic forms of diacylglycerol kinase appear primarily in the respective lineages and are missing from all archaeal genomes sequenced to date. The exceptions to this pattern include the presence of the soluble "eukaryotic" form in a limited number of bacteria, mostly firmicutes, and the discovery of the bacterial-like membrane-embedded form encoded in the cyanobacterium-like plastid of the cercozoan "green amoeba" *Paulinella chromatophora *and in the unfinished genome of castor bean (in the latter case, a bacterial contamination remains to be ruled out). Several other enzyme forms, such as class I lysyl-tRNA synthetase and cupin-type glucose-6-phosphate isomerase, originally described in archaea [34-37], are also found in certain lineages of bacteria (Table 4).

A comparison of the phyletic distributions of the enzymes that participate in defense against ROS revealed an abundance of distinct forms of these enzymes in bacterial and eukaryotic (particularly plant) genomes as opposed to a much lower diversity in archaea (Additional file 3, Table S7). The most common iron-dependent superoxide dismutase is universally present in bacteria, eukaryotes, and is also seen in some archaea. The copper/zinc-dependent form of superoxide dismutase is widespread in bacteria and eukaryotes as well, but among archaea its presence is limited to a few aerobic halophiles. The third form, nickel-dependent superoxide dismutase, is unique to bacteria, and is found primarily in actinobacteria and cyanobacteria, and accordingly, in plastids of green algae and diatoms. The fourth superoxide dismutase, a manganese-dependent cupin-type enzyme related to oxalate oxidase [9], appears to be encoded only in land plants. Likewise, most archaea encode a single form of peroxidase and chloride peroxidase (Additional file 3, Table S8).

### Non-homologous isofunctional enzymes and genome size

**Figure 5:**
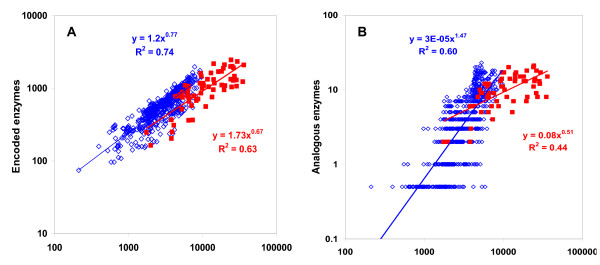
**Dependence between the genome size (the total number of encoded proteins) and the number of encoded enzymes**. A. The total number of encoded enzymes with assigned four-digit EC numbers. B. The number of encoded non-homologous isofunctional enzymes. Prokaryotic genomes are indicated with diamonds, eukaryotic genomes are indicated with squares. The best fit lines were calculated separately for prokaryotes (blue) and eukaryotes (red).

Previously, we analyzed the involvement of NISE in the key reactions of central metabolism [3,10] and concluded that the presence of NISE correlates with the genome size: microorganisms with small genomes typically encode a single form of any enzyme whereas organisms with larger genome size often carry the genes for two or more non-homologous isoforms [6]. These observations were confirmed on the larger set of NISE and the much larger set of complete genome sequences analyzed here (Figure 5). The total number of predicted enzymes with assigned EC numbers positively scales with the genome size, with an exponent of approximately 0.7, slightly lower than reported for metabolic enzymes in the studies on universal scaling behavior of different functional classes of proteins [38,39]; the exponent for prokaryotes is slightly greater than that for eukaryotes but the difference is not statistically significant (Figure 5A). The number of NISE pairs encoded in any given genome scales with the genome size substantially steeper than the total number of enzymes in prokaryotes but somewhat less steeply in eukaryotes (Figure 5B). These observations seem to reflect, mostly, the metabolic versatility of free-living bacteria, particularly, those that inhabit complex environments. In these organisms, the partial redundancy and specialization of enzymes appear to grow faster than linearly with the genome size.

## Discussion

### Functional, structural and evolutionary patterns of non-homologous isofunctional enzymes

This study expanded the previously delineated list of NISE [6] and, more importantly, put the phenomenon of utilization of multiple, non-homologous enzymes for catalysis of the same reaction on a firm structural footing. In the previous study, the sets of NISE, for which the existence of two or more distinct folds (the ultimate proof of the lack of homology), could be demonstrated directly was a small minority but this fraction changed to majority in the present study thanks to the advances of structural biology in general and structural genomics in particular. The present analysis detected non-homologous isoforms for approximately 8.5% of the enzymes included in the EC system. This is the low bound for the spread of NISE among enzymes because a considerable number of proteins that show varying degrees of evolutionary conservation but have not been biochemically characterized [40] are likely to be non-homologous isoforms of known enzymes. Thus, the fraction of non-homologous isoform sets among enzymes is likely to be close to 10%, by any account a widespread, substantial phenomenon.

We examined the distributions of NISE across several planes of biological diversity including classes of enzymes, biochemical pathways, protein folds, and phylogenetic lineages. The overall conclusion that does not seem to be particularly surprising (see, e.g. [41]) is the patchiness of the distribution of NISE and the paucity of strong trends. Nevertheless, several distinct patterns are supported statistically and deserve attention.

Non-homologous isofunctional enzymes are notably more common among hydrolases than in other EC classes. Evolutionary invention of unrelated catalysts for the same reaction seems to be relatively easy in this class of enzymes because one of their substrates is the universal small molecule (H_2_O), and the hydrolysis reaction typically does not require any coenzymes.

In the structural space, TIM-barrels are significantly over-represented among the NISE. This observation is compatible with the remarkable biochemical versatility of the TIM-barrel stemming from its symmetry that allows accommodation of different activities and substrate specificities through limited structural change.

Non-homologous isofunctional enzymes are represented in a great variety of biochemical pathways and systems, typically, in one or two reactions of a pathway. Against this overall patchy background, the excess of NISE in systems of defense against ROS is remarkable. It is tempting to speculate that in this case the emergence of analogous enzymes was driven by a powerful selection pressure in the face of the rapid oxygenation of the earth atmosphere. This pressure apparently triggered independent evolution of several distinct solutions for the set of relatively simple reactions that are required for ROS detoxification.

Non-homologous isofunctional enzymes are found in all major lines of cellular life. However, they show a superlinear scaling with genome size in bacteria but not in eukaryotes and accordingly appear to be most abundant in biochemically complex bacteria with large genomes. Detailed phylogenomic analysis of individual sets of NISE reveals evidence of "evolutionary tinkering" [42] that is pervasive in genome evolution. Non-homologous isoforms of enzymes seem to be recruited from pre-existing enzymes with related activities and specificities following duplication or horizontal transfer (apparently, the principal route of innovation in prokaryotes [43,44], where NISE are most common) of the respective genes. The recurrence of certain folds, such as the TIM-barrel, the Rossmann-fold or the alpha/beta hydrolase domain, in the sets of NISE reflects the biochemical versatility of these domains because of which they are, in a sense, "pre-adapted" for evolution of NISE. For instance, if an alpha-beta hydrolase exists with a specificity for a particular hydrolytic reaction, it will only take a small modification of a TIM-barrel with a related hydrolytic activity to evolve a pair of NISE.

### Non-homologous isofunctional enzymes and enzyme classification

Starting from the first enzyme classification schema devised by Dixon and Webb [45] and adopted by the Enzyme Commission of the International Union of Biochemistry [46], "enzymes are principally classified and named according to the reaction they catalyze" [47]. Further, "a certain name designates not a single enzyme protein but a group of proteins with the same catalytic property". The EC code numbers consist of four elements which specify, respectively, the enzyme class, subclass (the bond broken or hydrolyzed, the group transferred, etc.), sub-subclass (usually the nature of the substrate), and the serial number of the enzyme in the respective sub-subclass [47]. For the past 50 years, this nomenclature provided a solid basis for biochemical research and was able to accommodate and classify a variety of newly discovered enzymes. The EC system proved to be an indispensable tool for largely automated assignments of enzymatic functions to the numerous protein sequences encoded in the sequenced genomes of various, often poorly studied, organisms [48]. However, it is important to note that this classification is function-based, not sequence- or structure-based, and a substantial number of EC nodes are still not assigned a single protein sequence [49] (see Table 1). The strict reliance on substrate specificity is also a cause for certain confusion when the EC numbers are used to map reactions on the metabolic map. As an example, oxidation of D-glucose to D-glucono-1,5-lactone, catalyzed by the NAD^+^-dependent glucose dehydrogenases (EC 1.1.1.118), could also be listed under alcohol dehydrogenase (EC 1.1.1.1) and aldose dehydrogenase (EC 1.1.1.121), as well as under NADP^+^-dependent (EC 1.1.1.47), FAD-dependent (EC 1.1.99.10), or PQQ-dependent (EC 1.1.5.2) glucose 1-dehydrogenase. The EC approach also becomes problematic when the substrates are complex (e.g. proteins) or unknown. This complication has been recognized and successfully dealt with in the case of peptidases (EC 3.4.x.x [50,51]) but remains a problem for various protein kinases and protein phosphatases, which are known to be highly specific for their targets but are currently lumped together under the same EC nodes. It is hardly surprising that phosphoprotein (Ser) phosphatase (EC 3.1.3.16) and protein-tyrosine phosphatase (EC 3.1.3.48) made our list of enzymes with the largest numbers of (predicted) unrelated folds (Table 2). The same problem is expected to arise in the case of "house-cleaning" enzymes that hydrolyze specific non-canonical NTPs and other cellular waste products [52] but are usually characterized based on their side activity towards canonical sugars and/or NTPs [53].

Non-homologous isofunctional enzymes add a further complication to the EC classification schema. Although the existence of alternative forms of fructose bisphosphate aldolase, phosphoglycerate mutase and superoxide dismutase has been known for many years, recent studies have led to a dramatic increase of the number of such cases [13,14], including many where alternative enzyme forms have been unequivocally shown to adopt distinct structural folds (Table 1 and Additional file 2, Table S2). According to the general rules of the EC classification, enzyme isoforms that catalyze the same reaction do not qualify for different EC nodes [47]. Nevertheless, it seems reasonable to consider expanding the EC system by officially recognizing the notion of a "class" within an EC node, such as, for example, superoxide dismutase (EC 1.15.1.1) class I, class II, and so on. We hope that the present study, along with other related projects [13,14], could help in this regard.

Despite the fuzzy character of certain EC nodes, such as acid phosphatase (EC 3.1.3.2) or NADH dehydrogenase (EC 1.6.99.3), the EC-based approach is an estimate of the lower bound of the number of NISE. This approach leaves out many enzymes that catalyze similar biochemical reactions but differ in the nature of the phosphoryl donor (ATP, GTP, or pyrophosphate) or the electron acceptor (NAD^+^, NADP^+^, or both) and accordingly have been assigned different EC numbers. Conversely, it cannot be ruled out that detailed study of reactions catalyzed by enzymes that are currently assigned the same EC number reveals differences in substrates, cofactors or mechanisms that will eventually justify their classification to different EC nodes. Thus, not surprisingly, the NISE represent a moving target although we believe that the above estimate of approximately 10% NISE among enzymatic reactions is reasonably robust.

## Conclusions

Sets of evolutionarily unrelated, non-homologous isofunctional enzymes were detected for a substantial fraction (up to 10%) of biochemical reactions, and adequate description of these enzymes is important for the practical tasks of metabolic reconstruction and enzyme classification. Beyond this practical importance, NISE represent a major evolutionary phenomenon: their existence shows that, at least, for numerous and diverse biochemical problems, evolutionarily unrelated solutions can evolve. A crucial corollary of this finding is that the role of convergence in the evolution of proteins is at best very limited, and whenever enzymes with same fold catalyze the same reaction, they most likely have a common origin, even in the absence of significant sequence similarity. Conversely, an interesting subject for structural and functional studies is the search for subtle similarities between NISE that might allow them to accommodate the same substrates and catalyze the same reaction.

## Methods

Identification of NISE was performed using 3 methods. The principal approach again relied on the Enzyme Commission (EC, [47] numbers, where each complete EC number (node) specifies one particular biochemical reaction. Accordingly, NISE were identified as pairs of enzymes that had been assigned the same EC number but showed no detectable sequence similarity to each other. The second approach focused on apparently unrelated proteins with different EC numbers that were marked as catalyzing the same biochemical reaction in the KEGG database [20]. In addition, potential NISE were identified through text searches using keywords "analogous enzymes", "enzyme class" etc.

The EC-based analysis used protein sequences from the ENZYME [54] and KEGG databases. The KEGG database was used to track enzyme distribution in 718 completely sequenced genomes from 63 eukaryotes, 48 archaea and 607 bacteria (for the complete list, see Additional file 3, Table S9). The ENZYME database [55] was used as the source of information about the enzymatic activities demonstrated for proteins from organisms that might not have completely sequenced genomes.

All GenBank gene identification numbers (GIs) for proteins with assigned four-digit EC numbers in the KEGG and ENZYME databases were collected and their sequences were extracted from the NCBI Protein database [56]. Sequences containing fewer than 60 amino acid residues were discarded as these typically were fragments of proteins. Sequences that had been assigned two or more EC numbers were included in the analyzed set under each of these EC numbers. The initial combined set contained 2637 unique EC nodes (excluding proteases, see Additional file 3, Table S3). The EC nodes represented by single proteins (456 in total) were removed from the analyzed set, as were 4 EC nodes that represented large multi-subunit complexes (DNA-directed DNA and RNA polymerases, NADH dehydrogenase complex, and H^+^-transporting ATPase). The final set used for sequence clustering included 2177 EC nodes. BLASTP searches [57] were performed for each protein with a particular EC number against all other proteins with the same EC number. Single-linkage clustering was performed with the expectation value cut off of 0.01. The EC nodes were then sorted according to the number of sequence clusters associated with each of them. Most of the EC nodes (1397 of the 2177) were represented by single sequence clusters and were not analyzed further. At the next step, we identified and removed from further analysis 106 EC nodes that were represented by two or more sequence clusters but each cluster corresponded to a separate subunit of a heteromeric multi-subunit enzyme, as judged by UniProt, COG and/or CDD annotations of representatives of these clusters. The resulting set of candidate NISE consisted of 674 EC nodes represented by two or more sequence clusters. Members of every single-linkage cluster were searched against the PDB sequence subset of the NCBI protein database [56], the first three hits were collected and checked for SCOP fold annotations, where available. Clusters whose representatives produced reliable hits into PDB entries within the same SCOP fold were discarded and the corresponding EC nodes were removed from the analyzed set. Those sequence clusters that contained proteins with the PDB hits assigned to different SCOP folds (or those with no reliable PDB hits) were searched against Swiss-Prot and the results were manually analyzed.

In the course of the manual analysis, the EC nodes were assumed to harbor NISE if representatives of different single-linkage clusters for the same EC node had reliable hits (i) to the UniProtKB\SwissProt [58] and ENZYME [54] database entries with verified (and identical) enzymatic activity, and (ii) to the PDB entries that the SCOP [25] and/or CATH [59] databases assigned to different folds. The sequences without reliable PDB hits were assigned to SCOP folds using the SUPERFAMILY database [60]. Those PDB entries not listed in SCOP were assigned to SCOP folds using the Sequence-structure matching (SSM) tool [61].

The final refinement of the data set included manual elimination of protein sequences that did not satisfy the criteria for NISE, primarily proteins with apparently incorrectly assigned EC numbers or undocumented enzymatic activity (see [6] for additional details). The patterns of taxonomic distribution of the NISE were obtained from the KEGG assignments for 718 sequenced genomes [20].

The second approach was used to identify NISE that can catalyze the same biochemical reaction but have been assigned different EC numbers based on differences in substrate specificity (broad versus narrow), cofactor requirement, or physico-chemical parameters, e.g., the optimal pH. We downloaded the list of all 6564 KEGG reactions and selected those 308 of them that could be catalyzed by two or more enzymes with different four-digit EC numbers supported by at least a single protein sequence. For these EC numbers, lists of all structures (PDB IDs assigned in KEGG) were collected whenever possible and checked for fold assignments in the SCOP database. For the EC nodes without structural assignments in KEGG, selected representatives of single-linkage clusters obtained previously for each EC node were searched against PDB, and SCOP folds were assigned using the SUPERFAMILY database. Pairs of EC nodes whose representatives were assigned different folds but experimentally demonstrated to catalyze the same enzymatic reactions were added to the list of analogous enzymes.

Statistical significance of over- or under-representation of enzyme classes, functional groups and folds among the NISE was evaluated using the chi-square test (p < 0.05 was considered significant). Maximum-likelihood phylogenetic trees were constructed using the TreeFinder program [62] by optimizing a default starting tree constructed using the neighbor-joining method with the Whelan and Goldman (WAG) empirical model of substitutions [63]. The complete listing of the NISE identified in this study (Additional file 2, Table S2) is available online at http://www.ncbi.nlm.nih.gov/Complete_Genomes/AnalEnzymes.html.

## List of abbreviations

EC: Enzyme Commission; 3D: three-dimensional.

## Competing interests

The authors declare that they have no competing interests.

## Authors' contributions

All authors analyzed the data, wrote and revised the paper and approved the final version of the manuscript.

## Reviewers' comments

### Reviewer 1: Andrei Osterman, Bioinformatics and Systems Biology Program, Sanford-Burnham Medical Research Institute, La Jolla, California, USA

Reviewer 1

An insightful and thorough study by Omelchenko et al. brings our attention to one of the most fascinating aspects of enzyme evolution, prolific existence of protein families encoding non-homologous isofunctional enzymes. Authors provided a new census of well-documented cases of such "analogous" enzymes revealing that this phenomenon is much more widespread than could have been expected in the early days of genomics. This new study was largely facilitated by advents of genome sequencing and structural genomics, which helped to correct some of the conclusions in their earlier analysis, especially with respect to distant homologs. A well-dosed combination of the elegantly designed automated analysis with manual case-by-case investigation allowed authors to generate a unique and highly useful dataset provided in the Supplementary Materials. By applying stringent criteria (distinct folds) Omelchenko *et al*. concluded that at least 1/10 of all enzymes with presently assigned complete EC numbers could have emerged in evolution more than once. Such a high level of evolutionary redundancy is quite remarkable. Another notable conjecture based on the detailed analysis of this data is that the evolution of analogous enzymes appears to be largely driven by recruitment from distinct structural families (folds) featuring similar reactions. It provides another vivid illustration of the *patchwork pathway evolution *hypothesis of R. Jensen [41]. Abundance of non-homologous isozymes in prokaryotes was shown to correlate with the genome size and their distribution among various folds reflects functional versatility of popular folds (such as TIM barrel and Rossmann fold). This analysis sets a stage for further analysis of interrelationships between evolutionary redundancy and the types of catalyzed chemical reactions. Overall, this study contributes to our appreciation of the abundance of alternative solutions for the same or similar functional tasks that have emerged in course of evolution. In addition to its fundamental importance, this awareness as well as the captured specific knowledge would impact a number of applications in genomics (functional annotations and metabolic reconstruction), bioengineering (directed enzyme/pathway evolution) and drug discovery (identification of selective drug targets).

**Authors' response: **We thank the reviewer for these kind comments

Reviewer 1

"Analogous enzymes": outside of juxtaposition (analogous vs homologous) this term may be somewhat misleading. For example, if I'd hear that "these two enzymes are analogous" (outside of context of your paper title, which is helpful), I would think that the meaning is that these are two enzymes (homologous or not) catalyzing similar (analogous) but not identical reactions (e.g. glucokinase and mannokinase). What you mean in fact is "non-homologous isofunctional" enzymes (or "non-homologous isozymes"). You actually made a step towards better term in "analogous isoforms".

***Authors' response: **We agree. Actually, we found these comments so insightful and relevant that the phrase 'analogous enzymes' was replaced with 'non-homologous isofunctional enzymes' (NISE) throughout*.

Reviewer 1

How did you deal with multifunctional/multidomain enzymes (such as RibF and such). I understand that they could be handled similarly but it might be worth mentioning in "methods"? Similar question, how do you deal with intrinsically multisubunit (heterooligimers) monofunctional enzymes? For example, how would you treat our newly discovered three-subunit L-LDH (former YkgEFG [64]) vs LldD? The complexity is when the actual roles of subunits (as well as cofactors) are not yet clear (I guess you would skip us for the lack of EC number anyways?).

***Authors' response: **In the revised articles, we added to the Methods a sentence on handling multifunctional (and multidomain) enzymes that have been assigned two or more EC numbers. As for multi-subunit enzymes, these were removed from the automatically processed set but typically re-examined in the course of manual analysis. The new L-lactate dehydrogenase *[64]*was missed because its EC 1.1.1.27 already had been listed among the NISE owing to the presence of the Rossmann-fold and the L-sulfolactate dehydrogenase-like fold proteins*.

Reviewer 1

You seem to be using quite a high hierarchical level (fold) to define "analogy". It is fine and safe. However, as far as I know popular folds (e.g. TIM) may be shared by proteins that are perceived to be evolutionary unrelated (non-homologous). This apparently reflects convergent evolution in the fold space (some folds simply emerge and stick with higher probability?). Is that true? If yes, then you may be underestmating a number of genuine analogous (in the evolutionary sense) pairs?

***Authors' response: **This is a very interesting and thorny point. Indeed, it is the case that distinct forms of the same fold, especially, in the case of versatile, abundant folds like the TIM barrel, are often considered non-homologous. From that perspective, by considering solely distinct folds, we might be underestimating the total number of non-homologous enzyme pairs. However, we are not sure that claims of convergent emergence of the same fold are valid. Of course, this is a fundamental issue in evolution of proteins that we would not attempt to solve in this paper which is dedicated to a different aspect of biochemical evolution. Moreover, even structures assigned to different folds might still be evolutionarily related (e.g. *[65,66]), *which would lead to an overestimation of the number of 'truly non-homologous' enzymes. All in all, we believe that requirement that alternative enzyme isoforms had distinct folds to be considered non-homologous provides a reasonable and straightforward approach to the search for NISE. A more permissive approach to the identification of alternative enzyme isoforms has been recently used by others *[14].

### Specific comments

Reviewer 1

"Likewise, two ubiquitin thiolesterases, ... actually possess distinct activities, cleaving polyubiquitin chains linked, respectively, to Lys-48 and Lys-63 residues [18]." I either miss something or disagree. If, indeed, the only difference is the position of the polyubiquitinilated lysine in substrate protein, they should be considered analogous. Same deal with any enzymes involved in PTMs or processing of biopolymers, kinases, proteases and so forth. There is no straightforward way to encode their "site-specificity", therefore, in my opinion, for this type of analysis even trypsin and chymotrypsin should be considered as one: "serine endopeptidase of the chymotrypsin family".

***Authors' response: **The original language was indeed imprecise. The Lys-48 and Lys-63 residues are amino acid of ubiquitin not of the ubiquitinated protein substrate. The sentence is corrected to reflect this fact in the revised manuscript. The respective polyubiquitin chains are distinct molecules, so the two thiolesterases, probably, should not be in the list of NISE*.

Reviewer 1

I notice that you have missed one of the FGGY kinases: RbtK - D-ribulokinase (EC 2.7.1.47). I disagree that "...Glycerol kinase ...catalyzes a reaction of lipid metabolism..." It is primarily catabolism of glycerol in bacteria. Just skip this statement or be more inclusive.

***Authors' response: **Corrected: we included EC 2.7.1.47 in the text (but not in the figure) and changed 'lipid metabolism' to 'glycerol metabolism'*.

Reviewer 1

The sentence "It appears that in each case, gluconate kinase was recruited from a family of kinases with activities toward closely related substrates." is not incorrect but I feel that it puts emphasis in a wrong place. The key is that recruitment happens from families with the same type of chemical reaction (e.g. phosphorylation). Similar or dissimilar substrate is (a) an ambiguous notion (is adenylsulfate similar to gluconate?) and (b) not that important (glycerol and gluconate are distinct enough). How about referring to classic "patchwork hypothesis" of Jensen in this discussion? We actually provided a penny to it in our Science paper [67].

***Authors' response: ****We agree, corrected*.

Reviewer 1

Discussion of patchy distribution is hard to appreciate without bringing up the issue of HGT. Have you examined patchy "analogous enzymes" (especially between Archaea and Bacteria) as a possible outcome of HGT?

***Authors' response:****We agree that the patchy distribution of analogous enzymes is most likely a consequence of rampant horizontal gene transfer *[43], *and this is explicitly mentioned in the text:"Non-homologous isoforms of enzymes seem to be recruited from pre-existing enzymes with related activities and specificities following duplication or horizontal transfer (apparently, the principal route of innovation in prokaryotes *[43,44], *where NISE are most common) of the respective genes." More specific and detailed analysis of the origins of NISE sets is of definite interest but beyond the scope of this paper*.

Reviewer 1

In the first paragraph of Discussion, you mention that you use "distinct folds (as) the ultimate proof of analogy", which is fine. However, you could mention that it might be another cause for underestimation of the extent of analogy among enzymes. Likewise, in addition to enzymes that "have not been biochemically characterized" there are also enzymes that were characterized but have not made it to EC nomenclature (or/and public databases like KEGG - just wonder whether you even thought of using SEED for metabolic enzymes, you could find a few interesting cases on top of what you have).

***Authors' response: **We have not used SEED in this work but hope to employ it in the next phase of this project. We have performed a literature search for potential cases of analogous enzymes but that search was not comprehensive*.

Reviewer 1

"...recruited from pre-existing enzymes of related specificities..." - same comment. Not wrong but wrong emphasis. Chemistry (type of reaction) is clearly more important for recruitment than "substrate specificity". In the extreme case of "retrograde concept" one would expect glucose isomerase to be recruited from hexokinase family, which is not the main route.

***Authors' response: **We agree, changed to 'activities and specificities'*.

Reviewer 1

"This has been recognized and successfully dealt with in the case of peptidases (EC 3.4.x.x. [50,51]) but remains a problem for various protein kinases and protein phosphatases..." I disagree with this view and interpretation (I already expressed it about proteases), but I won't argue. I am sure that plurality of protein kinases and phosphatases is driven by other factors (including "simplicity" of reaction and high "demand" in regulatory networks).

***Authors' response: **We believe that the disagreement here, if any, is semantic rather than substantial. From the purely operational point of view, we are interested whether there are multiple unrelated isoforms that are capable of acting on the same substrate and performing the same biochemical reaction. We agree with the reviewer's view on the driving factors behind the observed plurality of kinases and phosphatases*.

Reviewer 1

In Conclusions, it is important to choose words carefully to make the message clear. For example: "Sets of analogous, unrelated enzymes were detected for a substantial minority..." I would say at least "Sets of analogous, evolutionary unrelated enzymes (nonhomologous isoforms) were detected for a substantial fraction (up to 10%)".

***Authors' response: **Changed as suggested*.

Reviewer 1

"...unrelated mechanistic solutions can evolve". Although this claim is not incorrect, it cannot be directly deduced from the existence of "analogous enzymes". As an example, chymotrypsins and subtilisins are both serine proteases (eg they run the same mechanism) while having distinct folds and evolutionary origin. I mean this claim would require a separate analysis of mechanisms. The only solid claim is that the same chemical solutions (with the same or distinct mechanisms) can evolve independently (functional, but not necessarily mechanistic) convergence.

***Authors' response: **We agree, changed to 'evolutionarily unrelated solutions'*.

### Reviewer 2:  Keith F. Tipton, School of Biochemistry and Immunology, Trinity College, Dublin, Ireland (nominated by Martijn Huynen)

This a welcome update of the paper on analogous enzymes published by these authors in 1998. It contains much useful information and analysis. The supplementary Table S2, also available on-line, is particularly valuable. Some points that the authors should consider are listed below.

Reviewer 2

By concentrating on catalytic function in their discussions of evolutionary pressure, the authors may be missing the fact that an increasing number of enzymes are now recognized to be multifunctional (sometimes also called "moonlighting") proteins, with alternative, distinct, functions that may also be species-specific. Lists of several of these have been published (e.g., [68-70]). This indicates that the evolutionary pressures may be more complicated. The authors might consider referring to such complexities, perhaps in the context of their statement that "the existence of analogy shows that, at least, for numerous and diverse biochemical problems, unrelated mechanistic solutions can evolve".

***Authors' response: **Moonlighting is a very interesting phenomenon that is, however, only tangentially related to the issue of NISE (analogous enzymes). The very definition of "moonlighting proteins" as those that "have two different functions within a single polypeptide chain" *[68,71]*refers primarily to enzymes having additional non-enzymatic functions (e.g. transcriptional regulator, membrane receptor, growth factor, structural component, and so on). The above-cited reviews mention a single example of an enzyme with two entirely different enzymatic activities, the monomer of glyceraldehyde-3-phosphate dehydrogenase supposedly acting as uracil-DNA glycosylase *[72], *which still remains controversial *[73]. *In contrast, multifunctional enzymes *[74]*usually turn out to consist of two or more different domains. In all these examples of "multitasking", the evolutionary constraints are very different from those encountered by non-homologous enzymes that evolved to catalyze the same biochemical reaction*.

**Reviewer's comment to the authors' response: **The problem of 'moonlighting' is surely that the evolutionary pressures on the alternative, non-enzymic, function(s) may be different from those on the catalytic function and thus cannot be ignored when considering the pressure on the catalytic function. Of course, much of the literature assumes that the catalytic function is the main one, but in some cases this may be doubtful.

Reviewer 2

There are also cases of catalytic promiscuity where an enzyme catalyses distinct types of reaction (see e.g., [75]). If the reactions are sufficiently different, this should result in different EC numbers being assigned to the same protein. Furthermore, there are multifunctional proteins to catalysing different steps of an overall process, such as tryptophan synthase (EC 4.2.1.20) in some species. Thus, both 'one-to many' and 'many-to-one' relationships between EC numbers and proteins are possible. The former represents a problem, which the authors rightly point out, remains to be resolved for families such as the protein kinases, where a recognised enzyme, such as PKC-alpha may have several distinct substrates (see [76]) and one protein substrate may be phosphorylated by more than one kinase.

***Authors' response: **Catalytic promiscuity, when alternative chemical reactions take place in essentially the same active site, is an important factor in enzyme evolution *([75,77,78]*and references therein). As discussed above, we believe it to be a major source of NISE*.

Reviewer 2

As the authors recognise, the EC classification system is, or should be, solely based on the overall reaction catalysed. As such it is neither concerned with protein-sequence nor mechanistic differences and it is, perhaps, not surprising to find different proteins catalysing the same reaction. In this context, the suggestion "Nevertheless, it seems reasonable to consider expanding the EC system by officially recognizing the notion of a "class" within an EC node, such as, for example, superoxide dismutase (EC 1.15.1.1) class I, class II, and so on", might be clarified, since it would constitute a departure from the strict reaction-catalysed criterion and could risk detracting from its present utility. The authors should clarify what "classes" they propose should be included; would it be all analogous and homologous enzymes encompassed by each EC number? In some cases such material may be dealt with, more adequately, by complementary databases, which rely on the EC system. For enzymes that have different mechanism of action, the problem might best be resolved through systems such as the MaCiE (Mechanism, Annotation and Classification in Enzymes) database [79] or its offshoot Metal MACiE [80]. However, although MACiE does deal with the different mechanisms of the class I & II aldolases (EC 4.1.2.13), only the Cu/Zn superoxide dismutase is listed in these databases at present.

***Authors' response: **Adding the notion of a "class" to the EC system is only one of a number of possible ways to deal with NISE. Having supplementary specialized databases of enzyme mechanisms, such as MACiE *[79,80], *or sequence-based profiles, such as PRIAM *[13]*would be less intrusive but would force the users to rely on those outside sources for important information on the diversity of the enzymes in each EC node. This work identified NISE for almost 8% of all EC nodes, and many more EC nodes include divergent enzyme isoforms that still belong to the same superfamilies *[13,14]. *Given the scope of the problem, we felt that it should be brought to the attention of Prof. Tipton and other members of the Enzyme Commission*.

Reviewer 2

The authors refer to the "strict reliance on substrate specificity" being "a cause for certain confusion when the EC numbers are applied to mapping reactions on the metabolic map" and give the example of the enzymes that could catalyse the oxidation of D-glucose. It is not clear why they regard this as a problem. Surely it is beneficial to be able to find all the enzymes that may contribute to a metabolic process? As, for example, in the approach adopted by Reaction Explorer [81], and then to investigate the extents to which each does contribute to it, if at all?

***Authors' response: **Although we agree in principle, the decision on whether a certain pathway is operational in a certain organism often hinges on the presence or absence of a small group of pathway-specific enzymes *[82]. *In such cases, non-critical application of EC numbers may lead researchers to an erroneous assertion of the presence - or absence - of a given reaction (and hence the whole pathway) in the given genome*.

Reviewer 2

A problem, which the authors touch upon, is that of broad-specificity enzymes, such as alcohol dehydrogenase (EC 1.1.1.1) and monoamine oxidase (EC 1.4.3.4), where the reaction is described in general terms, with little no indication of all the substrates that may be involved. Such information, where known, can be found in the BRENDA database [83]. Similarly, the Enzyme List does not aim to give detailed species information, since that can also be found in the BRENDA database.

***Authors' response: **Although we agree, we have to note that this arrangement makes the BRENDA database the sole provider of this critically important information. In our opinion, the EC system might benefit from inclusion of this type of data*.

**Reviewer's comment to the authors' response: **BRENDA is not the only source of specificity data and I did not intend to imply that it was. KEGG also gives such information. We collaborate closely with both databases, and take the view that if they are doing a good job, why should we want to duplicate them?

I am still not clear what you may have in mind by 'adding a class'. We have received many suggestions in the past for additional EC digit to cover several diverse areas, including mechanism, medically-relevant enzymes, enzymes from different species, isoenzymes etc. So far we have decided that this would not be helpful. The alternative might be adding a 'NISE' field to each entry but, as mentioned above, a direct link to the corresponding PRIAM page might be more helpful.

### Reviewer 3: Igor B. Zhulin, University of Tennessee - Oak Ridge National Lab., Oak Ridge, Tennessee, USA

This paper extends the authors' previous work identifying analogous enzymes more than a decade ago. The authors expanded their search methods by utilizing both the Swiss Prot database and the KEGG database to better associate proteins with enzymatic activity. By the author's own admission, no strong trends were observed in the dataset, but they were able to identify a few very interesting patterns, including enrichment of analogous enzymes among glycoside hydrolases, enzymes involved in oxidative stress relief, and among the TIM Barrel and NAP(P)-binding Rossmann structural folds. As expected, the authors find that the number analogous enzymes scales with increasing genome size. The authors discuss the evolutionary origins of some of the trends noted above, as well as the limitations imposed on their identification schemes by the EC numbering system itself. Overall, I do like this paper a lot, especially because in my lab we have recently become interested in one particular family of analogous enzymes. So, I enjoyed looking at a bigger picture, while picturing our own work in its context.

The analysis scheme employed is straightforward and utilizes proven bioinformatic methodology. The authors appear to utilize conservative criteria for inclusion of data for the analysis, so the results are likely to under-predict rather than over-predict analogous enzymes. The results greatly expand the listing of analogous enzymes and the extensive supplementary material provides useful information for specialist interested in any particular family of enzymes.

The inclusion of numerous genomes through the use of the KEGG database allowed analysis of analogous enzymes to be conducted on a sufficient scale to give a fairly good approximation of the their relative abundance and the importance of analogous inventions during evolution. The coverage of structural information, sequence, and biological information seems to be such that the boundaries for the proportion of analogous enzymes (~10% of the EC nodes) seem unlikely to significantly change with future genome sequencing.

Lack of true novelty in this analysis is a minor quibble, as it generated a useful resource in and of itself and the specific cases highlighted are of interest in a number of fields. The use of EC number annotations may be suspect in some cases where the traditional sequence similarity based annotation methods are unreliable or where the EC definitions are inadequate. I can offer a couple of examples, where we happened to dig around a little bit. For instance, Table S1 lists a couple of cellulases (entries #78 and #91) in glycoside hydrolase families 10 and 11. It appears that there are no experimentally defined cellulases in these families, and enzymes shown are putative xylanases. It also might be just a matter of semantics, since these enzyme are likely to be hemicellulases (technically could be called cellulases, I guess). Anyway, the authors are fully aware of the limitations imposed and there is no way to verify available experimental evidence for each and every entry in such a large-scale effort. The vast majority of the enzymes included in the study are readily identified by sequence similarity based annotations, so the conclusions as a whole are sound.

***Authors' response: **We fully agree with these comments*.

## Supplementary Material

Additional file 1 **Supplementary Table S1**. An update to the 1998 listing of analogous enzymes. Predicted analogous enzymes pairs from the 1998 list that have been removed from the new list are highlighted in yellow. The EC numbers are hyperlinked with the ENZYME database entries, examples are linked to UniProt, structures - to PDB, folds - to SCOP, families - to Pfam, and references - to PubMed. Click here for file

Additional file 2 **Supplementary Table S2**. A new listing of analogous enzymes. The EC numbers are hyperlinked with the ENZYME database entries, protein entries are linked to the NCBI protein database and UniProt, PDB entries - to PDB, SCOP folds - to SCOP, protein superfamilies - to SUPERFAMILY, protein families - to Pfam, and references - to PubMed. Click here for file

Additional file 3 Supplementary Tables S3-S9 Click here for file
