# Supplementary material for: Non-homologous isofunctional enzymes: A systematic analysis of alternative solutions in enzyme evolution
Source: Biol Direct. 2010 Apr 30;5:31. doi: 10.1186/1745-6150-5-31 (PMC2876114; doi:10.1186/1745-6150-5-31)
Supplement: Additional file 1 — Supplementary Table S1. An update to the 1998 listing of analogous enzymes. Predicted analogous enzymes pairs from the 1998 list that have been removed from the new list are highlighted in yellow. The EC numbers are hyperlinked with the ENZYME database entries, examples are linked to UniProt, structures - to PDB, folds - to SCOP, families - to Pfam, and references - to PubMed. [file 1745-6150-5-31-S1.PDF]

Table S1. An update to the 1998 listing of analogous enzymes

This listing was originally prepared as a web supplement to the article by M.Y. Galperin, D.R. Walker and E.V. Koonin **"Analogous enzymes: Independent inventions in enzyme evolution"**, published in 1998 in *Genome Research* **8:** 779-790. Free full text.

This updated version lists the same 105 EC nodes as the original one and is Supplementary Table S1 to the manuscript by M.V. Omelchenko, M.Y. Galperin, Y.I. Wolf and E.V. Koonin **"Analogous enzymes: A systematic analysis of alternative solutions in enzyme evolution**, submitted for publication in January 2010.

No.	EC no.	Enzyme name	Example	PDB	SCOP fold	Family (Pfam)	Ref.	Comments
1	**1.1.1.1**	Alcohol dehydrogenase (NAD+)	ADH1B_HUMAN	**1HDY**	GroES-like & Rossmann-fold	Zinc-contaning alcohol dehydrogenase	**9802210, 8201622**	Three distinct structural folds
	**1.1.1.1**	Alcohol dehydrogenase (NAD+)	ADH4_YEAST	**2BI4**	Dehydroquinate synthase-like	Iron-contaning alcohol dehydrogenase	**3282541, 2823079**	
	**1.1.1.1**	Alcohol dehydrogenase (NAD+)	ADH_DROME	**1MG5**	Rossmann-fold	Short chain dehydrogenase	**15016358, 11669613**	
2	**1.1.1.2**	Alcohol dehydrogenase (NADP+)	ADHC_MYCBO	**1YQD**	GroES-like & Rossmann-fold	Zinc-contaning alcohol dehydrogenase	**1427101, 15829607**	Three distinct structural folds
	**1.1.1.2**	Alcohol dehydrogenase (NADP+)	AK1A1_HUMAN	**2ALR**	TIM beta/alpha-barrel	Aldo/keto reductase	**15299353, 10486210**	
	**1.1.1.2**	Alcohol dehydrogenase (NADP+)	ADH4_SCHPO	**1O2D**	Dehydroquinate synthase-like	Iron-contaning alcohol dehydrogenase	**15327949, 14705036**	
3	**1.1.1.27**	L-lactate dehydrogenase	LDHA_HUMAN	**1EZ4**	Rossmann-fold & LDH C-terminal domain	Lactate & malate dehydrogenases	**PubMed**	Two distinct structural folds
	**1.1.1.27**	L-lactate dehydrogenase	LDH_RALEH	**1VBI**	L-sulfolactate dehydrogenase-like	Malate/L-Lactate dehydrogenases	**8405966**	
4	**1.1.1.28**	D-lactate dehydrogenase	LDHD_ECOLI	**2DLD**	Rossmann-fold & Flavodoxin-like	Formate/glycerate dehydrogenases	**9025293**	Two distinct structural folds

	**1.1.1.28**	D-lactate dehydrogenase	DLD_ECOLI	**1F0X**	FAD-binding domain & Ferredoxin-like	D-lactate dehydrogenase	**10944213**	
5	**1.1.1.37**	Malate dehydrogenase	MDH_HALMA	**2J5K**	Rossmann-fold & LDH C-terminal domain	L-lactate/malate dehydrogenases	**8476859, 10653643**	Two distinct structural folds
	**1.1.1.37**	Malate dehydrogenase	MDH_METFE	**2G8Y**	L-sulfolactate dehydrogenase-like	Malate/L-Lactate dehydrogenases	**2110059, 10850983**	
6	**1.1.1.42**	Isocitrate dehydrogenase (NADP+)	IDH_ECOLI	**1IDC**	Isocitrate/ Isopropylmalate dehydrogenase-like	Isocitrate/ isopropylmalate dehydrogenase	**1888729**	Same fold, same superfamily
	**1.1.1.42**	Isocitrate dehydrogenase (NADP+)	IDH_AZOVI	**1ITW**	Isocitrate/ Isopropylmalate dehydrogenase-like	Monomeric isocitrate dehydrogenase	**4149369**	
7	**1.1.1.47**	Glucose 1-dehydrogenase	DHG_BACSU	**1GCO**	Rossmann-fold	Short chain dehydrogenase	**3082854**	Two distinct structural folds
	**1.1.1.47**	Glucose 1-dehydrogenase	DHG_THEAC	**1HDZ**	GroES-like	Alcohol dehydrogenase GroES-like domain	**2803257, 8436115**	
8	**1.1.1.50**	3alpha-hydroxysteroid dehydrogenase	Q9ZFY9_COMTE	**1FJH**	Rossmann-fold	Short chain dehydrogenase	**10833462**	Two distinct structural folds
	**1.1.1.50**	3alpha-hydroxysteroid dehydrogenase	AK1C4_HUMAN	**2FVL**	TIM beta/alpha barrel	Aldo/keto reductase	**1530633, 11158055**	
9	**1.1.1.62**	Estradiol 17ß-dehydrogenase	DHB1_HUMAN	**1FDS**	Rossmann-fold	Short chain dehydrogenase	**2846351, 7663947**	Two distinct structural folds
	**1.1.1.62**	Estradiol 17ß-dehydrogenase	DHB5_MOUSE	**1Q5M**	TIM beta/alpha barrel	Aldo/keto reductase	**PubMed**	

10	**1.2.1.2**	Formate dehydrogenase	FDHF_ECOLI	**1FDI**	Formate dehydrogenase/ DMSO reductase	Molybdopterin-containing oxidoreductases	**9036855**	Two distinct structural folds
	**1.2.1.2**	Formate dehydrogenase	FDH_PSESR	**2NAC**	Rossmann-fold & Flavodoxin-like	2-hydroxyacid dehydrogenases	**1597184, 8484798**	
11	**1.2.99.2**	Carbon-monoxide dehydrogenase	DCML_OLICO	**1N60**	CO dehydrogenase ISP C-domain like and others		**10482497, 12475995**	Two distinct structural folds
	**1.2.99.2**	Carbon-monoxide dehydrogenase	COOS_RHORU	**1JQK**	Prismane protein-like	Prismane/CO dehydrogenase family	**8561463, 11593006**	
12	**1.3.1.33**	Protochlorophyllide reductase	BCHN_RHOCA	**3MIN**	Nitrogenase Fe-Mo fold	Protochlorophyllide reductase	**10811655**	The two forms use different electron donors
	**1.3.1.33**	Protochlorophyllide reductase	PORB_ARATH	*1hdu*	Rossmann-fold	Short chain dehydrogenase	**7659751**	
13	**1.5.1.3**	Dihydrofolate reductase	DYR_BACSU	**1MVS**	Dihydrofolate reductase-like	Dihydrofolate reductases	**1731871**	Two distinct structural folds
	**1.5.1.3**	Dihydrofolate reductase	FOLM_ECOLI	*3edm*	Rossmann-fold	Short chain dehydrogenase	**14617668**	
14	**1.6.99.3**	NADH dehydrogenase	DHNA_BACYN	**1XHC**	FAD/NAD(P)-binding domain	NADH dehydrogenase	**1917890**	Two distinct structural folds
	**1.6.99.3**	NADH dehydrogenase	NDUS1_HUMAN	**2FUG**	Formate dehydrogenase/ DMSO reductase	Complex I 75 kDa subunit family	**1935949**	
15	**1.10.3.1**	Catechol oxidase	DXA2_DROME	N/A	Corrected to PSMD3_DROME, 26S proteasome regulatory subunit 3		**1909680**	Misassignment corrected
	**1.10.3.1**	Diphenol oxidase	PPO_SPIOL	**1BT1**	Di-copper centre-containing domain		**7794929**	

16	**1.11.1.5**	Cytochrome-c peroxidase	CCPR_PSEAE	**1EB7**	Cytochrome c	Di-heme cytochrome c peroxidase	**7781769, 1657179, 8591033**	Two distinct structural folds
	**1.11.1.5**	Cytochrome-c peroxidase	CCPR_YEAST	**1EBE**	Heme-dependent peroxidases	Peroxidase	**2169873, 6092361**	
17	**1.11.1.6**	Catalase	CATA_HUMAN	**1QQW**	Heme-dependent catalase	Heme-dependent catalase	**10666617, 3755525**	Three distinct structural folds
	**1.11.1.6**	Catalase	CATA_ECOLI	**2FXG**	Heme-dependent peroxidases	Peroxidase	**374409, 2670897, 8508796**	
	**1.11.1.6**	Catalase	MCAT_LACPL	**1JKU**	Ferritin-like	Manganese catalase	**8939876**	
18	**1.11.1.7**	Peroxidase	PRDX6_MOUSE	**1PRX**	Thioredoxin	Thioredoxin	**9291135, 10395907, 9587003**	Three distinct structural folds
	**1.11.1.7**	Peroxidase	PERM_HUMAN	**1MYP**	Heme-dependent peroxidases	Animal heme peroxidase	**2154223**	
	**1.11.1.7**	Peroxidase	YCDB_ECOLI	*2D3Q*	Ferredoxin-like	Dyp-type peroxidase-like	**16551627, 14684913**	
19	**1.11.1.10**	Chloride peroxidase	PRXC_PSEPY	**1A88**	Alpha/beta-hydrolases	Haloperoxidase	**8344520**	Three distinct structural folds
	**1.11.1.10**	Chloride peroxidase	PRXC_CURIN	**1VNC**	Acid phosphatase	Type 2 phosphatidic acid phosphatase (PAP2)	**9165086**	
	**1.11.1.10**	Chloride peroxidase	PRXC_CALFU	**2CPO**	EF Hand-like	Cloroperoxidase	**8747463, 11278701**	
20	**1.14.15.3**	Alkane 1-monooxygenase	ALKB_PSEOL	*1AFR*	*Ferritin-like (predicted)*	Fatty acid desaturase	**2647718**	Two distinct structural folds
	**1.14.15.3**	Alkane 1-monooxygenase	CP4AM_HUMAN	**1TQN**	Cytochrome P450	Cytochrome P450	**PubMed**	

21	**1.15.1.1**	Superoxide dismutase	SODF_ECOLI	**1ISA**	Fe,Mn superoxide dismutase	Fe,Mn superoxide dismutase	**2447093**	Three distinct structural folds
	**1.15.1.1**	Superoxide dismutase	SODC_ECOLI	**1ESO**	Immunoglobulin-like beta-sandwich	Cu,Zn superoxide dismutase	**9405149**	
	**1.15.1.1**	Superoxide dismutase	SODN_STRSO	**1Q0D**	Four-helical up-and-down bundle	Nickel-containing superoxide dismutase	**8836134, 15173586**	
22	**1.17.4.2**	Ribonucleoside-triphosphate reductase	NRDD_ECOLI	**1H7A**	PFL-like glycyl radical enzymes	Anaerobic ribonucleotide reductase	**7852304, 12655046**	Same fold, same superfamily
	**1.17.4.2**	Ribonucleoside-triphosphate reductase	RTPR_LACLE	**1L1L**	PFL-like glycyl radical enzymes	B12-dependent (class II) ribonucleotide reductase	**11875520**	
23	**1.18.99.1**	Hydrogenase (now **EC 1.12.2.1**)	PHNL_DESGI	**1FRV**	HydA/Nqo6-like & HydB/Nqo4-like	Ni-Fe hydrogenase	**2651421, 7854413**	Two distinct structural folds
	**1.18.99.1**	Hydrogenase (now **EC 1.12.7.2**)	PHF1_CLOPA	**1FEH**	Fe-only hydrogenase	Iron-only hydrogenase	**1911757, 9836629**	
24	**2.1.1.17**	Phosphatidylethanolamine N-methyltransferase	PMTA_RHOSH	*3DH0*	SAM-dependent methyltransferases	SAM-dependent methyltransferase	**8340421**	Two distinct structural folds
	**2.1.1.17**	Phosphatidylethanolamine N-methyltransferase	PEM1_YEAST	N/A	Integral membrane protein		**2445736**	
25	**2.3.1.15**	Glycerol-3-phosphate O-acyltransferase	PLSB_ECOLI	N/A		Acyltransferase	**6350296**	Same catalytic domain
	**2.3.1.15**	Glycerol-3-phosphate O-acyltransferase	PLSB_CUCMO	**1K30**	Glycerol-3-phosphate 1-acyltransferase	Glycerol-3-phosphate 1-acyltransferase	**11377195**	
26	**2.3.1.28**	Chloramphenicol O-acetyltransferase	CAT4_AGRTU	**1XAT**	Single-stranded left-handed beta-helix	Galactoside acetyltransferase	**2013403, 9578552**	Two distinct structural folds
	**2.3.1.28**	Chloramphenicol O-acetyltransferase	CAT3_ECOLX	**3CLA**	CoA-dependent acyltransferases	Chloramphenicol acetyltransferase	**2187098**	

27	**2.3.1.43**	Phosphatidylcholine-sterol O-acyltransferase	LCAT_HUMAN	*1ex9*	*Alpha/beta hydrolase fold (predicted)*		**2823898**	Two distinct structural folds
	**2.3.1.43**	Phosphatidylcholine-sterol O-acyltransferase	GCAT_AERHY	*1vig*	*Eukaryotic type KH-domain (predicted)*		**3280033**	
28	**2.3.2.13**	Protein-glutamine gamma-glutamyltransferase	F13A_HUMAN TGAS_STRMB	**1FIE 1IU4**	Cysteine proteinases	Transglutaminase, microbial transglutaminase	**9839945, 2901091, 2877456**	Two distinct structural folds
	**2.3.2.13**	Protein-glutamine gamma-glutamyltransferase	TGL_BACSU	*1ssq*	*Single-stranded left-handed beta-helix (predicted)*		**9692191**	
29	**2.4.1.25**	4-a-glucanotransferase	MALQ_ECOLI	**1FP8**	TIM beta/alpha-barrel	Glycosyl hydrolase family 77	**2845225**	Two distinct structural folds
	**2.4.1.25**	4-a-glucanotransferase	MALQ_THELI	**1K1X**	7-stranded beta/alpha barrel	Glycosyl hydrolase family 57	**12618437**	
30	**2.5.1.18**	Glutathione S-transferase	GST_ECOLI GSTK1_HUMAN	**1A0F 1R4W**	Thioredoxin & GST C-terminal domain	Glutathione S-transferase, class beta, class kappa	**2185038, 16081649, 14717589**	Three distinct structural folds
	**2.5.1.18**	Glutathione S-transferase	MGST1_HUMAN	**2H8A**	MAPEG domain	MAPEG family	**8812420, 9278457, 10091672, 16806268**	
	**2.5.1.18**	Glutathione S-transferase	FOSA_SERMA	**1NPB**	Glyoxalase/ Bleomycin resistance protein	Antibiotic resistance proteins	**15075406, 9115979**	
31	**2.6.1.44**	Alanine-glyoxylate aminotransferase	SPYA_HUMAN	**1H0C**	PLP-dependent transferases	Cystathionine synthase-like	**2253628, 12899834**	Same fold, same superfamily
	**2.6.1.44**	Alanine-glyoxylate aminotransferase	AGT2_RAT	**1Z3Z**	PLP-dependent transferases	GABA-aminotransferase	**7592550**	

32	**2.7.1.2**	Glucose kinase	GLK_ECOLI	**1SZ2**	Ribonuclease H-like motif	Glucokinase	**9023215**	Same fold, same superfamily
	**2.7.1.2**	Glucose kinase	GLK_STRCO	**1WOK**	Ribonuclease H-like motif	ROK family	**1435260**	
	**2.7.1.2**	Glucose kinase	HXK1_HUMAN	**1DGK**	Ribonuclease H-like motif	Hexokinase	**1637300, 9735292**	
33	**2.7.1.4**	Fructokinase	SCRK_ECOLX	*1TZ6*	Ribokinase-like	PfkB family carbohydrate kinase	**8278523**	Two distinct structural folds
	**2.7.1.4**	Fructokinase	SCRK_STRMU	**1XC3**	Ribonuclease H-like motif	Actin-like ATPase domain	**8336109**	
34	**2.7.1.11**	6-Phosphofructokinase	K6PF1_ECOLI	**1PFK**	Phosphofructokinase	Phosphofructokinase	**2975709**	Two distinct structural folds
	**2.7.1.11**	6-Phosphofructokinase	K6PF2_ECOLI	**3CQD**	Ribokinase-like	PfkB family carbohydrate kinase	**18762190, 6310120**	
35	**2.7.1.12**	Gluconokinase	GNTK_ECOLI	**1KNQ**	P-loop NTPases	Gluconate kinase	**8655507, 11468405**	Two distinct structural folds
	**2.7.1.12**	Gluconokinase	GNTK_BACSU	**2ITM**	Ribonuclease H-like motif	Glycerol kinase	**3020045**	
36	**2.7.1.107**	Diacylglycerol kinase	KDGL_ECOLI	N/A	Integral membrane protein	Prokaryotic diacylglycerol kinase	**2984194, 8071224, 18611377**	Two distinct structural folds
	**2.7.1.107**	Diacylglycerol kinase	DGKG_HUMAN	*2qv7*	NAD kinase (predicted)	Diacylglycerol kinase catalytic domain	**8034597**	
37	**2.7.4.3**	Adenylate kinase	KAD_ECOLI	**1AKE**	P-loop NTPases	Adenylate kinase	**9715904**	Same structural family
	**2.7.4.3**	Adenylate kinase	KADA_METVO	**1NKS**	P-loop NTPases	Archaeal adenylate kinase	**7768791, 9055821, 9733648**	

38	**2.7.7.2**	FAD synthetase	FAD1_YEAST	**3G5A**	Adenine nucleotide alpha hydrolase (predicted)	PAPS reductase	**7799934, 19375431**	Same fold, different superfamilies, discussed in Huerta et al.
	**2.7.7.2**	FAD synthetase	RIBF_CORAM	**1S4M**	Adenine nucleotide alpha hydrolase	FAD synthetase	**3023344, 9454067**	
39	**2.7.7.4**	Sulfate adenylyltransferase	CYSN_ECOLI CYSD_ECOLI	**1ZUN**	P-loop NTPases and Adenine nucleotide alpha hydrolase		**2828368**	CysD and MET3 belong to the same superfamily
	**2.7.7.4**	Sulfate adenylyltransferase	MET3_YEAST	**1JEE**	Adenine nucleotide alpha hydrolase	ATP-sulfurylase	**11157739**	
40	**2.7.7.9**	UTP-glucose-1-phosphate uridylyltransferase	GALU_ECOLI	**2E3D**	Nucleotide-diphospho-sugar transferases	Nucleotidyl transferase	**7961613, 17322528**	Two distinct structural folds
	**2.7.7.9**	UTP-glucose-1-phosphate uridylyltransferase	UGPA_HUMAN	*2icy*	Single-stranded left-handed beta-helix	UTP-glucose-1-phosphate uridylyltransferase	**8354390, 17178129**	
41	**2.7.7.12**	Galactose-1-phosphate uridylyltransferase	GAL7_ECOLI	**1GUP**	HIT-like	Galactose-1-phosphate uridylyltransferase 1	**321007, 7669762**	Same fold, same superfamily
	**2.7.7.12**	Galactose-1-phosphate uridylyltransferase	GALT_LACHE	*1gup*	HIT-like (predicted)	Galactose-1-phosphate uridylyltransferase 2	**2066342**	
42	**2.7.7.19**	Polynucleotide adenylyltransferase	PCNB_ECOLI	**1MIY**	Nucleotidyltransferase & Poly A polymerase C-terminal region-like	Poly A polymerase C-terminal region-like	**10361280**	Same fold, same superfamily (catalytic domain)
	**2.7.7.19**	Polynucleotide adenylyltransferase	PAP_HUMAN	**1Q78**	Nucleotidyltransferase & PAP/OAS1 substrate-binding domain & Ferredoxin-like	Poly(A) polymerase, PAP, middle domain&	**9061026**	

43	**2.7.7.25**	tRNA adenylyltransferase	CCA_ECOLI	**1VFG**	Nucleotidyltransferase & Poly A polymerase C-terminal region-like	Bacterial CCA-adding enzyme type 1	**2204621**	Same fold, same superfamily (catalytic domain)
	**2.7.7.25**	tRNA adenylyltransferase	CCA_SULSH	**1UET**	Nucleotidyltransferase & PAP/OAS1 substrate-binding domain & Ferredoxin-like	Archaeal tRNA CCA-adding enzyme substrate-binding domain	**8809016, 15590678**	
44	**2.7.8.8**	CDP-diacylglycerol--serine O-phosphatidyltransferase	PSS_ECOLI	*1BYR*	Phospholipase D/ nuclease	Phospholipase D Active site motif	**1323044, 10074947**	Two distinct structural folds
	**2.7.8.8**	CDP-diacylglycerol--serine O-phosphatidyltransferase	PSS_YEAST	N/A	Integral membrane protein	CDP-alcohol phosphatidyltransferase	**3040403**	
45	**3.1.2.15**	Ubiquitin thiolesterase	UBP5_HUMAN	**2G43**	Cysteine proteinases	Ubiquitin carboxyl-terminal hydrolase	**7498549**	Different activities: cleave polyubiquitin chains linked, respectively, to Lys-48 and Lys-63
	**3.1.2.15**	Ubiquitin thiolesterase	STALP_HUMAN	**2ZNR**	Cytidine deaminase-like (predicted)	JAB1/MPN/Mov34 metalloenzyme	**15314065, 18758443**	
46	**3.1.3.48**	Protein-tyrosine-phosphatase	PTPA_STRCO	**1D1P**	Phosphotyrosine protein phosphatases I-like	Low-molecular-weight phosphotyrosine protein phosphatases	**8550407, 1304913**	Five distinct structural folds
	**3.1.3.48**	Protein-tyrosine-phosphatase	PTPRD_HUMAN	**1LAR**	Phosphotyrosine protein phosphatases II	Higher-molecular-weight phosphotyrosine protein phosphatases	**2170109, 8833149**	
	**3.1.3.48**	Protein-tyrosine-phosphatase	MPIP3_HUMAN	**1QB0**	Rhodanese/Cell cycle control phosphatase	Cell cycle control phosphatase	**8276463**	
	**3.1.3.48**	Protein-tyrosine-phosphatase	YWQE_BACSU	*2ANU*	*7-stranded beta/alpha barrel*	PHP domain	**15866923**	

	**3.1.3.48**	Protein-tyrosine-phosphatase	EYA3_MOUSE	*1JUD*	*HAD-like*	HAD-like hydrolase	**14628042, 14628052**	
47	**3.2.1.1**	Alpha-amylase	AMY1_ECOLI	**1EA9**	TIM beta/alpha-barrel & Glycosyl hydrolase	Alpha-amylase	**9268356**	Two distinct structural folds
	**3.2.1.1**	Alpha-amylase	AMYA_PYRFU	**1K1X**	7-stranded beta/alpha barrel	Glycosyl hydrolase family 57	**8226990**	
48	**3.2.1.4**	Cellulase	GUNA_PSEFL	**1UT9**	Alpha/alpha toroid	Glycosyl hydrolase family 9 (cellulase E)	**2851699**	At least four distinct structural folds
	**3.2.1.4**	Cellulase	GUNA_CLOCE	**1EDG**	TIM beta/alpha-barrel	Beta-glycanases	**1744052, 8535787**	
	**3.2.1.4**	Cellulase	GUN1_STRHA	**2BOD**	7-stranded beta/alpha barrel	Glycosyl hydrolase family 6	**1400190**	
	**3.2.1.4**	Cellulase	GUNM_CLOTM	**2FVG**	Phosphorylase/ hydrolase	Cellulase M	**GenBank**	
	**3.2.1.4**	Cellulase	GUN_ASPAC	**1KS4**	Concanavalin A-like lectins/glucanases	Glycosyl hydrolase family 11 (cellulase G)	**2249253, 7586029, 2379837**	
	**3.2.1.4**	Cellulase	GUNE_RUMFL	*1L0H*	Acyl carrier protein-like (predicted)		**8360615**	
	**3.2.1.4**	Cellulase	GUNA_PAELA	*2EQD*	TIM beta/alpha-barrel (predicted)		**17905739**	
49	**3.2.1.8**	Endo-1,4-ß-xylanase	XYNA_BUTFI	**1XAS**	TIM beta/alpha-barrel	beta-glycanases	**2198249, 1909424**	Two distinct structural folds
	**3.2.1.8**	Endo-1,4-ß-xylanase	XYNA2_CLOSR	**1QH6**	Concanavalin A-like lectins/ glucanases	Glycosyl hydrolase family 11 (cellulase G)	**7763496**	
50	**3.2.1.11**	Dextranase	DEXT_STRDO	N/A			**8021165**	Two distinct structural folds

	**3.2.1.11**	Dextranase	DEXT_PENMI	**1OGM**	Single-stranded right-handed beta-helix	Glycosyl hydrolase family 28	**8905923, 12962629**	
51	**3.2.1.14**	Chitinase	CHIA_SERMA	**1RD6**	TIM beta/alpha barrel	Type II chitinase	**PubMed**	Two distinct structural folds
	**3.2.1.14**	Chitinase	CHI1_ORYSA	**2BAA**	Lysozyme-like	Family 19 glycosidase	**1893114**	
52	**3.2.1.17**	Lysozyme	LYS_CLOAB	**1JFX**	TIM beta/alpha barrel	1,4-beta-N-acetylmuraminidase	**1599233, 7649184**	Two distinct structural folds
	**3.2.1.17**	Lysozyme	LYS_BPP1	**1XJT**	Lysozyme-like	Phage lysozyme	**8576044, 9514719**	
	**3.2.1.17**	Lysozyme	ACMA_LACLM	**2ZYC**	*Lysozyme-like*	Glycosyl hydrolase family 73	**7883712, 10049388, 19351587**	
	**3.2.1.17**	Lysozyme	LYB_BACSU	N/A	N/A		**3148618**	
53	**3.2.1.20**	Alpha-glucosidase	MALT_CANAL	**1JI1**	TIM beta/alpha barrel & Glycosyl-hydrolase domain	Glycosyl hydrolase family 13	**1400249**	Two distinct structural folds
	**3.2.1.20**	Alpha-glucosidase	AGLA_THEMA	**1OBB**	Rossmann-fold & LDH C-terminal domain	Glycosyl hydrolase family 4	**12062450, 10972187**	
54	**3.2.1.21**	Beta-glucosidase	BGLA_THEMA	**1OIF**	TIM beta/alpha barrel	Glycosyl hydrolase family 1	**8277941, 14624580**	Same fold, same superfamily
	**3.2.1.21**	Beta-glucosidase	BGLS_BUTFI	**1X38**	TIM beta/alpha barrel	Glycosyl hydrolase family 3	**2262790**	
55	**3.2.1.22**	Alpha-galactosidase	AGAL_STRMU	**1ZY9**	TIM beta/alpha barrel	Glycosyl hydrolase family 36	**1649890**	Two distinct structural folds
	**3.2.1.22**	Alpha-galactosidase	AGAL_ECOLI	**1OBB**	Rossmann-fold & LDH C-terminal_domain	Glycosyl hydrolase family 4	**2831880**	

56	**3.2.1.23**	Beta-galactosidase	BGAL_ECOLI	**1BGL**	TIM beta/alpha-barrel	Glycosyl hydrolase family 2	**11732897**	Same fold, same superfamily
	**3.2.1.23**	Beta-galactosidase	BGAL_SULAC		TIM beta/alpha-barrel	Glycosyl hydrolase family 1	**2508066**	
57	**3.2.1.28**	Alpha,alpha-trehalase	TREA_ECOLI	**2JG0**	Alpha/alpha toroid	Glycosyl hydrolase family 37	**2820965, 17455176**	Same fold, same superfamily
	**3.2.1.28**	Alpha,alpha-trehalase	TREA_EMENI	*1H54*	*Alpha/alpha toroid (predicted)*	Glycosyl hydrolase family 65	**9140977**	
58	**3.2.1.35**	Hyaluronidase	HYAL2_HUMAN	**1FCU**	TIM beta/alpha barrel	Glycosyl hydrolase family 56	**9712871, 17503783**	Two distinct structural folds
	**3.2.1.35**	Hyaluronidase	HYLP2_BPH45	**2PK1**	Triple-stranded beta-helix	Hyaluronidase_1	**7622224**	
	**3.2.1.35**	Hyaluronidase	HEMO_PIG	**1HXN**	4-bladed beta-propeller	Hemopexin domain	**7798203, 8590016**	Most likely a mistake
59	**3.2.1.37**	Xylan 1,4-ß-xylosidase	XYNB_BACPU	**1YIF**	5-bladed beta-propeller	Glycosyl hydrolase family 43	**2440680, 7766665**	Three distinct structural folds
	**3.2.1.37**	Xylan 1,4-ß-xylosidase	XYNB_THESA	**1PX8**	TIM beta/alpha barrel	Glycosyl hydrolase family 39	**8612648**	
	**3.2.1.37**	Xylan 1,4-ß-xylosidase	XYLA1_BACST	N/A	N/A	Glycosyl hydrolase family 52	**8074507, 11322943**	
60	**3.2.1.39**	Glucan endo-1,3-ß-D-glucosidase	E13A_ARATH	**2CYG**	TIM beta/alpha barrel	Glycosyl hydrolase family 17	**11405630, 16421930**	Three distinct structural folds
	**3.2.1.39**	Glucan endo-1,3-ß-D-glucosidase	E13B_BACCI	*1UPS*	Concanavalin A-like lectins/ glucanases	Glycosyl hydrolase family 16	**2311931**	
	**3.2.1.39**	Glucan endo-1,3-ß-D-glucosidase	E13B_CELCE	N/A	N/A	Glycosyl hydrolase family 64	**1985933**	

61	**3.2.1.55**	Alpha-N-arabinofuranosidase	ABFA_STRLI	**2C7F**	TIM beta/alpha barrel	Glycosyl hydrolase family 51	**8092996, 16336192**	Three distinct structural folds
	**3.2.1.55**	Alpha-N-arabinofuranosidase	XYLB_BUTFI	**1YRZ**	Concanavalin A-like lectins/glucanases & 5-bladed beta-propeller	Glycosyl hydrolase family 43	**1905520, 18980579**	
	**3.2.1.55**	Alpha-N-arabinofuranosidase	XYNC_PSEFL	N/A	*5-bladed beta-propeller (predicted)*	Glycosyl hydrolase family 62	**2125205**	
62	**3.2.1.58**	Glucan 1,3-ß-glucosidase	EXG_CANAL	**2PB1**	TIM beta/alpha barrel	Glycosyl hydrolase family 5 (cellulase A)	**8436950, 10610795**	Two distinct structural folds
	**3.2.1.58**	Glucan 1,3-ß-glucosidase	EXG1_COCCA	**3EQO**	*Single-stranded right-handed beta-helix*	Glycosyl hydrolase family 55	**8135518**	
63	**3.2.1.73**	Licheninase	GUB_NICPL	**2CYG**	TIM beta/alpha barrel	Glycosyl hydrolase family 17	**PubMed**	Three distinct structural folds
	**3.2.1.73**	Licheninase	GUB_BACSU	**1GBG**	Concanavalin A-like lectins/glucanases	Glycosyl hydrolase family 16	**1740123**	
	**3.2.1.73**	Licheninase	GUB_BACCI	**1V5C**	Alpha/alpha toroid	Glycosyl hydrolase family 8 (cellulase D)	**2377467**	
64	**3.2.1.78**	Mannan endo-1,4-ß-mannosidase	MANA_PSEFL	**1GW1**	TIM beta/alpha barrel	Glycosyl hydrolase family 26	**7848261, 12203498**	Same fold, same superfamily
	**3.2.1.78**	Mannan endo-1,4-ß-mannosidase	MANB_CALSA	**1BQC**	TIM beta/alpha barrel	Glycosyl hydrolase family 5	**1476429**	
65	**3.2.1.81**	ß-agarase	AGAR_STRCO	**1QOX**	TIM beta/alpha barrel	Glycosyl hydrolase family 1	**3034860**	Predicted two distinct structural folds
	**3.2.1.81**	ß-agarase	AGAA_VIBS7	N/A	N/A	Glycosyl hydrolase family 50	**8285681**	
	**3.2.1.81**	ß-agarase	AGAR_ALTAT	N/A	N/A	Glycosyl hydrolase family 86	**2914859**	

66	**3.2.1.86**	6-phospho-ß-glucosidase	BGLB_ECOLI	**1QOX**	TIM beta/alpha barrel	Glycosyl hydrolase family 1	**3034860**	Two distinct structural folds
	**3.2.1.86**	6-phospho-ß-glucosidase	CHBF_ECOLI	**1S6Y**	Rossmann-fold & LDH C-terminal domain	Glycosyl hydrolase family 4	**10572139**	
67	**3.2.2.21**	DNA-3-methyladenine glycosylase	3MG2_ECOLI	**1MPG**	DNA-glycosylase & TBP-like		**6389535**	Two distinct structural folds
	**3.2.2.21**	DNA-3-methyladenine glycosylase	3MG_HUMAN	**1F4R**	FMT C-terminal domain-like		**8589517, 1645538**	
68	**3.5.1.1**	Asparaginase	ASPG1_YEAST	**1HFW**	Glutaminase/ Asparaginase	Glutaminase/ Asparaginase	**8026756**	At least two distinct structural folds
	**3.5.1.1**	Asparaginase	ASPG_HUMAN	**1APY**	Ntn_hydrolase-like	(Glycosyl)asparaginase	**1840528**	
	**3.5.1.1**	Asparaginase	Q9RFN5_RHIET	N/A	N/A	L-asparaginase II	**10930734**	
69	**3.5.1.4**	Amidase	AMID_PSECL	**1OCK**	Amidase signature	Amidase	**2013568**	Two distinct structural folds
	**3.5.1.4**	Amidase	AMIE_PSEAE	**2UXY**	Carbon-nitrogen hydrolase	Carbon-nitrogen hydrolase	**87219101**	
70	**3.5.1.11**	Penicillin acylase	PAC_ECOLX	**1PNL**	Ntn hydrolase-like	Penicillin amidase	**9292993, 7816145**	Same fold, same supefamily
	**3.5.1.11**	Penicillin acylase	PAC_BACSH	**2PVA**	Ntn hydrolase-like	Linear amide C-N hydrolases	**10331865**	
71	**3.5.1.28**	N-acetylmuramoyl-L-alanine amidase	AMPD_ECOLI	**1J3G**	N-acetylmuramoyl-L-alanine amidase-like	N-acetylmuramoyl-L-alanine amidase-like	**12654266**	Two distinct structural folds
	**3.5.1.28**	N-acetylmuramoyl-L-alanine amidase	AMIB_ECOLI	*1JWQ*	Phosphorylase/ hydrolase-like	Zn-dependent exopeptidases	**7511774**	
72	**3.5.2.6**	ß-Lactamase	AMPC_ECOLI	**2BLS**	Beta-lactamase/ transpeptidase-like	Beta-lactamase	**6795623**	Two distinct structural folds

	**3.5.2.6**	ß-Lactamase	BLAB_BACCE	**1BC2**	Metallo-hydrolase/ oxidoreductase	Zn metallo-beta-lactamase	**9730812**	
73	**3.6.1.1**	Inorganic diphosphatase	IPYR_ARATH	**1TWL**	OB-fold	Pyrophosphatase	**654155**	Four distinct structural folds
	**3.6.1.1**	Inorganic diphosphatase	AVP1_ARATH	N/A	Integral membrane protein	Inorganic H+ pyrophosphatase	**9268385**	
	**3.6.1.1**	Inorganic diphosphatase	PPAC_BACSU	**1WPM**	DHH phosphoesterases	DHH hydrolase	**9845334, 11697905**	
	**3.6.1.1**	Inorganic diphosphatase	PPAX_BACSU	*2HDO*	HAD-like	HAD-like hydrolase	**12359880**	
74	**3.6.1.5**	Apyrase	APY_AEDAE	*1HP1*	5-nucleotidase & Metallo-dependent phosphatases	5'-nucleotidase	**2173922**	Three distinct structural folds
	**3.6.1.5**	Apyrase	ENTP3_HUMAN	*1T6C*	Ribonuclease H-like	Actin-like ATPase domain	**9675246, 8955160**	
	**3.6.1.5**	Apyrase	APY_CIMLE	**2H2N**	5-bladed beta-propeller	Apyrase	**9804829, 15006348**	
75	**3.6.1.11**	Exopolyphosphatase	PPX_ECOLI	**1U6Z**	HD-domain/PDEase-like & Ribonuclease H-like	Ppx associated domain	**8212131**	Two distinct structural folds
	**3.6.1.11**	Exopolyphosphatase	PPX1_YEAST	*1K20*	DHH phosphoesterases	Mn-dependent inorganic pyrophosphatase	**7860598**	
76	**3.6.1.17**	Bis(5'-nucleosyl) -tetraphosphatase (asymmetrical)	APH1_SCHPO	**1FHI**	HIT-like	HIT family	**8554540**	Three distinct structural folds
	**3.6.1.17**	Bis(5'-nucleosyl) -tetraphosphatase (asymmetrical)	AP4A_HUMAN	**1XSA**	Nudix	MutT-like	**7487923**	

	3.6.1.17	Bis(5'-nucleosyl) - tetraphosphatase (asymmetrical)	PRPE_BACSU	*1G5B*	Metallo-dependent phosphatases	Protein serine/ threonine phosphatase	12059787	
77	3.8.1.2	2-Haloalkanoate dehalogenase	HAD1_PSEUC	1QQ5	HAD-like	HAD-like hydrolase	1556080, 10521454	Different stereospecificity
	3.8.1.9	2-Haloalkanoate dehalogenase	HADD_PSEPU	3BJX	N/A	Haloacid dehalogenase DehI	1556080, 18353360	
78	3.8.1.3	Haloacetate dehalogenase	DEH1_MORSB	1G5F	Alpha/beta-hydrolases	Alpha/beta hydrolase_1	1512562, 11939779	Two distinct structural folds
	3.8.1.3	Haloacetate dehalogenase	DEH2_MORSB	1JUD	HAD-like	HAD-like hydrolase	8702766	
79	4.1.1.17	Ornithine decarboxylase	DCOS_ECOLI	1ORD	PLP-dependent transferase	Orn/Lys/Arg decarboxylase, class I	7563080	Two distinct structural folds
	4.1.1.17	Ornithine decarboxylase	DCOR_HUMAN	1D7K	TIM beta/alpha barrel	Orn/Lys/Arg decarboxylase class II	2587220, 2318872	
80	4.1.1.19	Arginine decarboxylase	ADIA_ECOLI	2VYC	PLP-dependent transferase	Orn/Lys/Arg decarboxylase, class I	8383109, 19298070	Three distinct structural folds
	4.1.1.19	Arginine decarboxylase	SPEA_ECOLI	1TWI	TIM beta/alpha barrel	Orn/Lys/Arg decarboxylase class II	4571773	
	4.1.1.19	Arginine decarboxylase	PDAD_METJA	1N2M	Pyruvoyl-dependent histidine and arginine decarboxylases	Pyruvoyl-dependent ArgDC	11980912	
81	4.1.1.22	Histidine decarboxylase	DCHS_ENTAE	*1JS3*	PLP-dependent transferase	Pyridoxal-dependent decarboxylase	2033044	Two distinct structural folds
	4.1.1.22	Histidine decarboxylase	DCHS_LACS3	1HQ6	Pyruvoyl-dependent histidine and arginine decarboxylases	Histidine decarboxylase	2857718	

82	**4.1.1.50**	S-adenosylmethionine decarboxylase	SPED_ECOLI	**1TLU**	S-adenosylmethionine decarboxylase	SAM decarboxylase	**3546296, 17567041**	Same fold, same superfamily
	**4.1.1.50**	S-adenosylmethionine decarboxylase	DCAM_HUMAN	**1MSV**	S-adenosylmethionine decarboxylase	SAM decarboxylase	**10378277**	
83	**4.1.2.13**	Fructose 1,6-bisphosphate aldolase	ALF_ECOLI	**1DOS**	TIM beta/alpha barrel	Fructose-bisphosphate aldolase class II	**8436219, 8836102**	Same fold, same superfamily
	**4.1.2.13**	Fructose 1,6-bisphosphate aldolase	ALDOA_HUMAN	**1ALD**	TIM beta/alpha barrel	Fructose-bisphosphate aldolase class I	**3355497, 2335208**	
84	**4.1.2.15**	Phospho-2-keto-3-deoxyheptonate aldolase	AROG_ECOLI	**1GG1**	TIM beta/alpha barrel	DAHP synthetase I	**10425687**	Same fold, same superfamily
	**4.1.2.15**	Phospho-2-keto-3-deoxyheptonate aldolase	AROF_STRCO	**2B7O**	TIM beta/alpha barrel	Class-II DAHP synthetase	**8760910, 16288916**	
85	**4.1.2.40**	Tagatose 1,6-bisphosphate aldolase	KBAY_ECOLI	**1GVF**	TIM beta/alpha barrel	Fructose-bisphosphate aldolase class II	**11940603**	Same fold, same superfamily
	**4.1.2.40**	Tagatose 1,6-bisphosphate aldolase	LACD_LACLA	**1TO3**	TIM beta/alpha barrel	DeoC/LacD family aldolase	**1901863**	
86	**4.2.1.1**	Carbonate dehydratase	CYNT_ECOLI	*1EKJ*	Resolvase-like	beta-carbonic anhydrase	**1740425**	Three distinct structural folds
	**4.2.1.1**	Carbonate dehydratase	CAH1_HUMAN	**2FOY**	Carbonic anhydrase		**804171, 4207120**	
	**4.2.1.1**	Carbonate dehydratase	CAH_METTE	**1QQ0**	Single-stranded left-handed beta-helix	gamma-carbonic anhydrase-like	**8041719**	
87	**4.2.1.2**	Fumarate hydratase	FUMA_ECOLI	**2ISB**	The swivelling beta/beta/alpha domain	Class I fumarase family	**1917897**	Two distinct structural folds
	**4.2.1.2**	Fumarate hydratase	FUMC_ECOLI	**1FUO**	L-aspartase-like	L-aspartase/fumarase	**8909293, 3282546**	

88	**4.2.1.10**	3-dehydroquinate dehydratase	AROQ_ACTPL	**1UQR**	Flavodoxin-like	Type II 3-dehydroquinate dehydratase	**PubMed**	Two distinct structural folds
	**4.2.1.10**	3-dehydroquinate dehydratase	AROD_ECOLI	**1QFE**	TIM beta/alpha barrel	Class I aldolase	**3541912**	
89	**4.2.1.13**	L-serine ammonia-lyase (now **EC 4.3.1.17**)	SDHL_ECOLI	**2IQQ**	FwdE/GAPDH domain	FeS-dependent L-serine dehydratase	**2504697**	Two distinct structural folds
	**4.2.1.13**	L-serine ammonia-lyase (now **EC 4.3.1.17**)	SDHL_HUMAN	**1PWH**	Tryptophan synthase beta subunit-like PLP-dependent enzymes	DeoC/LacD family aldolase	**14596599**	
90	**4.2.1.51**	Prephenate dehydratase	PHEA_ECOLI	**2QMW**	Periplasmic binding protein-like II	Prephenate dehydratase	**18171624**	Same fold, same superfamily
	**4.2.1.51**	Prephenate dehydratase	PHEC_PSEAE	**1XT8**	Periplasmic binding protein-like II	Bacterial extracellular solute-binding proteins	**92129331**	
91	**4.2.2.2**	Pectate lyase	PEL3_ERWCA	**1PLU**	Single-stranded right-handed beta-helix	Pectate lyase C	**12226275**	Two distinct structural folds, different families
	**4.2.2.2**	Pectate lyase	PELP_PECCC	**2V8K**	Alpha/alpha toroid	Periplasmic pectate lyase	**2695748, 17881361**	
	**4.2.2.2**	Pectate lyase	PELL_ERWCH	**1RU4**	Single-stranded right-handed beta-helix	Polysaccharide lyase family 9	**14670977**	
	**4.2.2.2**	Pectate lyase	Q47471_ERWCA	**3B4N**	*Single-stranded right-handed beta-helix*	Pectate lyase (transeliminase)	**7756691**	
92	**4.2.2.3**	Poly(ß-D-mannuronate) lyase	ALGL_PSEFL	*1QAZ*	Alpha/alpha toroid	Chondroitin AC/ alginate lyase	**12775688**	Three distinct structural folds
	**4.2.2.3**	Poly(ß-D-mannuronate) lyase	ALYA_KLEPN	*1VAV*	Concanavalin A-like lectins/glucanases	Concanavalin A-like lectins/glucanases	**8200539**	
	**4.2.2.3**	Poly(ß-D-mannuronate) lyase	ALYP_PSESO	*1OFM*	Single-stranded right-handed beta-helix		**8336113, 8319887**	

93	**4.2.99.18**	DNA-(apurinic or apyrimidinic site) lyase	END3_ECOLI	**2ABK**	DNA-glycosylase	Endonuclease III	**2669955**	Four distinct structural folds
	**4.2.99.18**	DNA-(apurinic or apyrimidinic site) lyase	APEX1_HUMAN	**1E9N**	DNase I-like	DNase I-like	**1383925, 1380694**	
	**4.2.99.18**	DNA-(apurinic or apyrimidinic site) lyase	APN1_YEAST	*1QTW*	TIM beta/alpha barrel	Endonuclease IV	**3056935**	
	**4.2.99.18**	DNA-(apurinic or apyrimidinic site) lyase	FPG_ECOLI	**1K82**	MutM-like DNA repair proteins	MutM-like DNA repair proteins	**11106507, 14607836, 11912217**	
94	**4.6.1.1**	Adenylate cyclase	CYAA_ECOLI	N/A	N/A	Adenylate_cyclase class I	**6344011, 92011391**	Four distinct structural folds
	**4.6.1.1**	Adenylate cyclase	CYAA_BORPE	**1YRU**	EF-hand-like	Anthrax toxin LF (class II AC)	**2897067, 16138079**	
	**4.6.1.1**	Adenylate cyclase	CYA1_HUMAN	**1CS4**	Ferredoxin-like	Adenylate/guanylate cyclase (class III AC)	**8314585**	
	**4.6.1.1**	Adenylate cyclase	O69199_AERHY	**2FJT**	CYTH-like phosphatases	CYTH domain (class IV AC)	**9642185, 16905149**	
95	**5.2.1.8**	Peptidylprolyl isomerase	PPIA_ECOLI	**1CLH**	Cyclophilin-like	Cyclophilin	**9501079, 17909185**	Two distinct structural folds
	**5.2.1.8**	Peptidylprolyl isomerase	FKBA_ECOLI	**1Q6H**	FKBP-like	FKBP-type PPIase	**1379319**	
96	**5.3.1.8**	Mannose-6-phosphate isomerase	MANA_STRMU	**1QWR**	Double-stranded beta-helix	RmlC-like cupins	**8293960**	Three distinct structural folds
	**5.3.1.8**	Mannose-6-phosphate isomerase	MANA_RHIME	*2GZ6*	Six-hairpin glycosidases	GlcNAc 2-epimerase	**1452036**	
	**5.3.1.8**	Mannose-6-phosphate isomerase	PGMI_SULAC	*1TZB*	SIS domain	SIS_domain	**14551194**	

97	**5.4.2.1**	Phosphoglycerate mutase	GPMA_ECOLI	**1E59**	Phosphoglycerate mutase	Phosphoglycerate mutase	**11038361**	Two distinct structural folds
	**5.4.2.1**	Phosphoglycerate mutase	GPMI_PSESM	**1EJJ**	Alkaline phosphatase-like	2,3-BPG-independent phosphoglycerate mutase	**12076796**	
98	**5.4.2.8**	Phosphomannomutase	RFBK_SALTY	**1K2Y**	Phosphoglucomutase	Phosphoglucomutase	**PubMed**	Two distinct structural folds
	**5.4.2.8**	Phosphomannomutase	PMM1_HUMAN	**2FUC**	HAD-like	Eukaryotic phosphomannomutase	**9119384**	
99	**5.4.99.5**	Chorismate mutase	CHMU_ENTAG	**2AO2**	Chorismate mutase II	Secreted chorismate mutase-like	**8335631, 9497350, 7496534**	Two distinct structural folds
	**5.4.99.5**	Chorismate mutase	CHMU_BACSU	**1COM**	Bacillus chorismate mutase-like	Chorismate mutase	**8378335, 8046752**	
100	**5.5.1.1**	Muconate cycloisomerase	CATB_PSEPU	**1MUC**	TIM beta/alpha barrel & Enolase N-terminal domain	D-glucarate dehydratase-like	**3609743, 3612800**	Two distinct structural folds
	**5.5.1.1**	Muconate cycloisomerase	MLE_TRICU	**1JOF**	7-bladed beta-propeller	WD40/YVTN repeat	**8110801, 11937053**	
101	**5.99.1.2**	DNA topoisomerase	TOP1_ECOLI	**1CY1**	Prokaryotic type I DNA topoisomerase	Prokaryotic type I DNA topoisomerase	**PubMed**	Two distinct structural folds
	**5.99.1.2**	DNA topoisomerase	TOP1_HUMAN	**1LPQ**	DNA-breaking-rejoining enzymes	Eukaryotic DNA topoisomerase I	**98155246, 8747458, 16352556**	
102	**6.1.1.6**	Lysine-tRNA ligase	SYK1_ECOLI	**1BBU**	Class II aaRS and biotin synthetases	Class II aminoacyl-tRNA synthetase	**7735833, 11887185**	Two distinct structural folds
	**6.1.1.6**	Lysine-tRNA ligase	SYK_PYRHO	**1IRX**	Adenine nucleotide alpha hydrolase	Class I lysyl-tRNA synthetase	**9353192, 11887185**	

103	**6.1.1.14**	Glycine-tRNA ligase	SYGA_ECOLI SYGB_ECOLI	**1J5W**	Class II aaRS and biotin synthetases	Glycyl-tRNA synthetase, alpha and beta subunits	**6309809**	Same fold, same superfamily
	**6.1.1.14**	Glycine-tRNA ligase	SYG_HUMAN	**1ATI**	Class II aaRS and biotin synthetases	Class-II aminoacyl-tRNA synthetase	**7753621**	
104	**6.3.2.2**	Glutamate-cysteine ligase	GSH1_ECOLI	**1VA6**	Glutamine synthetase/ guanido kinase	Glutamate-cysteine ligase	**86232579**	Predicted same fold
	**6.3.2.2**	Glutamate-cysteine ligase	GSH1_HUMAN	N/A	*Glutamine synthetase (predicted)*	Glutamate-cysteine ligase	**12663448, 9675072**	
105	**6.3.2.3**	Glutathione synthase	GSHB_ECOLI	**1GSA**	PreATP-grasp domain & ATP-grasp	Prokaryotic glutathione synthetase	**9010922**	Same fold, same superfamily, circular permutation
	**6.3.2.3**	Glutathione synthase	GSHB_HUMAN	**2HGS**	PreATP-grasp domain & ATP-grasp	Eukaryotic glutathione synthetase	**10369661**	
