# Supplementary material for: Non-homologous isofunctional enzymes: A systematic analysis of alternative solutions in enzyme evolution
Source: Biol Direct. 2010 Apr 30;5:31. doi: 10.1186/1745-6150-5-31 (PMC2876114; doi:10.1186/1745-6150-5-31)
Supplement: Additional file 2 — Supplementary Table S2. A new listing of analogous enzymes. The EC numbers are hyperlinked with the ENZYME database entries, protein entries are linked to the NCBI protein database and UniProt, PDB entries - to PDB, SCOP folds - to SCOP, protein superfamilies - to SUPERFAMILY, protein families - to Pfam, and references - to PubMed. [file 1745-6150-5-31-S2.PDF]

Table S2. An updated list of analogous enzymes

This listing was originally prepared as a web supplement to the article by M.Y. Galperin, D.R. Walker and E.V. Koonin **"Analogous enzymes: Independent inventions in enzyme evolution"**, published in 1998 in *Genome Research* **8:** 779-790. Abstract. Free full text. An updted list of the 105 EC nodes described in that paper is available **here**.

This new version is Supplementary Table S2 to the manuscript by M.V. Omelchenko, M.Y. Galperin, Y.I. Wolf and E.V. Koonin **"Non-homologous isofunctional enzymes: A systematic analysis of alternative solutions in enzyme evolution**, submitted for publication in *Biology Direct*.

No.	EC no.	Enzyme name	NCBI	UniProt	PDB	SCOP fold	Super-family	Family (Pfam)	Ref.	Comments
1	**1.1.1.1**	Alcohol dehydrogenase	P10127	ADH4_YEAST	2BI4	Dehydroquinate synthase-like	SupFam	Iron-contaning alcohol dehydrogenase	3282541, 2823079	Two distinct structural folds
	**1.1.1.1**	Alcohol dehydrogenase	P00334	ADH_DROME	1MG5	Rossmann-fold	SupFam	Short chain dehydrogenase	15016358, 11669613	
2	**1.1.1.2**	Alcohol dehydrogenase (NADP +)	P0A4X1	ADHC_MYCBO	1YQD	GroES-like and Rossmann-fold	SupFam	Zinc-contaning alcohol dehydrogenase	1427101, 15829607	Three distinct structural folds
	**1.1.1.2**	Alcohol dehydrogenase (NADP +)	P14550	AK1A1_HUMAN	2ALR	TIM beta/alpha-barrel	SupFam	Aldo/keto reductase	15299353, 10486210	
	**1.1.1.2**	Alcohol dehydrogenase (NADP +)	Q09669	ADH4_SCHPO	1O2D	Dehydroquinate synthase-like	SupFam	Iron-contaning alcohol dehydrogenase	15327949, 14705036	
3	**1.1.1.27**	L-lactate dehydrogenase	P00338	LDHA_HUMAN	1EZ4	Rossmann-fold and LDH C-terminal domain-like	SupFam	Lactate and malate dehydrogenases	11807949	Two distinct structural folds
	**1.1.1.27**	L-lactate dehydrogenase	Q07251	LDH_RALEH	1VBI	L-sulfolactate dehydrogenase-like	SupFam	Malate/L-Lactate dehydrogenases	8405966	
4	**1.1.1.28**	D-lactate dehydrogenase	P52643	LDHD_ECOLI	2DLD	Rossmann-fold and Flavodoxin-like	SupFam	Formate/glycerate dehydrogenases	9025293	Two distinct structural folds
	**1.1.1.28**	D-lactate dehydrogenase	P06149	DLD_ECOLI	1F0X	FAD-binding and Ferredoxin-like	SupFam	D-lactate dehydrogenase	10944213	
5	**1.1.1.37**	Malate dehydrogenase	Q07841	MDH_HALMA	2J5K	Rossmann-fold and LDH C-terminal domain-like	SupFam	L-lactate/malate dehydrogenases	8476859, 10653643	Two distinct structural folds
	**1.1.1.37**	Malate dehydrogenase	P16142	MDH_METFE	2G8Y	L-sulfolactate dehydrogenase-like	SupFam	Malate/L-Lactate dehydrogenases	2110059, 10850983	
6	**1.1.1.50**	3-alpha-hydroxysteroid dehydrogenase (B-specific)	Q9ZFY9	Q9ZFY9_COMTE (DIDH_COMTE)	1FJH	Rossmann-fold	SupFam	Short chain dehydrogenase	8944761, 10833462, 11007791	Two distinct structural folds

	**1.1.1.50**	3-alpha-hydroxysteroid dehydrogenase (B-specific)	P17516	AK1C4_HUMAN	2FVL	TIM beta/alpha-barrel	SupFam	Aldo/keto reductase	1530633, 11158055	
7	**1.1.1.62**	Estradiol 17-ß-dehydrogenase	P14061	DHB1_HUMAN	1FDS	Rossmann-fold	SupFam	Short chain dehydrogenase	2846351, 7663947	Two distinct structural folds
	**1.1.1.62**	Estradiol 17-ß-dehydrogenase	P70694	DHB5_MOUSE	1Q5M	TIM beta/alpha-barrel	SupFam	Aldo/keto reductase	7737980	
8	**1.1.1.63**	Testosterone 17-ß-dehydrogenase	O14756	H17B6_HUMAN	similar to 1I01	Rossmann-fold	SupFam	Short-chain dehydrogenase	11513953, 11360992	Two distinct structural folds
	**1.1.1.63**	Testosterone 17-ß-dehydrogenase	P42330	AK1C3_HUMAN	1S1P	TIM beta/alpha-barrel	SupFam	Aldo/keto reductase	14996743, 9927279, 15087468	
9	**1.1.1.64**	Testosterone 17-ß-dehydrogenase ($NADP^+$)	P37058	DHB3_HUMAN	2C07	Rossmann-fold	SupFam	Short-chain dehydrogenases	2558148	Two distinct structural folds
	**1.1.1.64**	Testosterone 17-ß-dehydrogenase ($NADP^+$)	P42330	AK1C3_HUMAN	1S1P	TIM beta/alpha-barrel	SupFam	Aldo/keto reductase	14996743, 9927279	
10	**1.1.1.82**	Malate dehydrogenase ($NADP^+$)	Q60176	MDH_METJA	1HYG	Rossmann-fold and LDH C-terminal domain-like	SupFam	Lactate/malate dehydrogenase	10850983, 11292347	Two distinct structural folds
	**1.1.1.82**	Malate dehydrogenase ($NADP^+$)	P16142	MDH_METFE	1RFM	L-sulfolactate dehydrogenase-like	SupFam	Malate/L-lactate dehydrogenase	2110059	
11	**1.1.1.189**	Prostaglandin-E2 9-reductase	P16152	CBR1_HUMAN	1WMA	Rossmann-fold	SupFam	Short-chain dehydrogenase	8421682	Two distinct structural folds
	**1.1.1.189**	Prostaglandin-E2 9-reductase	P80508	PE2R_RABIT	1Q13	TIM beta/alpha-barrel	SupFam	Aldo/keto reductase	8529651	
12	**1.1.1.283**	Methylglyoxal reductase	O32210	GR_BACSU	1MZR	TIM beta/alpha-barrel	SupFam	Aldo/keto reductase	16232966	Two distinct structural folds
	**1.1.1.283**	Methylglyoxal reductase	Q12068	GRE2_YEAST	1Y1P	Rossmann-fold	SupFam	NAD dependent epimerase/dehydratase	3896793, 12722185	
13	**1.2.1.2**	Formate dehydrogenase	P07658	FDHF_ECOLI	1FDI	Formate dehydrogenase/ DMSO reductase	SupFam	Molybdopterin-containing oxidoreductases	9036855	Two distinct structural folds
	**1.2.1.2**	Formate dehydrogenase	P33160	FDH_PSESR	2NAC	Rossmann-fold and Flavodoxin-like	SupFam	2-hydroxyacid dehydrogenase	1597184, 8484798	
14	**1.2.1.10**	Acetaldehyde dehydrogenase (acetylating)	P77580	MHPF_ECOLI, MHPF_PSEPU	1NVM	Rossmann-fold and FwdE/ GAPDH domain-like	SupFam	Prokaryotic acetaldehyde dehydrogenase	16782065, 11264589, 12764229	Two distinct structural folds
	**1.2.1.10**	Acetaldehyde dehydrogenase (acetylating)	Q24803	ADH2_ENTHI	N-term is similar to 1AD3	Predicted: ALDH-like	SupFam	Aldehyde dehydrogenase	7980441	
	**1.2.99.2**	Carbon-monoxide dehydrogenase (acceptor)	P19919	DCML_OLICO	1N60	CO dehydrogenase ISP C-domain like and others	SupFam	Aldehyde oxidase and xanthine dehydrogenase	10482497, 12475995	Two distinct

15	**1.2.99.2**	Carbon-monoxide dehydrogenase (acceptor)	P31896	COOS_RHORU	1JQK	Prismane protein-like	SupFam	Prismane/CO dehydrogenase	8561463, 11593006	structural folds
16	**1.3.1.34**	2,4-dienoyl-CoA reductase (NADPH)	P42593	FADH_ECOLI	1PS9	TIM beta/alpha-barrel	SupFam	Oxidoreductase / NADH oxidase family	12840019	Two distinct structural folds
	**1.3.1.34**	2,4-dienoyl-CoA reductase (NADPH)	Q9NUI1	DECR2_HUMAN	1W6U	Rossmann-fold	SupFam	Short-chain dehydrogenases	15531764	
17	**1.5.1.29**	FMN reductase	P0AEN1	FRE_ECOLI	1QFJ	Reductase/isomerase/ elongation factor common domain and Ferredoxin reductase-like C-terminal NADP-linked domain	SupFam	NAD(P)H-flavin reductase	2050627, 10353815	Two distinct structural folds
	**1.5.1.29**	FMN reductase	Q07923	LOT6_YEAST	1T0I	Flavodoxin-like	SupFam	FMN reductase	15184374	
18	**1.5.1.34**	6,7-dihydropteridine reductase	P09417	DHPR_HUMAN	1HDR	Rossmann-fold	SupFam	Short-chain dehydrogenase	3033643	Two distinct structural folds
	**1.5.1.34**	6,7-dihydropteridine reductase	P38489	NFNB_ECOLI	1ICR	FMN-dependent nitroreductase-like	SupFam	Nitroreductase	3060113, 11491290, 15684426	
19	**1.6.99.3**	NADH dehydrogenase	P00393	DHNA_ECOLI	1XHC	FAD/NAD(P)-binding domain	SupFam	NADH dehydrogenase	1917890	Two distinct structural folds
	**1.6.99.3**	NADH dehydrogenase	P28331	NDUS1_HUMAN	2FUG	Formate dehydrogenase/ DMSO reductase	SupFam	Complex I 75 kDa subunit family	1935949	
20	**1.7.2.1**	Nitrite reductase (NO-forming)	P38501	NIR_ALCFA	2AFN	Cupredoxin-like	SupFam	Multicopper oxidase	8515232	Two distinct structural folds
	**1.7.2.1**	Nitrite reductase (NO-forming)	P24474	NIRS_PSEAE	1NIR	Cytochrome c and 8-bladed beta-propeller	SupFam	Cytochrome D1 heme domain (Complex I 75 kDa subunit family)	2506077, 11226222	
21	**1.11.1.5**	Cytochrome-c peroxidase	P14532	CCPR_PSEAE	1EB7	Cytochrome c	SupFam	Di-heme cytochrome c peroxidase	7781769, 1657179, 8591033	Two distinct structural folds
	**1.11.1.5**	Cytochrome-c peroxidase	P00431	CCPR_YEAST	1EBE	Heme-dependent peroxidases	SupFam	Peroxidase	2169873, 6092361	
22	**1.11.1.6**	Catalase	P04040	CATA_HUMAN	1QQW	Heme-dependent catalase-like	SupFam	Heme-dependent catalase	10666617, 3755525	Three distinct structural folds
	**1.11.1.6**	Catalase	P13029	CATA_ECOLI	2FXG	Heme-dependent peroxidases	SupFam	Peroxidase	374409, 2670897, 8508796	
	**1.11.1.6**	Catalase	P60355	MCAT_LACPL	1JKU	Ferritin-like	SupFam	Manganese catalase	8939876	

23	**1.11.1.7**	Peroxidase	O08709	PRDX6_MOUSE	1PRX	Thioredoxin fold	SupFam	Thioredoxin	9291135, 10395907, 9587003	At least two distinct structural folds
	**1.11.1.7**	Peroxidase	P05164	PERM_HUMAN	1MYP	Heme-dependent peroxidases	SupFam	Animal heme peroxidase	2154223	
	**1.11.1.7**	Peroxidase	P31545	YCDB_ECOLI	similar to 2d3q	Predicted: Ferredoxin-like	SupFam	Dyp-type peroxidase-like	16551627, 14684913	
24	**1.11.1.10**	Chloride peroxidase	P25026	PRXC_PSEPY	1A88	Alpha/beta hydrolase	SupFam	Haloperoxidase	8344520	Two distinct structural folds
	**1.11.1.10**	Chloride peroxidase	P49053	PRXC_CURIN	1VNC	Acid phosphatase/ Vanadium-dependent haloperoxidase	SupFam	Type 2 phosphatidic acid phosphatase (PAP2)	9165086	
	**1.11.1.10**	Chloride peroxidase	P04963	PRXC_CALFU	2CPO	EF Hand-like	SupFam	Cloroperoxidase	8747463, 11278701	
25	**1.11.1.15**	Peroxiredoxin	Q9Y9L0	TDXH_AERPE	2E2G	Thioredoxin fold	SupFam	AhpC/TSA family	18436649	Two distinct structural folds
	**1.11.1.15**	Peroxiredoxin	P0A5N4	AHPD_MYCTU	1KNC	AhpD-like	SupFam	Carboxymuconolactone decarboxylase	11914371, 10766746, 11799204, 12761216	
	**1.11.1.15**	Peroxiredoxin	P0C0L2	OSMC_ECOLI	1NYE	OsmC-like	SupFam	OsmC-like	14627744, 15103136	
26	**1.12.2.1**	Cytochrome-c3 hydrogenase (was 1.18.99.1)	P12944	PHNL_DESGI	1FRV	HydA/Nqo6-like and HydB/ Nqo4-like	SupFam	Ni-Fe hydrogenase	2651421, 7854413	Two distinct structural folds
	**1.12.2.1**	Cytochrome-c3 hydrogenase (was 1.18.99.1)	P29166	PHF1_CLOPA	1FEH	Fe-only hydrogenase	SupFam	Iron-only hydrogenase	1911757, 9836629	
27	**1.14.99.3**	Heme oxygenase	P09601	HMOX1_HUMAN	1N3U	Heme oxygenase-like	SupFam	Heme oxygenase	12500973, 15285018, 15049686, 15522396	Two distinct structural folds
	**1.14.99.3**	Heme oxygenase	Q6GKE0	ISDI_STAAR	1XBW	Ferredoxin-like	SupFam	Antibiotic biosynthesis monooxygenase	14570922, 15774886	
28	**1.15.1.1**	Superoxide dismutase	P09157	SODF_ECOLI	1ISA	FeMn superoxide dismutase	SupFam	Fe,Mn superoxide dismutase	2447093	Two distinct structural folds
	**1.15.1.1**	Superoxide dismutase	P53635	SODC_ECOLI	1ESO	Immunoglobulin-like beta-sandwich	SupFam	Cu,Zn superoxide dismutase	9405149	
	**1.15.1.1**	Superoxide dismutase	P80734	SODN_STRSO	1Q0D	Four-helical up-and-down bundle	SupFam	Nickel superoxide dismutase	15173586	

29	**1.16.3.1**	Ferroxidase	P02794	FRIH_HUMAN, FTNA_ECOLI	2CEI, 1BCF	Ferritin-like	SupFam	Ferritin, Ferritin-like domain	7982945, 15201052	Two distinct structural folds
	**1.16.3.1**	Ferroxidase	P00450	CERU_HUMAN	1KCW	Cupredoxin-like	SupFam	Multicopper oxidase	17242517, 12055353	
30	**1.18.1.2**	Ferredoxin-NADP^{+} reductase	Q44532	FENR_AZOVI	1A8P	Reductase/isomerase/ elongation factor common domain and Ferredoxin reductase-like C-terminal NADP-linked domain	SupFam	Oxidoreductase FAD-binding and NAD-binding domains	8034707, 9865948, 9149148, 8449868	Two distinct structural folds
	**1.18.1.2**	Ferredoxin-NADP^{+} reductase	O05783	FPRA_MYCTU	1LQT	FAD/NADP-binding domain and Nucleotide-binding domain	SupFam	Pyridine nucleotide-disulphide oxidoreductase	12071965	
31	**1.20.4.1**	Arsenate reductase (glutaredoxin)	P08692	ARSC1_ECOLX	1I9D	Thioredoxin fold	SupFam	ArsC family	11709171	Two distinct structural folds
	**1.20.4.1**	Arsenate reductase (glutaredoxin)	Q06597	ACR2_YEAST	1C25	Rhodanese/Cell cycle control phosphatase	SupFam	Rhodanese	10801893	
32	**2.1.1.43**	Histone-lysine N-methyltransferase	Q8WTS6	SETD7_HUMAN	1N6C	Beta-clip (catalytic domain)	SupFam	Histone-lysine methyltransferase	12540855, 12372304	Two distinct structural folds
	**2.1.1.43**	Histone-lysine N-methyltransferase	Q04089	DOT1_YEAST	1U2Z	S-adenosyl-L-methionine-dependent methyltransferases	SupFam	Histone methylation protein	15292170, 12086673, 12080090	
33	**2.3.1.28**	Chloramphenicol O-acetyltransferase	P23364	CAT4_AGRTU	1XAT	Single-stranded left-handed beta-helix	SupFam	Galactoside acetyltransferase	2013403, 9578552	Two distinct structural folds
	**2.3.1.28**	Chloramphenicol O-acetyltransferase	P00484	CAT3_ECOLX	3CLA	CoA-dependent acyltransferases	SupFam	Chloramphenicol acetyltransferase	2187098	
34	**2.3.2.5**	Glutaminyl-peptide cyclotransferase	Q16769	QPCT_HUMAN	2AFM	Phosphorylase/hydrolase-like	SupFam	Peptidase family M28	16135565, 15063747	Two distinct structural folds
	**2.3.2.5**	Glutaminyl-peptide cyclotransferase	O81226	O81226_CARPA	2FAW, similar to 1VKD	5-bladed beta-propeller (paper)	SupFam	Glutamine cyclotransferase	11035947, 17081122	
35	**2.4.1.25**	4-alpha-glucanotransferase	P15977	MALQ_ECOLI	similar to 1FP8	TIM beta/alpha-barrel	SupFam	Glycoside hydrolase family 77	2845225	Two distinct structural folds
	**2.4.1.25**	4-alpha-glucanotransferase	O32462	MALQ_THELI	1K1X	7-stranded beta/alpha barrel	SupFam	Glycosyl hydrolase family 57	12618437	
36	**2.5.1.3**	Thiamine-phosphate synthase	P30137	THIE_ECOLI	2TPS	TIM beta/alpha-barrel	SupFam	Thiamine monophosphate synthase	10350464	Two distinct structural folds (no EC)

	**2.5.1.3**	Thiamine-phosphate synthase	O26949	O26949_METTH	2PHP	AraD/HMP-PK domain-like	SupFam	Archaeal phosphomethylpyrimidine kinase	12794638	
37	**2.5.1.17**	Cob(I)yrinic acid a,c-diamide adenosyltransferase	P31570	BTUR_SALTY	1G5T	P-loop containing NTPases	SupFam	ATP:corrinoid adenosyltransferase BtuR/ CobO/CobP	7860601	Two distinct structural folds
	**2.5.1.17**	Cob(I)yrinic acid a,c-diamide adenosyltransferase	Q96EY8	MMAB_HUMAN	2IDX	Ferritin-like	SupFam	Cobalamin adenosyltransferase	12514191, 17176040	
38	**2.5.1.18**	Glutathione transferase	P39100	GST_ECOLI, GSTK1_HUMAN	1A0F, 1R4W	Thioredoxin fold and GST C-terminal domain-like	SupFam	Glutathione S-transferase, class beta, class kappa	2185038, 16081649, 14717589	Three distinct structural folds
	**2.5.1.18**	Glutathione transferase	P10620	MGST1_HUMAN	2H8A	MAPEG domain-like	SupFam	MAPEG family	8812420, 9278457, 10091672, 16806268	
	**2.5.1.18**	Glutathione transferase	Q56415	FOSA_SERMA	1NPB	Glyoxalase/Bleomycin resistance protein/ Dihydroxybiphenyl dioxygenase	SupFam	Antibiotic resistance proteins	15075406, 9115979	
39	**2.5.1.47**	Cysteine synthase	P47998	CYSK1_ARATH	1Z7W	Tryptophan synthase beta subunit-like PLP-dependent enzymes	SupFam	Pyridoxal-phosphate dependent enzyme	16166087	Two distinct structural folds
	**2.5.1.47**	Cysteine synthase	P06106	MET17_YEAST	1GC0	PLP-dependent transferase-like	SupFam	Cys/Met metabolism PLP-dependent enzyme	7765825, 8511969	
40	**2.7.1.4**	Fructokinase	P40713	SCRK_ECOLI	similar to 1TZ6	Ribokinase-like	SupFam	PfkB family carbohydrate kinase	8278523	Two distinct structural folds
	**2.7.1.4**	Fructokinase	Q07211	SCRK_STRMU	1XC3	Ribonuclease H-like motif	SupFam	Actin-like ATPase domain	8336109	
41	**2.7.1.11**	6-phosphofructokinase	P06998	K6P1_ECOLI	1PFK	Phosphofructokinase	SupFam	Phosphofructokinase	2975709	Two distinct structural folds
	**2.7.1.11**	6-phosphofructokinase	P06999	K6PF2_ECOLI	3CQD	Ribokinase-like	SupFam	PfkB family carbohydrate kinase	18762190, 6310120	
42	**2.7.1.12**	Gluconokinase	P46859	GNTK_ECOLI	1KNQ	P-loop containing NTPases	SupFam	Gluconate kinase	8655507, 11468405	Two distinct structural folds
	**2.7.1.12**	Gluconokinase	P12011	GNTK_BACSU	3GBT, 2ITM	Ribonuclease H-like motif	SupFam	Glycerol kinase	3020045	
	**2.7.1.31**	Glycerate kinase	P23524	GLXK2_ECOLI	1TO6	Glycerate kinase I	SupFam	Glycerate kinase type-1	5325263, 166273	Two distinct structural folds

43	**2.7.1.31**	Glycerate kinase	Q944I4	GLYK_ARATH	similar to 1ODF	P-loop containing NTPases	SupFam	PRK/uridine kinase	15980259	
	2.7.1.31	Glycerate kinase	Q8IVS8	GLCTK_HUMAN	smilar to 2B8N	GckA/TtuD-like	SupFam	Glycerate kinase type-2 (contains MOFRL domain)	16753811, 16865707	Produces 2-phospho-glycerate
44	**2.7.1.33**	Pantothenate kinase	O25533	COAX_HELPY	2NRH	Ribonuclease H-like motif	SupFam	Type III pantothenate kinase	15795230, 16905099	Two distinct structural folds
	**2.7.1.33**	Pantothenate kinase	P0A6I3	COAA_ECOLI	1SQ5	P-loop containing NTPases	SupFam	PRK/Uridine kinase	15136582, 1328157	
45	**2.7.1.39**	Homoserine kinase	P17423	KHSE_YEAST	1FWK	Ribosomal protein S5 domain 2-like and Ferredoxin-like	SupFam	GHMP kinase	2165904	Two distinct structural folds
	**2.7.1.39**	Homoserine kinase	P29364	KHSE_PSEAE	2PPQ	Protein kinase-like (PK-like)	SupFam	Phosphotransferase	1333566	
46	**2.7.1.71**	Shikimate kinase	P0A6E1	AROL_ECOLI	1SHK	P-loop containing NTPases	SupFam	Shikimate kinase	3001029, 9600856, 11369852	Two distinct structural folds
	**2.7.1.71**	Shikimate kinase	Q58835	AROK_METJA	similar to 1VIS	Ribosomal protein S5 domain 2-like and Ferredoxin-like	SupFam	GHMP kinase	11114929	
47	**2.7.1.107**	Diacylglycerol kinase	P49619	DGKG_HUMAN	2QV7	NAD kinase/diacylglycerol kinase-like	SupFam	Diacylglycerol kinase catalytic domain	18611377	Two distinct structural folds
	**2.7.1.107**	Diacylglycerol kinase	P0ABN1	KDGL_ECOLI	-	Integral membrane protein	SupFam	Prokaryotic diacylglycerol kinase	2984194, 8071224	
48	**2.7.4.1**	Polyphosphate kinase	P0A7B1	PPK_ECOLI	1XDO	Spectrin repeat-like and Phospholipase D/nuclease and PHP14-like	SupFam	Polyphosphate kinase	8380170, 1331061	Two distinct structural folds
	**2.7.4.1**	Polyphosphate kinase	Q8GCQ3	Q8GCQ3_PSEAE (PPK2)	3CZQ	3-layer Alpha/beta/alpha sandwich fold (paper), contains P-loop	SupFam	Polyphosphate kinase 2	12486232, 19001261	
49	**2.7.4.2**	Phosphomevalonate kinase	P24521	ERG8_YEAST	similar to 1FWL	Ribosomal protein S5 domain 2-like and Ferredoxin-like	SupFam	GHMP_kinases	1846667	Two distinct structural folds
	**2.7.4.2**	Phosphomevalonate kinase	Q15126	PMVK_HUMAN	3CH4	P-loop containing NTPases	SupFam	Phosphomevalonate kinase	10191291, 16519518, 17902708	
50	**2.7.10.2**	Non-specific protein-tyrosine kinase	P06241	FYN_HUMAN	2H8H	Protein kinase-like (PK-like)	SupFam	Protein tyrosine kinase	15557120	At least two distinct

	**2.7.10.2**	Non-specific protein-tyrosine kinase	Q54520	CPSD2_STRPN	similar to 1G3Q	P-loop containing NTPases	SupFam	Pfam hit	10760144	structural folds
	**2.7.10.2**	Non-specific protein-tyrosine kinase	Q9UIG0	BAZ1B_HUMAN	-	-	SupFam	Pfam hit	19092802	
51	**3.1.1.2**	Arylesterase	Q07792	ESTE_VIBMI	similar to 1IVN	Flavodoxin-like	SupFam	GDSL-like Lipase/ Acylhydrolase	8141782	Two distinct structural folds
	**3.1.1.2**	Arylesterase	P22862	ESTE_PSEFL	1VA4	Alpha/beta hydrolase	SupFam	Alpha/beta hydrolase	15213385, 7704276, 1368608	
	**3.1.1.2**	Arylesterase	P27169	PON1_HUMAN	1V04	6-bladed beta-propeller	SupFam	Arylesterase	8393742, 1718413, 1673382, 15098021	
52	**3.1.1.4**	Phospholipase A2	Q9NZK7	PA2GE_HUMAN	1POE	Phospholipase A2 PLA2	SupFam	Phospholipase A2	10681567	Two distinct structural folds
	**3.1.1.4**	Phospholipase A2	P0A921	PA1_ECOLI	1QD5	Transmembrane beta-barrels	SupFam	Phospholipase A1	8300539, 10537112	
	**3.1.1.4**	Phospholipase A2	P47712	PA24A_HUMAN	1CJY	FabD/lysophospholipase-like (catalytic domain)	SupFam	Lysophospholipase catalytic domain	10319815, 8083230, 11416127	
53	**3.1.1.5**	Lysophospholipase	O88202	LPP60_RAT	N-term is similar to 2OCD	Glutaminase/Asparaginase	SupFam	Asparaginase (N-term)	9575212	At least two distinct structural folds
	**3.1.1.5**	Lysophospholipase	P47712	PA24A_HUMAN	1CJY	FabD/lysophospholipase-like (catalytic domain)	SupFam	Lysophospholipase catalytic domain	10319815, 8083230, 11416127	
	**3.1.1.5**	Lysophospholipase	P07000	PLDB_ECOLI	similar to 1A8Q	Predicted: Alpha/beta hydrolase	SupFam	Alpha/beta hydrolase	3908447, 3908445	
54	**3.1.1.29**	Aminoacyl-tRNA hydrolase	P0A7D1	PTH_ECOLI	2PTH	Phosphorylase/hydrolase-like	SupFam	Peptidyl-tRNA hydrolase	9303320	Two distinct structural folds
	**3.1.1.29**	Aminoacyl-tRNA hydrolase	Q980V1	PTH_SULSO, PTH2_HUMAN	1XTY, 1Q7S	Peptidyl-tRNA hydrolase II	SupFam	Peptidyl-tRNA hydrolase PTH2	12799450, 15766258, 14660562	
55	**3.1.1.31**	6-phosphogluconolactonase	Q9EV79	6PGL_PSEPU, 6PGL_THEMA	similar to 1VL1	NagB/RpiA/CoA transferase-like	SupFam	NagB-like	10869070, 11277923, 16359314	Two distinct structural folds
	**3.1.1.31**	6-phosphogluconolactonase	P52697	6PGL_ECOLI	1RI6	7-bladed beta-propeller	SupFam	Muconate lactonizing enzyme	15576773, 16359314	

56	**3.1.1.61**	Protein-glutamate methylesterase	P07330	CHEB_ECOLI	1CHD	Methylesterase CheB, C-terminal domain	SupFam	CheB methylesterase	7608974	Two distinct structural folds
	**3.1.1.61**	Protein-glutamate methylesterase	Q9X005	CHED_THEMA	2F9Z	CheC-like and CNF1/YfiH-like putative cysteine hydrolases	SupFam	CheD	16469702	
57	**3.1.2.1**	Acetyl-CoA hydrolase	Q9UUJ9	ACH1_SCHPO	2G39	NagB/RpiA/CoA transferase-like	SupFam	Acetyl-CoA hydrolase/ transferase	12606555, 1970569	Two distinct structural folds
	**3.1.2.1**	Acetyl-CoA hydrolase	Q99NB7	ACO12_RAT	3B7K (N-term) 3FO5 (C-term)	Thioesterase/thiol ester dehydrase-isomerase	SupFam	Thioesterase superfamily	11322891	
58	**3.1.3.2**	Acid phosphatase	Q9NPH0	PPA6_HUMAN	similar to 1ND5	Phosphoglycerate mutase-like	SupFam	Histidine acid phosphatase	12010880, 10506173	At least five distinct structural folds
	**3.1.3.2**	Acid phosphatase	P0AE22	APHA_ECOLI	1N8N	HAD-like	SupFam	Class B acid phosphatases	9011040	
	**3.1.3.2**	Acid phosphatase	P14924	PPA_ZYMMO	similar to 1D2T	Acid phosphatase/ Vanadium-dependent haloperoxidase	SupFam	Type 2 phosphatidic acid phosphatase (PAP2)	2914872	
	**3.1.3.2**	Acid phosphatase	P13686	PPA5_HUMAN	1UTE	Metallo-dependent phosphatases	SupFam	Calcineurin-like phosphoesterase	2775236	
	**3.1.3.2**	Acid phosphatase	P24666	PPAC_HUMAN	5PNT	Phosphotyrosine protein phosphatases I-like	SupFam	Low molecular weight phosphotyrosine protein phosphatase	9705307	
	**3.1.3.2**	Acid phosphatase	P37274	PHOA_PENCH	-	-	SupFam	Phosphoesterase	1563629	
59	**3.1.3.5**	5'-nucleotidase	P0A840	SURE_ECOLI	1L5X, 2V4O	SurE-like	SupFam	SurE nucleotidase	19021761, 15489502	Two distinct structural folds
	**3.1.3.5**	5'-nucleotidase	B1X903	YFBR_ECODH	2PAQ	HD-domain/PDEase-like	SupFam	Metal dependent phosphohydrolase	15489502	
	**3.1.3.5**	5'-nucleotidase	O46411	5NTC_BOVIN	2J2C, similar to 2BDE	HAD-like	SupFam	5' nucleotidase	9371705	
60	**3.1.3.8**	3-phytase	P42094	PHYT_BACSU	1POO	6-bladed beta-propeller	SupFam	Phytase	11104666	Two distinct structural folds
	**3.1.3.8**	3-phytase	O00092	PHYA_ASPFU	1QWO	Phosphoglycerate mutase-like	SupFam	Histidine acid phosphatase	9143104, 15136045	
61	**3.1.3.11**	Fructose-bisphosphatase	P19912	F16P2_RALEH	similar to 1D9Q	Carbohydrate phosphatase	SupFam	FBPase	7767230	At least two distinct

	**3.1.3.11**	Fructose-bisphosphatase	Q45597	F16P_BACSU	-	Predicted: Metallo-dependent phosphatases	SupFam	Firmicute fructose-1,6-bisphosphatase (FBPase 2)	221467, 9696785	structural folds
	**3.1.3.11**	Fructose-bisphosphatase	Q8U359	Q8U359_PYRFU (PF0613)	1UMG	Sulfolobus fructose-16-bisphosphatase-like	SupFam	FBPase 3	12029059, 15274916, 12065581, 15317785	
62	**3.1.3.15**	Histidinol-phosphatase	P06987	HIS7_ECOLI	2FPR	HAD-like	SupFam	Polynucleotide kinase 3 phosphatase	16966333	Two distinct structural folds
	**3.1.3.15**	Histidinol-phosphatase	O34411	HIS9_BACSU	3DCP	7-stranded beta/alpha barrel	SupFam	Polymerase/Histidinol Phosphatase	10322033, 17929834	
63	**3.1.3.16**	Phosphoprotein phosphatase	P55798	PRP1_ECOLI	1G5B	Metallo-dependent phosphatases	SupFam	Calcineurin-like phosphoesterase	9130712	Four distinct structural folds
	**3.1.3.16**	Phosphoprotein phosphatase	O34779	PRPC_BACSU	2PK0, similar to C-term of 1TXO	PP2C-like	SupFam	Protein phosphatase 2C	10986276	
	**3.1.3.16**	Phosphoprotein phosphatase	Q9GZU7	CTDS1_HUMAN	1TA0	HAD-like	SupFam	NLI interacting factor-like phosphatase	15304220	
	**3.1.3.16**	Phosphoprotein phosphatase	Q8WTR2	DUS19_HUMAN	similar to 1M3G	Phosphotyrosine protein phosphatases II	SupFam	Dual specificity phosphatase (catalytic domain)	12479873	
64	**3.1.3.33**	Polynucleotide 5'-phosphatase	O13297	CET1_YEAST	1D8H	CYTH-like phosphatases	SupFam	mRNA capping enzyme, beta chain	9345280, 10589681	At least two distinct structural folds
	**3.1.3.33**	Polynucleotide 5'-phosphatase	O60942	MCE1_HUMAN	N-term is 1I9S and C-term is similar to 1P16	Phosphotyrosine protein phosphatases II	SupFam	Dual specificity phosphatase, catalytic domain	9473487, 9371772, 9512541	
	**3.1.3.33**	Polynucleotide 5'-phosphatase	P04298	MCEL_VACCW	-	-	SupFam	Viral GTase family (UniProt)	8662636, 8662635	
	**3.1.3.33**	Polynucleotide 5'-phosphatase	P08411	POLN_SFV	-	-	SupFam	Pfam hits	10748213	
65	**3.1.3.48**	Protein-tyrosine-phosphatase	P53433	PTPA_STRCO	1D1P	Phosphotyrosine protein phosphatases I-like	SupFam	Low-molecular-weight phosphotyrosine protein phosphatases	8550407, 1304913	At least three distinct
	**3.1.3.48**	Protein-tyrosine-phosphatase	P23468	PTPRD_HUMAN	1LAR (C-term)	Phosphotyrosine protein phosphatases II	SupFam	Higher-molecular-weight phosphotyrosine protein phosphatases	2170109, 8833149	

	**3.1.3.48**	Protein-tyrosine-phosphatase	P30307	MPIP3_HUMAN	1QB0 (C-term)	Rhodanese/Cell cycle control phosphatase	SupFam	Cell cycle control phosphatase, catalytic domain	8276463	structural folds
	**3.1.3.48**	Protein-tyrosine-phosphatase	P96717	YWQE_BACSU	similar to 2anu	Predicted: 7-stranded beta/alpha barrel	SupFam	PHP domain	15866923	
	**3.1.3.48**	Protein-tyrosine-phosphatase	P97480	EYA3_MOUSE	similar to 1jud	Predicted: HAD-like	SupFam	HAD-like hydrolase	14628042, 14628052	
66	**3.1.4.52**	Cyclic-diguanylate-specific phosphodiesterase	P21514	YAHA_ECOLI	2BAS	TIM beta/alpha-barrel	SupFam	Pfam hits	15994307, 15995192	Two distinct structural folds (EC 3.1.4.-)
	**3.1.4.52**	Cyclic-diguanylate-specific phosphodiesterase	Q4UU85	RPFG_XANC8	similar to 1F0J	HD-domain/PDEase-like	SupFam	HD domain	16611728	
67	**3.1.6.1**	Arylsulfatase	P51691	ARS_PSEAE	1HDH	Alkaline phosphatase-like	SupFam	Sulfatase	7744061, 11435113	Two distinct structural folds
	**3.1.6.1**	Arylsulfatase	P28607	ARS_ALTCA	similar to 2CBN	Metallo-hydrolase/oxidoreductase	SupFam	Metallo-beta-lactamase	8535517	
68	**3.1.8.1**	Aryldialkylphosphatase	P0A434	OPD_BREDI	1PSC	TIM beta/alpha-barrel	SupFam	Phosphotriesterase	7794910	Two distinct structural folds
	**3.1.8.1**	Aryldialkylphosphatase	Q15165	PON2_HUMAN	1V04	6-bladed beta-propeller	SupFam	Arylesterase	8661009, 11579088, 15098021	
69	**3.1.8.2**	Diisopropyl-fluorophosphatase	Q7SIG4	DFPA_LOLVU	2GVV	6-bladed beta-propeller	SupFam	SMP-30/Gluconolaconase/LRE-like	11295437, 11171055	Two distinct structural folds
	**3.1.8.2**	Diisopropyl-fluorophosphatase	P77814	PEPQ_PSEHA	similar to 2BWX	Creatinase/aminopeptidase	SupFam	Metallopeptidase family M24	9079288	
70	**3.1.11.2**	Exodeoxyribonuclease III	P09030	EX3_ECOLI	1AKO	DNase I-like	SupFam	Endonuclease/Exonuclease/phosphatase	7885481, 8948651	Two distinct structural folds
	**3.1.11.2**	Exodeoxyribonuclease III	Q9BQ50	TREX2_HUMAN	1Y97	Ribonuclease H-like motif	SupFam	Exonuclease	15661738, 10391904	
	**3.1.11.2**	Exodeoxyribonuclease III	O60671	RAD1_HUMAN	3A1J	DNA clamp	SupFam	Repair protein Rad1/Rec1/Rad17	9660799, 9716408	
71	**3.1.11.3**	Exodeoxyribonuclease (lambda-induced)	P06229	EXO5_BPT5	1UT5	PIN_domain-like (catalytic domain)	SupFam	5'3'-exonuclease, N-terminal resolvase-like domain	3002857, 9874768	Two distinct structural folds
	**3.1.11.3**	Exodeoxyribonuclease (lambda-induced)	P03697	EXO_LAMBD	1AVQ	Restriction endonuclease-like	SupFam	YqaJ viral recombinase family	9295273	
72	**3.1.13.1**	Exoribonuclease II	P30850	RNB_ECOLI	2IX0	OB-fold	SupFam	Ribonuclease II (RNB)	8497196	Two distinct structural

	**3.1.13.1**	Exoribonuclease II	Q96AZ6	ISG20_HUMAN	1WLJ	Ribonuclease H-like motif	SupFam	Exonuclease	11401564, 12594219	folds
73	**3.1.21.1**	Deoxyribonuclease I	P00639	DNAS1_BOVIN	1DNK	Ribonuclease H-like motif and DNase I-like	SupFam	Endonuclease/ Exonuclease/phosphatase family	9469931	Two distinct structural folds
	**3.1.21.1**	Deoxyribonuclease I	P25736	END1_ECOLI	1OUP	His-Me finger endonucleases	SupFam	Endonuclease I	7867949	
74	**3.1.21.2**	Deoxyribonuclease IV (phage-T4-induced)	P0A6C1	END4_ECOLI	1QUM	TIM beta/alpha-barrel	SupFam	Xylose isomerase-like TIM barrel (clan)	10458614	Two distinct structural folds
	**3.1.21.2**	Deoxyribonuclease IV (phage-T4-induced)	P00641	ENRN_BPT7	1M0D	Restriction endonuclease-like	SupFam	Phage endonuclease I	12093751	
75	**3.1.22.4**	Crossover junction endodeoxyribonuclease	P0A814	RUVC_ECOLI	1HJR	Ribonuclease H-like motif	SupFam	RuvC	7923356, 1661673	Two distinct structural folds
	**3.1.22.4**	Crossover junction endodeoxyribonuclease	P0AG74	RUSA_ECOLI	1Q8R	Bacillus chorismate mutase-like	SupFam	RusA	14656440, 9169457	
76	**3.1.26.4**	Calf thymus ribonuclease H	P10442	RNH2_ECOLI	2ETJ	Ribonuclease H-like motif	SupFam	RNase HII	2172991	Two distinct structural folds
	**3.1.26.4**	Calf thymus ribonuclease H	P13319	RNH_BPT4	1TFR	PIN_domain-like (catalytic domain)	SupFam	T4 RNase H, C terminal	1703156, 8674116	
77	**3.2.1.1**	Alpha-amylase	P25718	AMY1_ECOLI	similar to 1EA9	TIM beta/alpha-barrel and Glycosyl hydrolase domain	SupFam	Alpha-amylase	9268356	Two distinct structural folds
	**3.2.1.1**	Alpha-amylase	P49067	AMYA_PYRFU	similar to 1K1X	7-stranded beta/alpha barrel	SupFam	Glycosyl hydrolase family 57	8226990	
78	**3.2.1.4**	Cellulase	P10476	GUNA_PSEFL	N-term is similar to 1UT9	Alpha/alpha toroid	SupFam	Glycosyl hydrolase 9 family (cellulase E)	2851699	At least four distinct structural folds
	**3.2.1.4**	Cellulase	P17901	GUNA_CLOCE	1EDG	TIM beta/alpha-barrel	SupFam	Beta-glycanases	1744052, 8535787	
	**3.2.1.4**	Cellulase	P33682	GUN1_STRHA	2BOD	7-stranded beta/alpha barrel	SupFam	Glycosyl hydrolases family 6	1400190	
	**3.2.1.4**	Cellulase	P55742	GUNM_CLOTM	2FVG	Phosphorylase/hydrolase-like	SupFam	Peptidase M42	GenBank: 1097207	
	**3.2.1.4**	Cellulase	P22669	GUN_ASPAC	1KS4	Concanavalin A-like lectins/ glucanases	SupFam	Glycosyl hydrolase 11 family (cellulase G)	2249253, 7586029, 2379837	
	**3.2.1.4**	Cellulase	Q05622	GUNE_RUMFL	similar to 1L0H	Predicted: Acyl carrier protein-like	SupFam	Pfam hit	8360615	

79	**3.2.1.8**	Endo-1,4-ß-xylanase	P23551	XYNA_BUTFI	1XAS	TIM beta/alpha-barrel	SupFam	Beta-glycanases	2198249, 1909424	Two distinct structural folds
	**3.2.1.8**	Endo-1,4-ß-xylanase	P33558	XYNA2_CLOSR	1QH6	Concanavalin A-like lectins/ glucanases	SupFam	Glycosyl hydrolase 11 family (cellulase G)	7763496	
80	**3.2.1.14**	Chitinase	P07254	CHIA_SERMA	1RD6	TIM beta/alpha-barrel (catalytic domain)	SupFam	Type II chitinase	16453672	Two distinct structural folds
	**3.2.1.14**	Chitinase	P24626	CHI1_ORYSA	2BAA	Lysozyme-like	SupFam	Family 19 glycosidase	1893114	
81	**3.2.1.17**	Lysozyme	P34020	LYS_CLOAB	N-term is similar to 1JFX	TIM beta/alpha-barrel	SupFam	1,4-beta-N-acetylmuraminidase	1599233, 7649184	At least two distinct structural folds
	**3.2.1.17**	Lysozyme	Q37875	LYS_BPP1	1XJT	Lysozyme-like	SupFam	Phage lysozyme	8576044, 9514719	
	**3.2.1.17**	Lysozyme	P10773	LYB_BACSU	-	-	SupFam	Pfam hit	3148618	
82	**3.2.1.20**	Alpha-glucosidase	Q02751	MALT_CANAL	1JI1	TIM beta/alpha-barrel and Glycosyl hydrolase domain	SupFam	Glycosyl hydrolase family 13	1400249	Two distinct structural folds
	**3.2.1.20**	Alpha-glucosidase	O33830	AGLA_THEMA	1OBB	Rossmann-fold and LDH C-terminal domain-like	SupFam	Glycosyl hydrolase family 4	12062450, 10972187	
83	**3.2.1.22**	Alpha-galactosidase	P27756	AGAL_STRMU	1R46	TIM beta/alpha-barrel	SupFam	Glycosyl hydrolase family 36	1649890	Two distinct structural folds
	**3.2.1.22**	Alpha-galactosidase	P06720	AGAL_ECOLI	similar to 1OBB	Rossmann-fold and LDH C-terminal domain-like	SupFam	Glycosyl hydrolase family 4	2831880	
84	**3.2.1.35**	Hyaluronoglucosaminidase	Q12891	HYAL2_HUMAN	1FCQ	TIM beta/alpha-barrel	SupFam	Glycosyl hydrolase family 56	9712871, 17503783	Two distinct structural folds
	**3.2.1.35**	Hyaluronoglucosaminidase	Q54699	HYLP2_BPH45	2PK1	Triple-stranded beta-helix	SupFam	Hyaluronidase_1	7622224	
85	**3.2.1.37**	Xylan 1,4-ß-xylosidase	P07129	XYNB_BACPU	1YIF	5-bladed beta-propeller	SupFam	Glycosyl hydrolase family 43	2440680, 7766665	At least two distinct structural folds
	**3.2.1.37**	Xylan 1,4-ß-xylosidase	P36906	XYNB_THESA	1PX8	TIM beta/alpha-barrel	SupFam	Glycosyl hydrolase family 39	8612648	
	**3.2.1.37**	Xylan 1,4-ß-xylosidase	P45702	XYLA1_BACST	-	-	SupFam	Glycosyl hydrolase family 52	8074507, 11322943	
86	**3.2.1.39**	Glucan endo-1,3-ß-D-glucosidase	P33157	E13A_ARATH	2CYG	TIM beta/alpha-barrel	SupFam	Glycosyl hydrolase family 17	11405630, 16421930	At least two distinct structural folds
	**3.2.1.39**	Glucan endo-1,3-ß-D-glucosidase	P23903	E13B_BACCI	C-term is 2VY0	Predicted: Concanavalin A-like lectins/glucanases	SupFam	Glycosyl hydrolase family 16	2311931, 19154353	
	**3.2.1.39**	Glucan endo-1,3-ß-D-glucosidase	P22222	E13B_CELCE	-	-	SupFam	Glycosyl hydrolase family 64	1985933	

87	**3.2.1.49**	Alpha-N-acetylgalactosaminidase	P17050	NAGAB_HUMAN	3H53	TIM beta/alpha-barrel and Glycosyl hydrolase domain	SupFam	Melibiase	19683538	Two distinct structural folds
	**3.2.1.49**	Alpha-N-acetylgalactosaminidase	A4Q8F7	GH109_FLAME	2IXA	Rossmann-fold	SupFam	Oxidoreductase family	17401360	
88	**3.2.1.55**	Alpha-N-arabinofuranosidase	P53627	ABFA_STRLI	2C7F	TIM beta/alpha-barrel	SupFam	Glycosyl hydrolase family 51	8092996, 16336192	Two distinct structural folds
	**3.2.1.55**	Alpha-N-arabinofuranosidase	P45982	XYLB_BUTFI	1YRZ	5-bladed beta-propeller and Concanavalin A-like lectins/ glucanases	SupFam	Glycosyl hydrolase family 43	1905520, 18980579	
89	**3.2.1.73**	Licheninase	P07979	GUB_NICPL	2CYG	TIM beta/alpha-barrel	SupFam	Glycosyl hydrolase family 17	9452466	Two distinct structural folds
	**3.2.1.73**	Licheninase	P04957	GUB_BACSU	1GBG	Concanavalin A-like lectins/ glucanases	SupFam	Glycosyl hydrolase family 16	1740123	
	**3.2.1.73**	Licheninase	P19254	GUB_BACCI	1V5C	Alpha/alpha toroid	SupFam	Glycosyl hydrolase family 8 (cellulase D)	2377467	
90	**3.2.1.86**	6-phospho-ß-glucosidase	P11988	BGLB_ECOLI	1QOX	TIM beta/alpha-barrel	SupFam	Glycosyl hydrolase family 1	3034860	Two distinct structural folds
	**3.2.1.86**	6-phospho-ß-glucosidase	P17411	CHBF_ECOLI	1S6Y	Rossmann-fold and LDH C-terminal domain-like	SupFam	Glycosyl hydrolase family 4	10572139	
91	**3.2.1.91**	Cellulose 1,4-ß-cellobiosidase	P0C2S1	CELK_CLOTM	1UT9	Alpha/alpha toroid	SupFam	Glycosyl hydrolase 9 (cellulase E) family	10464199	Two distinct structural folds
	**3.2.1.91**	Cellulose 1,4-ß-cellobiosidase	P50401	GUXA_CELFI	similar to 1BVW	7-stranded beta/alpha barrel	SupFam	Glycosyl hydrolase 6 (cellulase B) family	8065260	
	**3.2.1.91**	Cellulose 1,4-ß-cellobiosidase	P07986	GUX_CELFI	1FH7	TIM beta/alpha-barrel	SupFam	Glycosyl hydrolase 10 (cellulase F) family	9537990	
92	**3.2.2.21**	DNA-3-methyladenine glycosylase II	P04395	3MG2_ECOLI	1MPG	DNA-glycosylase and TBP-like	SupFam	AlkA N-terminal domain and HhH-GPD	6389535	Two distinct structural folds
	**3.2.2.21**	DNA-3-methyladenine glycosylase II	P29372	3MG_HUMAN	1F4R (C-term)	FMT C-terminal domain-like	SupFam	Methylpurine-DNA glycosylase	8589517, 1645538	
93	**3.5.1.1**	Asparaginase	P38986	ASPG1_YEAST	1HFW	Glutaminase/Asparaginase	SupFam	Glutaminase/ Asparaginase	8026756	At least two distinct structural folds
	**3.5.1.1**	Asparaginase	P20933	ASPG_HUMAN	1APY	Ntn hydrolase-like	SupFam	(Glycosyl)asparaginase	1840528	
	**3.5.1.1**	Asparaginase	Q9RFN5	Q9RFN5_RHIET	-	-	SupFam	L-asparaginase II	10930734	
94	**3.5.1.4**	Amidase	P27765	AMID_PSECL	1OCK	Amidase signature	SupFam	Amidase	2013568	Two distinct structural folds
	**3.5.1.4**	Amidase	P11436	ALAM_PSEAE	2UXY	Carbon-nitrogen hydrolase	SupFam	Carbon-nitrogen hydrolase	87219101	

95	**3.5.1.9**	Arylformamidase	Q8K4H1	AFMID_MOUSE	2PBL	Alpha/beta hydrolase	SupFam	Pfam hit	12007602, 15935693	Two distinct structural folds
	**3.5.1.9**	Arylformamidase	P0C8P4	KYNB_RALME	similar to 1R61	The "swivelling" beta/beta/alpha domain	SupFam	Putative cyclase	14592712, 14592712	
96	**3.5.1.49**	Formamidase	Q50228	FMDA_METME	similar to 2F4L	CUB-like	SupFam	Acetamidase/formamidase	8841393	Two distinct structural folds
	**3.5.1.49**	Formamidase	O25836	AMIF_HELPY	2DYU	Carbon-nitrogen hydrolase	SupFam	Carbon-nitrogen hydrolase	11359566, 17307742, 14742519	
97	**3.5.1.52**	Peptide-N4-(N-acetyl-ß-glucosaminyl)asparagine amidase	Q02890	PNG1_YEAST	1X3W	Cysteine proteinases	SupFam	Transglutaminase-like	10831608, 11259433, 14726951, 15964983	At least two distinct structural folds
	**3.5.1.52**	Peptide-N4-(N-acetyl-ß-glucosaminyl)asparagine amidase	P21163	PNGF_ELIMR	1PNG	Nucleoplasmin-like/VP	SupFam	Peptide-N-glycosidase F	7918386, 7493989	
	**3.5.1.52**	Peptide-N4-(N-acetyl-ß-glucosaminyl)asparagine amidase	P81898	PNAA_PRUDU	-	-	SupFam	Patent US 5710016 (GenBank AAC20834)	9523720	
98	**3.5.2.6**	ß-lactamase	P00811	AMPC_ECOLI	2BLS	Beta-lactamase/ transpeptidase-like	SupFam	Beta-Lactamase/D-ala carboxypeptidase	6795623	Two distinct structural folds
	**3.5.2.6**	ß-lactamase	P14488	BLAB_BACCE	1BC2	Metallo-hydrolase/ oxidoreductase	SupFam	Zn metallo-beta-lactamase	9730812	
99	**3.5.2.17**	Hydroxyisourate hydrolase	O32142	HIUH_BACSU	similar to 1OO2	Prealbumin-like	SupFam	HIUase/Transthyretin	16098976, 16782815	Two distinct structural folds
	**3.5.2.17**	Hydroxyisourate hydrolase	Q8S3J3	HIUH_SOYBN	similar to 1cbg	TIM beta/alpha-barrel	SupFam	Glycosyl hydrolase family 1	10567345, 12481089	
100	**3.5.4.1**	Cytosine deaminase	P25524	CODA_ECOLI	1R9Z	TIM beta/alpha-barrel (catalytic domain)	SupFam	Amidohydrolase	11812140, 1640834	Two distinct structural folds
	**3.5.4.1**	Cytosine deaminase	Q12178	FCY1_YEAST	1P6O	Cytidine deaminase-like	SupFam	Cytidine and deoxycytidylate deaminase	9000374	
101	**3.5.4.3**	Guanine deaminase	P76641	GUAD_ECOLI	2UZ9	TIM beta/alpha-barrel (catalytic domain)	SupFam	Amidohydrolase	10913105	Two distinct structural folds
	**3.5.4.3**	Guanine deaminase	O34598	GUAD_BACSU	1WKQ	Cytidine deaminase-like	SupFam	Cytidine and deoxycytidylate deaminase	15159585, 15180998, 11101664	

102	**3.5.4.9**	Methenyltetrahydrofolate cyclohydrolase	P24186	FOLD_ECOLI	1B0A	Aminoacid dehydrogenase-like N-terminal domain and Rossmann-fold	SupFam	Tetrahydrofolate dehydrogenase/ cyclohydrolase	1748668, 10386884	Two distinct structural folds
	**3.5.4.9**	Methenyltetrahydrofolate cyclohydrolase	Q49135	FCHA_METEA	similar to 1O5H	Methenyltetrahydrofolate cyclohydrolase-like	SupFam	Formiminotransferase-cyclodeaminase	10215859	
103	**3.5.4.10**	IMP cyclohydrolase	P31939	PUR9_HUMAN	1PKX	Methylglyoxal synthase-like (IMP cyclohydrolase domain)	SupFam	AICARFT/IMPCHase	8567683, 14756553, 14966129	Two distinct structural folds
	**3.5.4.10**	IMP cyclohydrolase	A0B9N7	PURO_METTP	2NTK	Ntn hydrolase-like	SupFam	IMP cyclohydrolase-like (archaeal)	17407260, 12012346	
104	**3.5.99.6**	Glucosamine-6-phosphate deaminase	P0A759	NAGB_ECOLI	1FS5	NagB/RpiA/CoA transferase-like	SupFam	Glucosamine_iso	1734962, 8747459	Two distinct structural folds
	**3.5.99.6**	Glucosamine-6-phosphate deaminase	Q5JDU3	Q5JDU3_PYRKO	2CB0	SIS domain	SupFam	SIS domain	16199574, 16857666, 17387737	
105	**3.6.1.1**	Inorganic diphosphatase	P21216	IPYR_ARATH	1TWL	OB-fold	SupFam	Pyrophosphatase	654155	At least three distinct structural folds
	**3.6.1.1**	Inorganic diphosphatase	P31414	AVP1_ARATH	-	Integral membrane protein	SupFam	Inorganic H^+ pyrophosphatase	9268385	
	**3.6.1.1**	Inorganic diphosphatase	P37487	PPAC_BACSU	1WPM	DHH phosphoesterases	SupFam	Mn-dependent inorganic pyrophosphatase	9845334, 11697905	
	**3.6.1.1**	Inorganic diphosphatase	Q9JMQ2	PPAX_BACSU	similar to 2HDO	Predicted: HAD-like	SupFam	HAD-like hydrolase	12359880	
106	**3.6.1.5**	Apyrase	O96559	APY_CIMLE	2H2N	5-bladed beta-propeller	SupFam	Apyrase	9804829, 15006348	At least two distinct structural folds
	**3.6.1.5**	Apyrase	P50635	APY_AEDAE	similar to 2Z1A	5-nucleotidase and Metallo-dependent phosphatases	SupFam	5'-nucleotidase	2173922	
	**3.6.1.5**	Apyrase	O75355	ENTP3_HUMAN	3CJ1, similar to 1T6C	Predicted: Ribonuclease H-like motif	SupFam	Actin-like ATPase domain	9675246, 8955160	
107	**3.6.1.11**	Exopolyphosphatase	P29014	PPX_ECOLI	1U6Z	Ribonuclease H-like motif and HD-domain/PDEase-like	SupFam	Ppx associated domain	8212131	Two distinct structural folds
	**3.6.1.11**	Exopolyphosphatase	P38698	PPX1_YEAST	2QB6, similar to 1K20	DHH phosphoesterases	SupFam	Mn-dependent inorganic pyrophosphatase	7860598	
108	**3.6.1.13**	ADP-ribose diphosphatase	O94759	TRPM2_HUMAN	similar to 1QVJ	NUDIX	SupFam	NUDIX domain (paper)	11385575	Two distinct structural

	**3.6.1.13**	ADP-ribose diphosphatase	Q5M886	ADPRM_RAT	2NXF	Metallo-dependent phosphatases	SupFam	Calcineurin-like phosphoesterase	18352857	folds
109	**3.6.1.17**	Bis(5'-nucleosyl)-tetraphosphatase (asymmetrical)	P49776	APH1_SCHPO	1FHI	HIT-like	SupFam	HIT family	8554540	At least two distinct structural folds
	**3.6.1.17**	Bis(5'-nucleosyl)-tetraphosphatase (asymmetrical)	P50583	AP4A_HUMAN	1XSA	NUDIX	SupFam	MutT-like	7487923	
	**3.6.1.17**	Bis(5'-nucleosyl)-tetraphosphatase (asymmetrical)	O31614	PRPE_BACSU	similar to 1G5B	Predicted: Metallo-dependent phosphatases	SupFam	Protein serine/threonine phosphatase	12059787	
110	**3.6.1.23**	DUTP diphosphatase	P33316	DUT_HUMAN	1Q5H	Beta-clip	SupFam	dUTPase	8805593, 8631816	Two distinct structural folds
	**3.6.1.23**	DUTP diphosphatase	O15923	O15923_TRYCR (DUT)	1OGK	all-alpha NTP pyrophosphatases	SupFam	dUTPase_2	14725764, 12208522	
111	**3.6.1.45**	UDP-sugar diphosphatase	P07024	USHA_ECOLI	2USH	Metallo-dependent phosphatases and 5'-nucleotidase (syn. UDP-sugar hydrolase), C-terminal domain	SupFam	5'-nucleotidase	11491293, 10331872	Two distinct structural folds
	**3.6.1.45**	UDP-sugar diphosphatase	O95848	NUD14_HUMAN	similar to 1VIU	NUDIX	SupFam	NUDIX hydrolase	12429023	
112	**3.8.1.3**	Haloacetate dehalogenase	Q01398	DEH1_MORSB	1G5F	Alpha/beta hydrolase	SupFam	Haloalkane dehalogenase; pdb and 1IZ7, pdb and 1MJ5	1512562, 11939779	Two distinct structural folds
	**3.8.1.3**	Haloacetate dehalogenase	Q01399	DEH2_MORSB	1JUD	HAD-like	SupFam	HAD-related	8702766	
113	**4.1.1.17**	Ornithine decarboxylase	P24169	DCOS_ECOLI	1ORD	PLP-dependent transferase-like and Ornithine decarboxylase C-terminal domain	SupFam	Orn/Lys/Arg decarboxylase, class I	7563080	Two distinct structural folds
	**4.1.1.17**	Ornithine decarboxylase	P11926	DCOR_HUMAN	1D7K	TIM beta/alpha-barrel	SupFam	Orn/Lys/Arg decarboxylase, class II	2587220, 2318872	
114	**4.1.1.18**	Lysine decarboxylase	P0A9H3	LDCI_ECOLI	similar to 1ORD	PLP-dependent transferase-like and Ornithine decarboxylase C-terminal domain	SupFam	Orn/Lys/Arg decarboxylase class-I	4204273	Two distinct structural folds
	**4.1.1.18**	Lysine decarboxylase	O50657	DCLO_SELRU	similar to 7ODC	TIM beta/alpha-barrel	SupFam	Orn/Lys/Arg decarboxylase class-II	11073919	

115	**4.1.1.19**	Arginine decarboxylase	P28629	ADIA_ECOLI	similar to 1C4K	PLP-dependent transferase-like and Ornithine decarboxylase C-terminal domain	SupFam	Orn/Lys/Arg decarboxylase, class I	4204273	At least two distinct structural folds
	**4.1.1.19**	Arginine decarboxylase	P21170	SPEA_ECOLI	similar to 1TWI	Predicted: TIM beta/alpha barrel	SupFam	Orn/Lys/Arg decarboxylase class II	8383109, 19298070	
	**4.1.1.19**	Arginine decarboxylase	Q57764	PDAD_METJA	1N2M	Pyruvoyl-dependent histidine and arginine decarboxylases	SupFam	Pyruvoyl-dependent ArgDC	11980912	
116	**4.2.1.1**	Carbonate dehydratase	P17582	CYNT_ECOLI	similar to 1EKJ	Resolvase-like	SupFam	Beta-carbonic anhydrase	1740425	Two distinct structural folds
	**4.2.1.1**	Carbonate dehydratase	P00915	CAH1_HUMAN	2FOY	Carbonic anhydrase	SupFam	Eukaryotic-type carbonic anhydrase	804171, 4207120	
	**4.2.1.1**	Carbonate dehydratase	P40881	CAH_METTE	1QQ0	Single-stranded left-handed beta-helix	SupFam	Gamma-carbonic anhydrase-like	8041719	
117	**4.2.1.2**	Fumarate hydratase	P05042	FUMC_ECOLI	1FUO	L-aspartase-like	SupFam	L-aspartase/fumarase	8909293, 3282546	Two distinct structural folds
	**4.2.1.2**	Fumarate hydratase	P00923	FUMA_ECOLI	C-term domain is 2ISB	The "swivelling" beta/beta/alpha domain	SupFam	Class I fumarase	1917897	
118	**4.2.1.10**	3-dehydroquinate dehydratase	P43877	AROQ_ACTPL	1UQR	Flavodoxin-like	SupFam	Type II 3-dehydroquinate dehydratase	14993670, 8170389, 2848727	Two distinct structural folds
	**4.2.1.10**	3-dehydroquinate dehydratase	P05194	AROD_ECOLI	1QFE	TIM beta/alpha-barrel	SupFam	Class I aldolase	3541912	
119	**4.2.1.17**	Enoyl-CoA hydratase	P21177	FADB_ECOLI	1WDK	ClpP/crotonase, Rossmann-fold and Thiolase-like	SupFam	Enoyl-CoA hydratase/isomerase family	3286611	Two distinct structural folds
	**4.2.1.17**	Enoyl-CoA hydratase	P96807	ECH1_MYCTU	2C2I	Thioesterase/thiol ester dehydrase-isomerase	SupFam	MaoC dehydratase	16963641	
120	**4.2.1.78**	Norcoclaurine synthase	A2A1A0	NCS1_COPJA	1GP4	Double-stranded beta-helix	SupFam	2OG-Fe(II) oxygenase superfamily	17204481	Two distinct structural folds
	**4.2.1.78**	Norcoclaurine synthase	Q67A25	NCS_THLFG, NCS2_COPJA	2VNE, similar to 1FM4	TBP-like	SupFam	Pathogenesis-related protein	17696451, 18391427	
121	**4.2.1.92**	Hydroperoxide dehydratase	Q96242	CP74A_ARATH	2RCH	Cytochrome P450	SupFam	Cytochrome P450	8756596, 10420644	Two distinct structural folds
	**4.2.1.92**	Hydroperoxide dehydratase	O16025	AOSL_PLEHO	1U5U	Heme-dependent catalase-like	SupFam	Lipoxygenase	10559269, 9302294	

122	**4.2.2.2**	Pectate lyase	P0C1C2	PEL3_ERWCA	1PLU	Single-stranded right-handed beta-helix	SupFam	Pectate lyase C	12226275	Two distinct structural folds
	**4.2.2.2**	Pectate lyase	P14005	PELP_PECCC	similar to 1NXC	Alpha/alpha toroid	SupFam	Periplasmic pectate lyase	2695748, 17881361	
123	**4.2.99.18**	DNA-(apurinic or apyrimidinic site) lyase	P20625	END3_ECOLI	2ABK	DNA-glycosylase	SupFam	Endonuclease III	2669955	Four distinct structural folds
	**4.2.99.18**	DNA-(apurinic or apyrimidinic site) lyase	P27695	APEX1_HUMAN	1E9N	DNase I-like	SupFam	DNase I-like	1383925, 1380694	
	**4.2.99.18**	DNA-(apurinic or apyrimidinic site) lyase	P22936	APN1_YEAST	similar to 1QTW	TIM beta/alpha-barrel	SupFam	Endonuclease IV	3056935	
	**4.2.99.18**	DNA-(apurinic or apyrimidinic site) lyase	P05523	FPG_ECOLI	1K82	MutM-like DNA repair protein	SupFam	Middle domain of MutM-like DNA repair proteins	11106507, 14607836, 11912217	
124	**4.3.1.17**	L-serine ammonia-lyase (was 4.2.1.13)	P16095	SDHL_ECOLI	2IQQ (N-term)	FwdE/GAPDH domain-like	SupFam	Fe-S dependent L-serine dehydratase	2504697, 8436113, 8385012	Two distinct structural folds
	**4.3.1.17**	L-serine ammonia-lyase (was 4.2.1.13)	P20132	SDHL_HUMAN	1P5J	Tryptophan synthase beta subunit-like PLP-dependent enzymes	SupFam	Serine/threonine dehydratase	14596599, 14646100, 2674117	
125	**4.6.1.1**	Adenylate cyclase	Q08828	CYA1_HUMAN	1CS4	P-loop containing NTPases and Ferredoxin-like	SupFam	Adenylate/guanylate cyclase (class III AC)	8314585	At least three distinct structural folds
	**4.6.1.1**	Adenylate cyclase	O69199	O69199_AERHY	2ACA	CYTH-like phosphatases	SupFam	CYTH domain	9642185, 16905149	
	**4.6.1.1**	Adenylate cyclase	P15318	CYAA_BORPE	N-term is 1YRU	EF Hand-like	SupFam	Anthrax toxin LF (class II AC)	2897067, 16138079	
	**4.6.1.1**	Adenylate cyclase	P00936	CYAA_ECOLI	-	-	SupFam	Adenylate_cyclase class I	6344011, 92011391	
126	**5.1.1.1**	Alanine racemase	P0A6B4	ALR1_ECOLI	2RJG	Domain of alpha and beta subunits of F1 ATP synthase-like and TIM beta/alpha-barrel	SupFam	Alanine racemase	18434499	Two distinct structural folds
	**5.1.1.1**	Alanine racemase	Q9UW18	TOXG_COCCA	similar to 1M6S	PLP-dependent transferase-like	SupFam	Beta-eliminating lyase	10671527	
127	**5.1.1.13**	Aspartate racemase	P83989	Q2L695_ANABR (RACD_ANABR)	similar to 1WTC	Tryptophan synthase beta subunit-like PLP-dependent enzymes	SupFam	Pyridoxal-phosphate dependent enzyme	12568809, 16452311	Two distinct structural folds
	**5.1.1.13**	Aspartate racemase	O58403	O58403_PYRHO	1JFL	ATC-like	SupFam	Asp/Glu/Hydantoin racemase	12051922	

128	**5.2.1.8**	Peptidylprolyl isomerase	P0AFL3	PPIA_ECOLI	1CLH	Cyclophilin-like	SupFam	Cyclophilin	9501079, 17909185	Two distinct structural folds
	**5.2.1.8**	Peptidylprolyl isomerase	P45523	FKBA_ECOLI	1Q6H	FKBP-like	SupFam	FKBP-type PPIase	1379319	
129	**5.3.1.6**	Ribose-5-phosphate isomerase	P0A7Z0	RPIA_ECOLI	1LKZ	NagB/RpiA/CoA transferase-like and Ferredoxin-like	SupFam	Ribose 5-phosphate isomerase	12211039, 8366047	Two distinct structural folds
	**5.3.1.6**	Ribose-5-phosphate isomerase	P37351	RPIB_ECOLI	1NN4	Ribose/Galactose isomerase RpiB/AlsB	SupFam	Ribose/Galactose Isomerase	14499611, 8576032	
130	**5.3.1.8**	Mannose-6-phosphate isomerase	Q59935	MANA_STRMU	1QWR	Double-stranded beta-helix	SupFam	RmlC-like cupins	8293960	At least two distinct structural folds
	**5.3.1.8**	Mannose-6-phosphate isomerase	Q4JCA7	PGMI_SULAC	similar to 1TZB	SIS domain	SupFam	SIS_domain	14551194	
	**5.3.1.8**	Mannose-6-phosphate isomerase	P29954	MANA_RHIME	similar to 2GZ6	Predicted: Alpha/alpha toroid	SupFam	GlcNAc 2-epimerase	1452036	
131	**5.3.1.9**	Glucose-6-phosphate isomerase	P06744	G6PI_HUMAN	1JLH	SIS domain	SupFam	Phosphoglucose isomerase	12573240	Two distinct structural folds
	**5.3.1.9**	Glucose-6-phosphate isomerase	P84140	G6PI_THELI	1J3P	Double-stranded beta-helix	SupFam	Glucose-6-phosphate isomerase (GPI) (Archaeal-type GPI)	12560104	
132	**5.3.3.2**	Isopentenyl-diphosphate delta-isomerase	P50740	IDI2_BACSU	1P0K	TIM beta/alpha-barrel	SupFam	FMN-dependent dehydrogenase	15206931, 12798687	Two distinct structural folds
	**5.3.3.2**	Isopentenyl-diphosphate delta-isomerase	Q46822	IDI_ECOLI	1HZT	NUDIX	SupFam	NUDIX	10419945, 11285217	
133	**5.3.3.10**	5-carboxymethyl-2-hydroxymuconate delta-isomerase	Q05354	HPCD_ECOLX	1OTG	Tautomerase/MIF	SupFam	CHMI, 5-carboxymethyl-2-hydroxymuconate isomerase	8547259, 2194841	Two distinct structural folds
	**5.3.3.10**	5-carboxymethyl-2-hydroxymuconate delta-isomerase	P37352	HPCE_ECOLX	1I7O	FAH	SupFam	Fumarylacetoacetate (FAA) hydrolase	8223600	
134	**5.3.99.2**	Prostaglandin-D synthase	O60760	PTGD2_HUMAN	1IYH	Thioredoxin fold and GST C-terminal domain-like	SupFam	Glutathione S-transferase	9353279, 10824118	Two distinct structural folds
	**5.3.99.2**	Prostaglandin-D synthase	P41222	PTGDS_HUMAN	2CZT	Lipocalins	SupFam	Lipocalin family	8336134, 9475419	
135	**5.3.99.3**	Prostaglandin-E synthase	Q9N0A4	PGES2_MACFA	1Z9H	Thioredoxin fold and GST C-terminal domain-like	SupFam	Glutathione S-transferase	15854652, 11866447	Two distinct structural folds
	**5.3.99.3**	Prostaglandin-E synthase	O14684	PTGES_HUMAN	2H8A	MAPEG domain-like	SupFam	MAPEG family	12672824, 12460774	

No.	EC no.	Enzyme name	NCBI	UniProt	PDB	SCOP fold	Super-family	Family (Pfam)	Ref.	Predicted
	**5.3.99.3**	Prostaglandin-E synthase	Q15185	TEBP_HUMAN	1EJF	HSP20-like chaperones	SupFam	CS domain	10922363, 10811660	
136	**5.4.2.1**	Phosphoglycerate mutase	P31217	GPMA_ECOLI	1E59	Phosphoglycerate mutase-like	SupFam	Phosphoglycerate mutase	11038361	Two distinct structural folds
	**5.4.2.1**	Phosphoglycerate mutase	P52832	GPMI_PSESM	1EJJ	Alkaline phosphatase-like	SupFam	2,3-Bisphosphoglycerate-independent phosphoglycerate mutase	12076796	
137	**5.4.2.8**	Phosphomannomutase	P26405	RFBK_SALTY	1K2Y	Phosphoglucomutase	SupFam	Phosphoglucomutase	1710759	Two distinct structural folds
	**5.4.2.8**	Phosphomannomutase	Q92871	PMM1_HUMAN	2FUC	HAD-like	SupFam	Eukaryotic phosphomannomutase	9119384	
138	**5.4.99.5**	Chorismate mutase	P42517	CHMU_ENTAG	2AO2	Chorismate mutase II	SupFam	Secreted chorismate mutase-like	8335631, 9497350, 7496534	Two distinct structural folds
	**5.4.99.5**	Chorismate mutase	P19080	CHMU_BACSU	1COM	Bacillus chorismate mutase-like	SupFam	Chorismate mutase	8378335	
139	**5.5.1.1**	Muconate cycloisomerase	P08310	CATB_PSEPU	1MUC	TIM beta/alpha-barrel and Enolase N-terminal domain-like	SupFam	D-glucarate dehydratase-like	3609743, 3612800	Two distinct structural folds
	**5.5.1.1**	Muconate cycloisomerase	P46057	MLE_TRICU	C-term is similar to C-term of 1JOF	7-bladed beta-propeller	SupFam	WD40/YVTN repeat	8110801, 11937053	
140	**5.99.1.2**	DNA topoisomerase	P06612	TOP1_ECOLI	1CY1	Prokaryotic type I DNA topoisomerase	SupFam	Prokaryotic type I DNA topoisomerase	3029379, 8114910, 7779808	Two distinct structural folds
	**5.99.1.2**	DNA topoisomerase	P11387	TOP1_HUMAN	1LPQ	DNA breaking-rejoining enzymes	SupFam	Eukaryotic DNA topoisomerase I	98155246, 8747458, 16352556	
141	**6.1.1.6**	Lysine—tRNA ligase	P13030	SYK1_ECOLI	1BBU	Class II aaRS and biotin synthetases	SupFam	Class II aminoacyl-tRNA synthetase	7735833, 11887185, 2183178	Two distinct structural folds
	**6.1.1.6**	Lysine—tRNA ligase	Q9YFT9	SYK_AERPE	1IRX	Adenine nucleotide alpha hydrolase-like	SupFam	Class I lysyl-tRNA synthetase	9353192, 11887185, 16529662	
**No.**	**EC no.**	**Enzyme name**	**NCBI**	**UniProt**	**PDB**	**SCOP fold**	**Super-family**	**Family (Pfam)**	**Ref.**	**Predicted**

142	**1.2.1.31**	L-aminoadipate-semialdehyde dehydrogenase	P49419	AL7A1_HUMAN	2J6L, similar to 1EUH	Predicted: ALDH-like	SupFam	Aldehyde dehydrogenase	16491085, 18694748	Predicted analogs
	**1.2.1.31**	L-aminoadipate-semialdehyde dehydrogenase	P07702	LYS2_YEAST	similar to 2VSQ	Predicted: CoA-dependent acyltransferases, Acetyl-CoA synthetase-like and Rossmann-fold	SupFam	NAD_binding_4	10320345, 12756539	Predicted analogs
143	**1.3.1.9**	Enoyl-[acyl-carrier-protein] reductase (NADH)	P0A5Y6	INHA_MYCTU	2NV6	Rossmann-fold	SupFam	Short-chain dehydrogenase	10336454	Predicted analogs
	**1.3.1.9**	Enoyl-[acyl-carrier-protein] reductase (NADH)	P07149	FAS1_YEAST	3HMJ	Predicted: TIM beta/alpha-barrel	SupFam	Enoyl-[acyl-carrier-protein] reductase (Fungal fatty acid synthetase subunit beta)	17431175, 19679086	Predicted analogs
144	**1.3.1.20**	Trans-1,2-dihydrobenzene-1,2-diol dehydrogenase	Q7JK39	DHDH_MACFU	2O48	Predicted: Rossmann-fold and FwdE/GAPDH domain-like	SupFam	Oxidoreductase family	11306093, 10477285, 17654552	Predicted analogs
	**1.3.1.20**	Trans-1,2-dihydrobenzene-1,2-diol dehydrogenase	Q04828	AK1C1_HUMAN	1MRQ	TIM beta/alpha-barrel	SupFam	Aldo/keto reductase	11013348, 8573067	Predicted analogs
145	**1.5.1.3**	Dihydrofolate reductase	P11045	DYR_BACSU	1MVS	Dihydrofolate reductase-like	SupFam	Dihydrofolate reductases	1731871	Predicted analogs
	**1.5.1.3**	Dihydrofolate reductase	P0AFS3	FOLM_ECOLI	similar to 3edm	Predicted: Rossmann-fold	SupFam	Short chain dehydrogenase	14617668	Predicted analogs
146	**1.14.13.39**	Nitric-oxide synthase	P29476	NOS1_RAT	1TLL	Reductase/isomerase/elongation factor common domain and Flavodoxin-like and Ferredoxin reductase-like C-terminal NADP-linked domain	SupFam	NOS family	1379068	Predicted analogs
	**1.14.13.39**	Nitric-oxide synthase	Q66GP9	NOS1_ARATH	similar to 3EC1	Predicted: P-loop	SupFam	Plant NOS family	14526079, 11916378	Predicted analogs
147	**2.1.1.17**	Phosphatidylethanolamine N-methyltransferase	Q05197	PMTA_RHOSH	similar to 3DH0	Predicted: SAM-dependent methyltransferases	SupFam	SAM-dependent methyltransferase	8340421	Predicted analogs
	**2.1.1.17**	Phosphatidylethanolamine N-methyltransferase	P05374	PEM1_YEAST	-	Integral membrane protein	SupFam	Pfam hit	2445736	Predicted analogs
148	**2.1.1.31**	tRNA (guanine-N1-)-methyltransferase	P0A873	TRMD_ECOLI	1P9P	Alpha/beta knot	SupFam	TRNA (Guanine-1)-methyltransferase	6337136, 14583191	Predicted analogs

	2.1.1.31	tRNA (guanine-N1-)-methyltransferase	Q32P41	TRMT5_HUMAN	C-term is similar to N-term of 2FRN	Predicted: S-adenosyl-L-methionine-dependent methyltransferases	SupFam	Met-10+ like-protein	15248782	Predicted analogs
149	2.3.1.1	Amino-acid N-acetyltransferase	P0A6C5	ARGA_ECOLI	2R8V	Acyl-CoA N-acyltransferases (Nat)	SupFam	Acetyltransferase (GNAT) family	16890	Predicted analogs
	2.3.1.1	Amino-acid N-acetyltransferase	Q04728	ARGJ_YEAST	1VZ6	DmpA/ArgJ-like	SupFam	ArgJ family	9428669	Predicted analogs
150	2.3.1.43	Phosphatidylcholine—sterol O-acyltransferase	P04180	LCAT_HUMAN	1ex9	Alpha/beta hydrolase	SupFam	Lecithin:cholesterol acyltransferase	2823898	Predicted analogs
	2.3.1.43	Phosphatidylcholine—sterol O-acyltransferase	P10480	GCAT_AERHY	similar to: 1PP4	Flavodoxin-like	SupFam	Lipase_GDSL	3280033	Predicted analogs
151	2.3.1.51	1-acylglycerol-3-phosphate O-acyltransferase	O15120	PLCB_HUMAN	similar to 1K30	Predicted: Glycerol-3-phosphate (1)-acyltransferase	SupFam	Acyltransferase	9242711	Predicted analogs
	2.3.1.51	1-acylglycerol-3-phosphate O-acyltransferase	Q12385	ICT1_YEAST	similar to 1IUN	Predicted: Alpha/beta hydrolase	SupFam	Alpha/beta hydrolase	18252723	Predicted analogs
152	2.3.1.84	Alcohol O-acetyltransferase	P40353	ATF1_YEAST	similar to 1L5A	Predicted: CoA-dependent acyltransferases	SupFam	Alcohol acetyltransferase	8085822, 7764365	Predicted analogs
	2.3.1.84	Alcohol O-acetyltransferase	P38295	MCFS2_YEAST	similar to 1VA4	Predicted: Alpha/beta hydrolase	SupFam	Abhydrolase_1	16361250	Predicted analogs
153	2.7.4.21	Inositol-hexakisphosphate kinase	Q12494	KCS1_YEAST	1W2D	SAICAR synthase-like	SupFam	Inositol phosphokinase	15350214	Predicted analogs
	2.7.4.21	Inositol-hexakisphosphate kinase	Q06685	VIP1_YEAST	similar to 1GSA	Predicted: ATP-grasp	SupFam	Histidine acid phosphatase	17412958, 17690096	Predicted analogs
154	3.1.1.1	Carboxylesterase	O33407	ESTA_PSEAE	similar to 1PP4	Predicted: Flavodoxin-like	SupFam	GDSL-like Lipase/ Acylhydrolase	10559163	Predicted analogs
	3.1.1.1	Carboxylesterase	P13001	BIOH_ECOLI	1M33	Alpha/beta hydrolase	SupFam	Alpha/beta hydrolase	12732651	Predicted analogs
155	3.1.1.47	1-alkyl-2-acetylglycerophosphocholine esterase	Q13093	PAFA_HUMAN	3D59	Predicted: Alpha/beta hydrolase	SupFam	Serine esterase	8624782, 18784071	Predicted analogs
	3.1.1.47	1-alkyl-2-acetylglycerophosphocholine esterase	P68401	PA1B2_BOVIN (beta) and PA1B3_BOVIN (gamma)	1FXW	Flavodoxin-like (beta and gamma)	SupFam	GDSL' lipolytic enzyme	8083218, 8985254	Predicted analogs (cytosolic ezyme is formed of three subunits)

156	**3.1.2.2**	Palmitoyl-CoA hydrolase	O55137	ACOT1_MOUSE	3HLK	Predicted: Alpha/beta hydrolase	SupFam	BAAT / Acyl-CoA thioester hydrolase C terminal	10567408	Predicted analogs
	**3.1.2.2**	Palmitoyl-CoA hydrolase	O00154	BACH_HUMAN	1YLI, 4HBT?	Predicted: Thioesterase/ thiol ester dehydrase-isomerase	SupFam	Thioesterase superfamily	10578051	Predicted analogs
157	**3.1.2.14**	Oleoyl-[acyl-carrier-protein] hydrolase	P08635	SAST_RAT	similar to 1C4X	Predicted: Alpha/beta hydrolase	SupFam	Thioesterase	3105579	Predicted analogs
	**3.1.2.14**	Oleoyl-[acyl-carrier-protein] hydrolase	P07149	FAS1_YEAST	1S9C	Thioesterase/thiol ester dehydrase-isomerase	SupFam	Acyl transferase	3528750, 3031066	Predicted analogs
158	**3.1.3.1**	Alkaline phosphatase	P00634	PPB_ECOLI	1ED8	Alkaline phosphatase-like	SupFam	Alkaline phosphatase	2010919	Predicted analogs
	**3.1.3.1**	Alkaline phosphatase	Q05205	PPB_LYSEN	similar to C-term of 1XZW	Predicted: Metallo-dependent phosphatases	SupFam	Calcineurin-like phosphoesterase	1856159	Predicted analogs
159	**3.1.3.3**	Phosphoserine phosphatase	Q9UBQ0	VPS29_HUMAN	1W24	Metallo-dependent phosphatases	SupFam	Calcineurin-like phosphoesterase	16737443	Predicted analogs
	**3.1.3.3**	Phosphoserine phosphatase	O07014	RSBP_BACSU	similar to 1TXO	Predicted: PP2C-like	SupFam	Protein phosphatase 2C	10632888	Predicted analogs
	3.1.3.3	Phosphoserine phosphatase	O82796	SERB_ARATH	similar to 1NNL	HAD-like	SupFam	SerB family	10196182	Hydrolyzes free Ser~P
160	**3.1.3.7**	3'(2'),5'-bisphosphate nucleotidase	Q42546	DPNP1_ARATH	similar to 1QGX	Carbohydrate phosphatase	SupFam	Inositol monophosphatase	17993620, 10205895	Predicted analogs
	**3.1.3.7**	3'(2'),5'-bisphosphate nucleotidase	O34600	NRNA_BACSU	similar to 1K20	Predicted: DHH phosphoesterases	SupFam	DHH	17586819	Predicted analogs
161	**3.1.3.27**	Phosphatidylglycerophosphatase	P18200	PGPA_ECOLI	similar to 1RFZ	Predicted: YutG-like	SupFam	Phosphatidyl-glycerophosphatase A	6296050	Predicted analogs
	**3.1.3.27**	Phosphatidylglycerophosphatase	P0A924	PGPB_ECOLI	similar to 1D2T	Predicted: Acid phosphatase/Vanadium-dependent haloperoxidase	SupFam	Phosphatidic acid phosphatase (PAP2)	6296050	Predicted analogs
162	**3.1.4.3**	Phospholipase C	P15713	PHLN_PSEAE	2D1G	Predicted: Alkaline phosphatase-like	SupFam	Phosphoesterase	2120196	Predicted analogs, by VAST
	**3.1.4.3**	Phospholipase C	Q0TV31	PHLC_CLOP1	1QM6	Phospholipase C/P1 nuclease	SupFam	Zinc dependent phospholipase C	11008117, 12009886	Predicted analogs
163	**3.1.4.4**	Phospholipase D	P20626	PLD_CORPS	-	Predicted: TIM beta/alpha-barrel (only motif)	SupFam	Pfam hit	2407718, 7934899	Predicted analogs

	**3.1.4.4**	Phospholipase D	Q53728	PLD_STRAT	1F0I	Phospholipase D/nuclease	SupFam	Phospholipase D Active site motif	7765769, 10089437, 18338352	Predicted analogs
164	**3.1.4.12**	Sphingomyelin phosphodiesterase	P17405	ASM_HUMAN	similar to 2AHD	Predicted: Metallo-dependent phosphatases	SupFam	Calcineurin-like phosphoesterase	1840600, 12631268	Predicted analogs (acid)
	**3.1.4.12**	Sphingomyelin phosphodiesterase	Q6UWV6	ENPP7_HUMAN	similar to 2GS0	PH domain-like barrel	SupFam	Type I phosphodiesterase / nucleotide pyrophosphatase	12885774	Predicted analogs (alkaline)
	**3.1.4.12**	Sphingomyelin phosphodiesterase	O60906	NSMA_HUMAN	similar to 1I9Y	Predicted: DNase I-like	SupFam	Endonuclease/ Exonuclease/phosphatase	10608884	Predicted analogs (neutral)
	**3.1.4.12**	Sphingomyelin phosphodiesterase	Q9NXE4	NSMA3_HUMAN	-	-	SupFam	Pfam hit	16517606	Predicted analogs (neutral)
165	**3.1.4.16**	2',3'-cyclic-nucleotide 2'-phosphodiesterase	Q2FZ08	CNPD_STAA8	-	Predicted: HD-domain/ PDEase-like	SupFam	2',3' cyclic nucleotide phosphodiesterase	17951247, 15853881	Predicted analogs
	**3.1.4.16**	2',3'-cyclic-nucleotide 2'-phosphodiesterase	P08331	CN16_ECOLI	similar to 2USH	5-nucleotidase and Metallo-dependent_phosphatases	SupFam	5'-nucleotidase	3005231	Predicted analogs
166	**3.1.4.17**	3',5'-cyclic-nucleotide phosphodiesterase	Q07343	PDE4B_HUMAN	1XLX	HD-domain/PDEase-like	SupFam	Cyclic nucleotide phosphodiesterase	9371714, 8384210	Predicted analogs
	**3.1.4.17**	3',5'-cyclic-nucleotide phosphodiesterase	P22434	PDE1_YEAST	similar to 1P9E	Predicted: Metallo-hydrolase/oxidoreductase	SupFam	cAMP phosphodiesterase class-II	2824992	Predicted analogs
167	**3.1.4.35**	3',5'-cyclic-GMP phosphodiesterase	B0G0Y8	PDE3_DICDI	2HD1	HD-domain/PDEase-like	SupFam	3'5'-cyclic nucleotide phosphodiesterase	11171061, 17040207	Predicted analogs
	**3.1.4.35**	3',5'-cyclic-GMP phosphodiesterase	P12019	PDE1_DICDI	similar to: 1KO2	Predicted: Metallo-hydrolase/oxidoreductase	SupFam	cAMP phosphodiesterases class-II	12429832	Predicted analogs
168	**3.1.4.53**	3',5'-cyclic-AMP phosphodiesterase	Q23917	PDE2_DICDI	2HD1	HD-domain/PDEase-like	SupFam	3'5'-cyclic nucleotide phosphodiesterase	9812977, 10488068	Predicted analogs
	**3.1.4.53**	3',5'-cyclic-AMP phosphodiesterase	P12019	PDE1_DICDI	similar to: 1KO2	Predicted: Metallo-hydrolase/oxidoreductase	SupFam	cAMP phosphodiesterases class-II	12429832	Predicted analogs
169	**3.1.13.4**	Poly(A)-specific ribonuclease	Q504Q3	PAN2_HUMAN	1WLJ (domain)	Ribonuclease H-like motif	SupFam	DnaQ-like 3'-5' exonuclease, Exonuc X-T	14583602	Predicted analogs
	**3.1.13.4**	Poly(A)-specific ribonuclease	P31384	CCR4_YEAST	similar to 1AKO	Predicted: DNase I-like	SupFam	Endonuclease/ Exonuclease/phosphatase	11889048, 11889047	Predicted analogs

170	**3.2.1.3**	Glucan 1,4-alpha-glucosidase	P69328	AMYG_ASPNG	1GAI	Alpha/alpha toroid	SupFam	Glycosyl hydrolases family 15	1527049, 8503847, 3081341	Predicted analogs
	**3.2.1.3**	Glucan 1,4-alpha-glucosidase	O43451	MGA_HUMAN	2QLY	Predicted: TIM beta/alpha-barrel	SupFam	Glycosyl hydrolases family 31	18036614, 3143729	Predicted analogs
171	**3.2.1.11**	Dextranase	P48845	DEXT_PENMI	1OGM	Dextranase N-terminal domain and Single-stranded right-handed beta-helix	SupFam	Glycosyl hydrolases family 28	8905923, 12962629	Predicted analogs
	**3.2.1.11**	Dextranase	P39653	DEXT_STRDO	similar to: 1EA9	Predicted: TIM beta/alpha-barrel	SupFam	Glycosyl hydrolase 66 family	8021165, 12030973	Predicted analogs
172	**3.2.1.45**	Glucosylceramidase	P04062	GLCM_HUMAN	2V3D	TIM beta/alpha-barrel	SupFam	O-Glycosyl hydrolase family 30	17666401	Predicted analogs
	**3.2.1.45**	Glucosylceramidase	Q9HCG7	GBA2_HUMAN	similar to 2JG0	Predicted: Alpha/alpha toroid	SupFam	DUF608, Protein of unknown function	11489889	Predicted analogs
173	**3.2.1.51**	Alpha-L-fucosidase	P04066	FUCO_HUMAN	1ODU	Glycosyl_hydrolase_domain and TIM_beta/alpha-barrel	SupFam	Glycosyl hydrolase 29 family	2894306, 2174090	Predicted analogs
	**3.2.1.51**	Alpha-L-fucosidase	Q9FXE5	FUCO3_ARATH	Predicted: 1ESC	Predicted: Flavodoxin-like	SupFam	GDSL' lipolytic enzyme	11788770	Predicted analogs
	**3.2.1.51**	Alpha-L-fucosidase	Q8L7W8	FUCO2_ARATH	2RDY	Predicted: Alpha/alpha toroid	SupFam	Glycosyl hydrolase 95 family	15262925	Predicted analogs
174	**3.2.1.58**	Glucan 1,3-ß-glucosidase	P29717	EXG_CANAL	1CZ1	TIM beta/alpha-barrel	SupFam	Glycosyl hydrolase family 5 (cellulase A)	8436950, 10610795	Predicted analogs
	**3.2.1.58**	Glucan 1,3-ß-glucosidase	P49426	EXG1_COCCA	3EQN, 1KTW	Predicted: Single-stranded right-handed beta-helix	SupFam	Glycosyl hydrolase family 55	8135518	Predicted analogs
175	**3.2.1.81**	ß-agarase	P07883	AGAR_STRCO	-	Predicted: Concanavalin A-like lectins/glucanases	SupFam	Glycosyl hydrolase family 16	3034860	Predicted analogs
	**3.2.1.81**	ß-agarase	P48839	AGAA_VIBS7	-	TIM beta/alpha-barrel	SupFam	Glycosyl hydrolase family 50	8285681	Predicted analogs
176	**3.2.1.96**	Mannosyl-glycoprotein endo-ß-N-acetylglucosaminidase	P59206	LYTB_STRR6	similar to 2ZYC	Predicted: Lysozyme-like	SupFam	Glucosaminidase	10096093	Predicted analogs
	**3.2.1.96**	Mannosyl-glycoprotein endo-ß-N-acetylglucosaminidase	P36912	EBA2_FLAME	similar to 1EOK	Predicted: TIM beta/alpha-barrel	SupFam	Glycosyl hydrolase 18 family (UniProt)	8486657, 7768917	Predicted analogs
177	**3.2.2.22**	rRNA N-glycosylase	P02879	RICI_RICCO	1APG	Ribosome inactivating proteins (RIP)	SupFam	Ribosome inactivating protein	1433290, 8780513	Predicted analogs

	**3.2.2.22**	rRNA N-glycosylase	Q1JPL7	PME18_ARATH	Predicted: 1X8Z, 1GQ8	Predicted: Bromodomain-like (N-term) and Single-stranded right-handed beta-helix (C-term)	SupFam	PMEI, Plant invertase/ pectin methylesterase inhibitor and pectinesterase	18222123	Predicted analogs
178	**3.5.1.28**	N-acetylmuramoyl-L-alanine amidase	P13016	AMPD_ECOLI	1J3G	N-acetylmuramoyl-L-alanine amidase-like	SupFam	N-acetylmuramoyl-L-alanine amidase 2	12654266	Predicted analogs
	**3.5.1.28**	N-acetylmuramoyl-L-alanine amidase	P26365	AMIB_ECOLI	C-term is similar to 1JWQ	Predicted: Phosphorylase/ hydrolase-like	SupFam	Zn-dependent exopeptidases	7511774	Predicted analogs
179	**3.6.1.6**	Nucleoside-diphosphatase	Q9WUZ9	ENTP5_MOUSE	similar to N-term of 3CJ1	Predicted: Ribonuclease H-like motif	SupFam	GDA1/CD39 NTPase	10369669	Predicted analogs
	**3.6.1.6**	Nucleoside-diphosphatase	Q8WVQ1	CANT1_HUMAN	2H2N	5-bladed beta-propeller	SupFam	Apyrase	12167635	Predicted analogs
180	**4.1.1.22**	Histidine decarboxylase	P28577	DCHS_ENTAE	similar to 1JS3	Predicted: PLP-dependent transferase-like	SupFam	Pyridoxal-dependent decarboxylase	2033044	Predicted analogs
	**4.1.1.22**	Histidine decarboxylase	P00862	DCHS_LACS3	1HQ6	Pyruvoyl-dependent histidine and arginine decarboxylases	SupFam	Histidine decarboxylase	2857718	Predicted analogs
181	**4.2.2.3**	Poly(ß-D-mannuronate) lyase	P59786	ALGL_PSEFL	similar to 1QAZ	Predicted: Alpha/alpha toroid	SupFam	Chondroitin AC/alginate lyase	12775688	Predicted analogs
	**4.2.2.3**	Poly(ß-D-mannuronate) lyase	Q59478	ALYA_KLEPN	similar to 1VAV	Predicted: Concanavalin A-like lectins/glucanases	SupFam	Concanavalin A-like lectins/glucanases	8200539	Predicted analogs
	**4.2.2.3**	Poly(ß-D-mannuronate) lyase	Q06365	ALYP_PSESO	N-term is similar to 1OFM	Predicted: Single-stranded right-handed beta-helix	SupFam	Polysaccharide lyase 6 (UniProt)	8336113, 8319887	Predicted analogs
182	**4.2.2.9**	Pectate disaccharide-lyase	Q05526	PELW_DICD3	2V8J, similar to 1FP3	Predicted: Alpha/alpha toroid	SupFam	Periplasmic pectate lyase	10383957, 1766386, 17881361	Predicted analogs
	**4.2.2.9**	Pectate disaccharide-lyase	P22751	PELX_ERWCH	C-term is similar to C-term of 1RU4	Predicted: Single-stranded right-handed beta-helix	SupFam	Polysaccharide lyase 9 (UniProt)	10383957, 2254266	Predicted analogs
183	**4.3.1.18**	D-serine ammonia-lyase	P00926	SDHD_ECOLI	similar to 1O58	Tryptophan synthase beta subunit-like PLP-dependent enzymes	SupFam	Pyridoxal-phosphate dependent enzyme	6853521, 10601247	Predicted analogs
	**4.3.1.18**	D-serine ammonia-lyase	P53095	YGU6_YEAST	Predicted: 1L6G	Predicted: TIM beta/alpha-barrel	SupFam	Alanine racemase, N-terminal domain	17937657, 18564178	Predicted analogs

184	4.99.1.4	Sirohydrochlorin ferrochelatase	P0AEA8	CYSG_ECOLI	1PJT	Rossmann-fold, Siroheme synthase middle domains-like and Tetrapyrrole methylase	SupFam	Siroheme synthase (multi doman)	8243665, 2407558, 2407234	Predicted analogs
	4.99.1.4	Sirohydrochlorin ferrochelatase	P61817	SIRB_BACME	Predicted: 1tjn	Predicted: Chelatase-like	SupFam	CbiX family, SirB subfamily	12408752	Predicted analogs
185	5.3.3.1	Steroid delta-isomerase	P00947	SDIS_COMTE	1OCV	Cystatin-like	SupFam	Nuclear transport factor 2	4753764, 10551849	Predicted analogs
	5.3.3.1	Steroid delta-isomerase	P14060	3BHS1_HUMAN	similar to 1EQ2	Predicted: Rossmann-fold	SupFam	3-beta hydroxysteroid dehydrogenase/isomerase	2139411, 2082186, 1401999	Predicted analogs
186	5.3.3.12	L-dopachrome isomerase	P40126	TYRP2_HUMAN	similar to 1WX2, 1LNL	Predicted: Di-copper centre-containing domain	SupFam	Tyrosinase	8573077	Predicted analogs
	5.3.3.12	L-dopachrome isomerase	P34884	MIF_MOUSE	2GDG	Tautomerase/MIF	SupFam	Macrophage migration inhibitory factor (MIF)	7947826, 10360941, 8900542, 9794786	Predicted analogs
187	no EC	4-Methyl-5-hydroxyethylthiazole phosphate synthase	P30139	THIG_ECOLI	1XM3	TIM beta/alpha-barrel	SupFam	Thiazole biosynthesis protein	17403671, 15012138	Predicted analogs (no EC)
	no EC	4-Methyl-5-hydroxyethylthiazole phosphate synthase	P32318	THI4_YEAST	2GJC	FAD/NAD(P)-binding domain	SupFam	Thi4 family	9367751, 12794638, 18652458	Predicted analogs (no EC)
**No.**	**EC no.**	**Enzyme name**	**NCBI**	**UniProt**	**PDB**	**SCOP fold**	**Super-family**	**Family (Pfam)**	**Ref.**	**Potential**
188	1.1.1.81	Hydroxypyruvate reductase	P70788	TTUD3_AGRVI	similar to 2B8N	GckA/TtuD-like	SupFam	MOFRL	8672817, 7592429	Potential analogs (mis-annotation)
	1.1.1.81	Hydroxypyruvate reductase	P37666	GHRB_ECOLI	similar to 2DBR, 1GDH	Predicted: Rossmann-fold and Flavodoxin-like	SupFam	D-isomer specific 2-hydroxyacid dehydrogenase	11237876	Potential analogs
189	1.1.5.2	Quinoprotein glucose dehydrogenase	P05465	DHGA_ACICA	similar to 1KV9	8-bladed beta-propeller and Cytochrome c	SupFam	Bacterial PQQ dehydrogenase	3399393, 16754970	Potential analogs (related structures)
	1.1.5.2	Quinoprotein glucose dehydrogenase	P13650	DHGB_ACICA	1CRU	6-bladed beta-propeller	SupFam	PQQ oxidoreductase gdhB	14741705	Potential analogs (related structures)

190	**1.4.1.2**	Glutamate dehydrogenase	P41755	DHE2_ACHKL	-	-	SupFam	NAD-specific glutamate dehydrogenase	8308022	Potential analogs (no papers after 1994)
	**1.4.1.2**	Glutamate dehydrogenase	P39633	DHE2_BACSU	1GTM	Rossmann-fold and Aminoacid dehydrogenase-like N-terminal domain	SupFam	Glu/Leu/Phe/Val dehydrogenases family	9829940	Potential analogs
191	**1.13.11.15**	3,4-dihydroxyphenylacetate 2,3-dioxygenase	Q45135	Q45135_9MICO	1F1X	Glyoxalase/Bleomycin resistance protein/ Dihydroxybiphenyl dioxygenase	SupFam	Glyoxalase	9179284, 15028678, 15568806, 16217642	Potential analogs
	**1.13.11.15**	3,4-dihydroxyphenylacetate 2,3-dioxygenase	Q05353	HPCB_ECOLI	-	-	SupFam	LigB	3053656, 2261999	Potential analogs (Fe dependent)
192	**1.14.11.16**	Peptide-aspartate ß-dioxygenase	Q9NWT6	HIF1N_HUMAN	1H2K	Double-stranded beta-helix and Transactivation domain	SupFam	JmjC domain	12042299, 12080085, 12432100	Potential analogs
	**1.14.11.16**	Peptide-aspartate ß-dioxygenase	Q12797	ASPH_HUMAN	-	-	SupFam	Aspartyl/asparaginyl beta-hydroxylase	7821814	Potential analogs
193	**1.14.13.7**	Phenol 2-monooxygenase	P19730, P19731	DMPL_PSEUF DMPM_PSEUF	2INP, 1HQI	Ferritin-like and beta-Grasp (ubiquitin-like) and Monooxygenase (hydroxylase) regulatory protein	SupFam	Methane/Phenol/Toluene Hydroxylase	17176061, 2254258	Potential analogs
	**1.14.13.7**	Phenol 2-monooxygenase	P15245	PH2M_TRICU	1FOH	FAD/NADP-binding domain, Thioredoxin fold and FAD-linked_reductases C-terminal domain	SupFam	Pfam hit	8145253, 9634698	Potential analogs
194	**1.14.15.3**	Alkane 1-monooxygenase	P12691	ALKB_PSEOL	-	-	SupFam	Fatty acid desaturase	2647718	Potential analogs
	**1.14.15.3**	Alkane 1-monooxygenase	Q5TCH4	CP4AM_HUMAN	similar to 1TQN	Cytochrome P450	SupFam	Cytochrome P450	14641109, 7798189, 11931657, 2361958	Potential analogs
195	**2.3.1.23**	1-acylglycerophosphocholine O-acyltransferase	O42916	ALE1_SCHPO	-	-	SupFam	MBOAT family, integral membrane protein	17890783	Potential analogs
	**2.3.1.23**	1-acylglycerophosphocholine O-acyltransferase	Q06510	TAZ1_YEAST	similar to 1IUQ	Predicted: Glycerol-3-phosphate (1)-acyltransferase	SupFam	Acyltransferase	15588229, 16135531	Potential analogs

196	**2.3.1.75**	Long-chain-alcohol O-fatty-acyltransferase	Q93ZR6	WSD1_ARATH	-	-	SupFam	N-term UPF0089 family	18621978	Potential analogs (activity?)
	**2.3.1.75**	Long-chain-alcohol O-fatty-acyltransferase	Q9XGY6	WAXS1_SIMCH	-	-	SupFam	Wax synthase	10712527	Potential analogs
	**2.3.1.75**	Long-chain-alcohol O-fatty-acyltransferase	Q58HT5	AWAT1_HUMAN	-	-	SupFam	Diacylglycerol acyltransferase	15671038	Potential analogs
197	**2.3.1.88**	Peptide alpha-N-acetyltransferase	P41227	ARD1A_HUMAN	similar to 1MK4	Acyl-CoA N-acyltransferases (Nat)	SupFam	GNAT superfamily	7981673, 15496142	Potential analogs
	**2.3.1.88**	Peptide alpha-N-acetyltransferase	P37293	NAT2_YEAST	-	-	SupFam	No Pfam hits	8175741, 9128142	Potential analogs
198	**2.3.2.13**	Protein-glutamine gamma-glutamyltransferase	P00488	F13A_HUMAN, TGAS_STRMB	1FIE, 1IU4	Cysteine proteinases	SupFam	Transglutaminase, microbial transglutaminase	9839945, 2901091, 2877456	Potential analogs
	**2.3.2.13**	Protein-glutamine gamma-glutamyltransferase	P40746	TGL_BACSU	-	-	SupFam	Bacillus TGase	9692191	Potential analogs
199	**2.4.1.69**	Galactoside 2-alpha-L-fucosyltransferase	Q9TUD4	FUT1_GORGO	-	-	SupFam	Glycosyltransferase family 11	10723735, 7372640, 8613146	Potential analogs
	**2.4.1.69**	Galactoside 2-alpha-L-fucosyltransferase	Q9M5Q1	FUT1_PEA	-	-	SupFam	Xyloglucan fucosyltransferase (Glycosyltransferase family 37)	10747946	Potential analogs
	**2.4.1.69**	Galactoside 2-alpha-L-fucosyltransferase	Q54QG0	PGFB_DICDI	N-term is similar to 1H7Q	Predicted: Nucleotide-diphospho-sugar transferases (N-term)	SupFam	Glycosyltransferase family 2 (UniProt)	11423539, 12244067	Potential analogs
200	**2.4.1.226**	N-acetylgalactosaminyl-proteoglycan 3-ß-glucuronosyltransferase	Q8IZ52	CHSS2_HUMAN	-	-	SupFam	Chondroitin N-acetylgalactosaminyl-transferase	12761225	Potential analogs
	**2.4.1.226**	N-acetylgalactosaminyl-proteoglycan 3-ß-glucuronosyltransferase	Q8L0V4	CHS_ECOLX	2Z86	Predicted: Nucleotide-diphospho-sugar transferases (N-term and C-term)	SupFam	Glycosyltransferase 2	11943778	Potential analogs
201	**2.7.7.24**	Glucose-1-phosphate thymidylyltransferase	P61887	RMLA2_ECOLI	1MC3	Nucleotide-diphospho-sugar transferases	SupFam	Nucleotidyl transferase	7559340, 12171937	Potential analogs
	**2.7.7.24**	Glucose-1-phosphate thymidylyltransferase	P29785	STRO_STRGR	Predicted: 1LBV	Predicted: Carbohydrate phosphatase	SupFam	Inositol monophosphatase family	1661369	Potential analogs (activity?)

202	**2.7.7.50**	mRNA guanylyltransferase	Q01159	MCE1_YEAST	1P16	ATP-grasp (catalytic domain)	SupFam	Eukaryotic GTase	6389537, 3029058, 6094533	Potential analogs
	**2.7.7.50**	mRNA guanylyltransferase	P11079	LMBD2_REOVD	1EJ6	Low-resolution structure 1EJ6 does not allow comparison	SupFam	Orthoreovirus lambda-2	10801118	Potential analogs
	**2.7.7.50**	mRNA guanylyltransferase	A2T3S5	VP3_ROTSH	-	-	SupFam	Rotavirus VP3	10603323	Potential analogs
203	**2.7.8.5**	CDP-diacylglycerol-glycerol-3-phosphate 3-phosphatidyltransferase	P25578	PGPS1_YEAST	Predicted: 1BYS	Predicted: Phospholipase D/ nuclease	SupFam	Phospholipase D (CDP-alcohol phosphatidyltransferase class-I, UniProt)	9545322	Potential analogs
	**2.7.8.5**	CDP-diacylglycerol-glycerol-3-phosphate 3-phosphatidyltransferase	P0ABF8	PGSA_ECOLI	-	-	SupFam	CDP-alcohol phosphatidyltransferase	1323047, 8195097	Potential analogs
204	**2.7.8.8**	CDP-diacylglycerol-serine O-phosphatidyltransferase	P23830	PSS_ECOLI	similar to 1BYR	Predicted: Phospholipase D/ nuclease	SupFam	Phospholipase D Active site motif	1323044, 10074947	Potential analogs
	**2.7.8.8**	CDP-diacylglycerol-serine O-phosphatidyltransferase	P08456	PSS_YEAST	-	Integral membrane protein	SupFam	CDP-alcohol phosphatidyltransferase	3040403	Potential analogs
205	**2.7.13.3**	Histidine kinase	P0AEJ4	ENVZ_ECOLI	1BXD	ATPase domain of HSP90 chaperone/DNA topoisomerase II/histidine kinase	SupFam	Histidine kinase domain	10426948, 1323560	Potential analogs
	**2.7.13.3**	Histidine kinase	Q9AWA5	GWD1_SOLTU	-	-	SupFam	Pyruvate phosphate dikinase, PEP/pyruvate binding domain	12011472	Potential analogs (chloroplast precursor)
206	**3.1.1.32**	Phospholipase A1	P0A921	PA1_ECOLI	1QD5	Transmembrane beta-barrels	SupFam	Phospholipase A1	8300539	Potential analogs
	**3.1.1.32**	Phospholipase A1	P18952	PA1_SERLI	-	Predicted: Alpha/beta hydrolase	SupFam	Pfam hit	3056919	Potential analogs
207	**3.1.3.6**	3'-nucleotidase	P0A840	SURE_ECOLI	1ILV	SurE-like	SupFam	SurE nucleotidase	15489502	Potential analogs
	**3.1.3.6**	3'-nucleotidase	P24504	NUP3_PENSQ	1AK0	Phospholipase C/P1 nuclease	SupFam	S1/P1 Nuclease	1964878, 1369324	Potential analogs
20	**3.1.3.78**	Phosphatidylinositol-4,5-bisphosphate 4-phosphatase	Q86T03	TM55B_HUMAN)	-	-	SupFam	Transmembrane protein 55A	16365287	Potential analogs
	**3.1.3.78**	Phosphatidylinositol-4,5-bisphosphate 4-phosphatase	Q07566	IPGD_SHIFL	-	-	SupFam	Enterobacterial virulence protein IpgD	12356723, 11029686	Potential analogs

209	**3.2.1.132**	Chitosanase	Q9P961	Q9P961_ASPOR	-	-	SupFam	Fungal chitosanase	11388486	Potential analogs
	**3.2.1.132**	Chitosanase	O07921	CHIS_BACSU	1CHK	Lysozyme-like	SupFam	Glycosyl hydrolase family 46	10658653, 10521473	Potential analogs
210	**3.2.1.143**	Poly(ADP-ribose) glycohydrolase	Q9NX46	ARHL2_HUMAN	2FOZ, similar to 1T5J	ADP-ribosylglycohydrolase	SupFam	ADP-ribosylglycohydrolase	16278211	Potential analogs
	**3.2.1.143**	Poly(ADP-ribose) glycohydrolase	Q86W56	PARG_HUMAN	-	-	SupFam	Poly (ADP-ribose) glycohydrolase (PARG)	10449915, 9115250	Potential analogs
211	**3.2.2.5**	NAD^+ nucleosidase (ADP ribosyl cyclase)	P28907	CD38_HUMAN	2EF1	Flavodoxin-like	SupFam	Ribosyl hydrolyase	8253715, 16154090	Potential analogs
	**3.2.2.5**	NAD^+ nucleosidase (ADP ribosyl cyclase)	A2RCA5	A2RCA5_STRPG	-	-	SupFam	NAD glycohydrolase	15627505, 16177331, 18048938	Potential analogs
212	**3.5.1.23**	Ceramidase	Q13510	ASAH1_HUMAN	Predicted: 3PVA	Predicted: Ntn hydrolase-like	SupFam	Choloylglycine hydrolase	7744740, 8955159, 10993717	Potential analogs (acid)
	**3.5.1.23**	Ceramidase	Q5QJU3	ASA3L_HUMAN	-	-	SupFam	Alkaline phytoceramidase	11356846	Potential analogs (alkaline)
	**3.5.1.23**	Ceramidase	Q9NR71	ASAH2_HUMAN	similar to 2ZXC	-	SupFam	Neutral/alkaline non-lysosomal ceramidase	10781606, 16229686	Potential analogs (neutral)
213	**3.6.1.27**	Undecaprenyl-diphosphatase	P80143	UDDP_SULAC	C-term is similar to C-term of 1QI9	Predicted: Acid phosphatase/ Vanadium-dependent haloperoxidase	SupFam	PAP2 superfamily	1321720	Potential analogs
	**3.6.1.27**	Undecaprenyl-diphosphatase	P60932	UPPP_ECOLI	-	-	SupFam	Bacitracin resistance protein BacA	15778224	Potential analogs
**No.**	**EC no.**	**Enzyme name and REACTION**	**NCBI**	**UniProt**	**PDB**	**SCOP fold**	**Super-family**	**Family (Pfam)**	**Ref.**	**Comments**
214	**4.1.1.11**	Aspartate 1-decarboxylase (L-Aspartate <=> ß-Alanine + CO_2)	P0A790	PAND_ECOLI	1AW8	Double psi beta-barrel	SupFam	Aspartate decarboxylase	6767707	Two distinct structural folds
	**4.1.1.15**	Glutamate decarboxylase (L-Aspartate <=> ß-Alanine + CO_2)	Q05329	DCE2_HUMAN	1JS3	PLP-dependent transferase-like	SupFam	Pyridoxal-dependent decarboxylase	6956856, 658037	

215	**1.4.3.21**	Primary-amine oxidase (Tyramine + H_2O + Oxygen <=> 4-Hydroxyphenylacetaldehyde + NH_3 + H_2O_2)	P49250	AMO_KLEAE	1OAC	Supersandwich and Cystatin-like and N domain of copper amine oxidase-like	SupFam	Copper amine oxidase	1556068	Two distinct structural folds
	**1.4.3.4**	Amine oxidase (flavin-containing) (Tyramine + H_2O + Oxygen <=> 4-Hydroxyphenylacetaldehyde + NH_3 + H_2O_2)	Q6NSN2	AOF_DANRE	1O5W	FAD/NADP-binding domain and FAD-linked reductases C-terminal domain	SupFam	Flavin monoamine oxidase	16917825	
216	**2.7.4.14**	Cytidylate kinase (ATP + UMP <=> ADP + UDP)	Q5EBM0	CMPK2_HUMAN	similar to 2TMK	P-loop containing NTPases	SupFam	Thymidylate kinase	17999954	Two distinct structural folds
	**2.7.4.22**	UMP kinase (ATP + UMP <=> ADP + UDP)	P0A7E9	PYRH_ECOLI	2BNE	Carbamate kinase-like	SupFam	Amino acid kinase	7711027	
217	**3.1.3.62**	multiple inositol-polyphosphate phosphatase (myo-Inositol hexakisphosphate + H2O <=> 1L-myo-Inositol 1,2,3,4,6-pentakisphosphate + Orthophosphate)	O35217	MINP1_RAT	similar to 1QWO	Phosphoglycerate mutase-like	SupFam	Multiple inositol-polyphosphate phosphatase	9359836	Two distinct structural folds
	**3.1.3.72**	5-phytase; Systematic name: myo-inositol-hexakisphosphate 5-phosphohydrolase (myo-Inositol hexakisphosphate + H_2O <=> 1L-myo-Inositol 1,2,3,4,6-pentakisphosphate + Orthophosphate)	Q7WUJ1	Q7WUJ1_SELRU	1U24	Phosphotyrosine protein phosphatases II	SupFam	Pfam hit	17567745	
218	**3.2.1.93**	Alpha,-alpha-phosphotrehalase (H2O + alpha,alpha'-Trehalose 6-phosphate <=> D-Glucose + D-Glucose 6-phosphate)	P39795	TREC_BACSU	1UOK	TIM beta/alpha-barrel and Glycosyl hydrolase domain	SupFam	Alpha amylase	7751281	Two distinct structural folds
	**3.2.1.122**	Maltose-6'-phosphate glucosidase (H2O + alpha, alpha'-Trehalose 6-phosphate <=> D-Glucose + D-Glucose 6-phosphate)	O06901	MALH_FUSMR	1U8X	LDH C-terminal domain-like and Rossmann-fold	SupFam	Family 4 glycosyl hydrolase	11882720, 7730284	
219	**3.5.1.2**	Glutaminase (L-Glutamine + H_2O <=> L-Glutamate + NH_3)	Q9UI32	GLSL_HUMAN	3CZD	Beta-lactamase/ transpeptidase-like	SupFam	Glutaminase	18459799	Two distinct structural folds
	**3.5.1.38**	Glutamin-(asparagin-)ase (L-Glutamine + H_2O <=> L-Glutamate + NH_3)	Q88K39	ASPQ_PSEPK, ASPG2_ECOLI	1DJO	Glutaminase/ Asparaginase	SupFam	Asparaginase	10684596, 8434007, 1521538	

220	**3.8.1.2**	(S)-2-haloacid dehalogenase ((S)-2-Haloacid + H_2O <=> (R)-2-Hydroxyacid + Halide)	Q52087	HADL_PSEPU	1QH9	HAD-like	SupFam	Haloacid dehalogenase-like hydrolase	1588303	Two distinct structural folds
	**3.8.1.10**	2-haloacid dehalogenase (configuration-inverting) ((S)-2-Haloacid + H_2O <=> (R)-2-Hydroxyacid + Halide)	O06652	HADDL_PSES4	3BJX	-	SupFam	Halocarboxylic acid dehydrogenase DehI	9209038	
221	**5.4.2.2**	Phosphoglucomutase (Alpha-D-Ribose 1-phosphate <=> D-Ribose 5-phosphate)	P00949	PGM1_RABIT	1JDY	Phosphoglucomutase first 3 domains and TBP-like	SupFam	Phosphoglucomutase/ phosphomannomutase	15299905, 1328221	Two distinct structural folds
	**5.4.2.7**	Phosphopentomutase (Alpha-D-Ribose 1-phosphate <=> D-Ribose 5-phosphate)	P0A6K6	DEOB_ECOLI	2I09	Alkaline phosphatase-like and DeoB insert domain-like	SupFam	Phosphopentomutase	4904508	
222	**1.3.1.44**	Trans-2-enoyl-CoA reductase (NAD^+) (Butanoyl-CoA + NAD^+ <=> Crotonoyl-CoA + NADH + H^+)	Q5EU90	TER_EUGGR	-	Predicted: Rossmann-fold	SupFam	Short-chain alcohol dehydrogenase	15569691	Predicted analogs
	**1.3.99.2**	Butyryl-CoA dehydrogenase (Butanoyl-CoA + NAD^+ <=> Crotonoyl-CoA + NADH + H^+)	Q06319	ACDS_MEGEL, ACADS_HUMAN	1BUC	Bromodomain-like and Acyl-CoA dehydrogenase NM domain-like	SupFam	Acyl-CoA dehydrogenase	8399220, 7857927	Predicted analogs
223	**2.5.1.44**	Homospermidine synthase (Putrescine + Spermidine <=> Homospermidine + 1,3-Diaminopropane)	O32323	HSS_RHOVI	2PH5	Predicted: Rossman-fold and FwdE/GAPDH domain-like	SupFam	Homospermidine synthase	8841401	Predicted analogs
	**2.5.1.45**	Homospermidine synthase (spermidine-specific) (Putrescine + Spermidine <=> Homospermidine + 1,3-Diaminopropane)	Q9SC13	HSS1_SENVE	1RLZ	DHS-like NAD/FAD-binding domain	SupFam	Deoxyhypusine synthase	10611289	Predicted analogs
224	*2.7.1.31*	Glycerate 2-kinase	P77364	GLXK1_ECOLI	1TO6	Glycerate kinase I	SupFam	Glycerate kinase type-1	9772162	Two distinct structural folds
	2.7.1.31	Glycerate 2-kinase	Q8IVS8	GLCTK_HUMAN	smilar to 2B8N	GckA/TtuD-like	SupFam	Glycerate kinase type-2 (contains MOFRL domain)	16753811, 16865707	
