# Supplementary material for: Non-homologous isofunctional enzymes: A systematic analysis of alternative solutions in enzyme evolution
Source: Biol Direct. 2010 Apr 30;5:31. doi: 10.1186/1745-6150-5-31 (PMC2876114; doi:10.1186/1745-6150-5-31)
Supplement: Additional file 3 — Supplementary Tables S3-S9 [file 1745-6150-5-31-S3.PDF]

Supplementary Table S1. An update of the original 1998 listing of 105 EC nodes with predicted analogous enzymes (online)

Supplementary Table S2. New listing of confirmed and predicted analogous enzymes (online)

Supplementary Table S3. Statistics of analogous enzyme searches in the ENZYME and KEGG databases

	ENZYME database	KEGG database	ENZYME & KEGG combined set
Four-digit EC nodes	4890	–	–
EC nodes with at least one assigned sequence	2487	2256	–
Without proteases sequences	2187	2041	2637
Without remaining single sequences	1893	1934	2181
Multisubunit enzyme EC nodes to be excluded	4	4	4
EC nodes selected for clustering	1889	1930	2177

Supplementary Table S4. Combined results of clustering sequences from the ENZYME and KEGG databases

		**ENZYME**	**ENZYME**	**ENZYME**	**ENZYME**	**ENZYME**	**ENZYME**	**ENZYME**	
	Number of clusters per EC node	**0**	**1**	**2**	**3**	**4**	**5**	**>5**	**Total**
**KEGG**	**0**	-	223	20	3	-	1	-	247
**KEGG**	**1**	230	944	59	10	1	-	1	1245
**KEGG**	**2**	31	246	103	20	5	1	1	407
**KEGG**	**3**	16	73	37	20	8	2	-	156
**KEGG**	**4**	9	19	15	12	4	2	-	61
**KEGG**	**5**	1	9	3	6	2	5	2	28
**KEGG**	**>5**	1	5	7	1	5	1	13	33
**Total**		288	1519	244	72	25	12	17	2177

Each cell shows the number of EC nodes whose sequences clustered into the specified number of clusters (1 – 5 or more than 5) in KEGG and ENZYME databases. Only about half of EC nodes (yellow highlighting) had the same numbers of clusters in the two databases.

Supplementary Table S5. Distribution of analogous enzymes among enzyme classes

	Enzyme class	Analogs (EC)	Analyzed set (EC)	Expect	Chi2	P(Chi2)
1	Oxidoreductase	36	616	52.5	4.9	0.16
2	Transferase	27	676	57.7	15.8	4.30×10^{-4}
3	Hydrolase	87	450	38.4	60.3	4.78×10^{-14}
4	Lyase	18	224	19.1	0.0	5.34
5	Isomerase	17	106	9.0	6.2	0.08
6	Ligase	1	109	9.3	6.5	0.06
	Total	186	2181			

Supplementary Table S6. Distribution of analogous enzymes by COG category

COG category	Analogs	All EC analyzed	Expect	Chi2	P(Chi2)
B	1	6	0.51	0.00	21.00
N	1	3	0.26	0.23	13.22
V	2	14	1.19	0.08	16.37
K	3	23	1.96	0.15	14.71
J	5	71	6.06	0.05	17.25
O	5	50	4.26	0.01	19.09
ox stress	6	18	1.54	10.24	0.03
H	7	159	13.56	2.71	2.10
T	7	59	5.03	0.43	10.77
L	8	29	2.47	10.22	0.03
M	8	173	14.75	2.65	2.17
P	9	67	5.71	1.36	5.12
Q	9	207	17.65	3.77	1.10
F	13	110	9.38	1.04	6.48
R	13	192	16.37	0.50	10.03
C	16	215	18.34	0.18	14.03
E	17	312	26.61	3.12	1.63
I	21	193	16.46	0.99	6.70
G	35	270	23.03	5.72	0.35

Supplementary Table S7. Distribution of analogous enzymes among most common folds

	Fold	Analogs	All	Expect	Chi2	P(Chi2)
1	TIM beta/alpha-barrel	51	250	21.32	39.94	2.62×10^{-9}
2	Rossmann-fold	28	212	18.08	4.91	0.27
3	Alpha/beta hydrolase	12	40	3.41	19.18	1.19×10^{-4}
4	Metallo-dependent phosphatases	11	16	1.36	61.16	5.25×10^{-14}
5	Ribonuclease H-like motif	12	32	2.73	28.19	1.10×10^{-6}
6	Alpha/alpha toroid	10	33	2.81	15.88	6.74×10^{-4}
7	Flavodoxin-like	10	63	5.37	3.17	0.75
8	P-loop containing NTPase	9	94	8.02	0.03	8.64
9	Ferredoxin-like	8	77	6.57	0.13	7.16
10	HAD-like	8	37	3.16	5.98	0.14
	Total EC nodes	186	2181			

Supplementary Table S8. Phylogenetic patterns of analogous enzymes involved in defense against reactive oxygen species

Enzyme (EC)	Examples	Instances in bacteria (out of 607)	Instances in archaea (out of 48)	Instances in eukaryotes (out of 63)
Superoxide dismutase (EC 1.15.1.1)	SODF_ECOLI<br>SODC_ECOLI<br>SODN_STRSO<br>NEC1_NICLS	526<br>249<br>32<br>6	23<br>3<br>0<br>0	62<br>47<br>1<br>3
Catalase (EC 1.11.1.6)	CATA_HUMAN<br>CATG_ECOLI<br>MCAT_LACPL	323<br>217<br>122	6<br>6<br>6	49<br>21<br>0
Peroxidase (EC 1.11.1.7)	PRDX6_MOUSE<br>PERM_HUMAN<br>YCDB_ECOLI	551<br>31<br>247	45<br>**0**<br>0	55<br>25<br>7
Chloride peroxidase (EC 1.11.1.10)	PRXC_PSEPY<br>PRXC_CURIN<br>PRXC_CALFU	31<br>221<br>0	0<br>9<br>0	4<br>9<br>9
Peroxiredoxin (EC 1.11.1.15)	TDXH_AERPE<br>AHPD_MYCTU<br>OSMC_ECOLI	564<br>426<br>437	45<br>30<br>21	61<br>24<br>7

Supplementary Table S9. Complete genomes used in the analysis of phylogenetic distribution of analogous enzymes

No.	KEGG organism name	KEGG symbol	NCBI Taxonomy name	TaxID	Proteins	Domain
1	Homo sapiens (human)	hsa	Homo sapiens	9606	24329	Eukaryotes
2	Pan troglodytes (chimpanzee)	ptr	Pan troglodytes	9598	25189	Eukaryotes
3	Macaca mulatta (rhesus monkey)	mcc	Macaca mulatta	9544	23980	Eukaryotes
4	Mus musculus (mouse)	mmu	Mus musculus	10090	29561	Eukaryotes
5	Rattus norvegicus (rat)	rno	Rattus norvegicus	10116	26231	Eukaryotes
6	Canis familiaris (dog)	cfa	Canis lupus familiaris	9615	19823	Eukaryotes
7	Bos taurus (cow)	bta	Bos taurus	9913	24151	Eukaryotes
8	Sus scrofa (pig)	ssc	Sus scrofa	9823	1752	Eukaryotes
9	Monodelphis domestica (opossum)	mdo	Monodelphis domestica	13616	19136	Eukaryotes
10	Ornithorhynchus anatinus (platypus)	oaa	Ornithorhynchus anatinus	9258	16405	Eukaryotes
11	Gallus gallus (chicken)	gga	Gallus gallus	9031	18131	Eukaryotes
12	Xenopus laevis (African clawed frog)	xla	Xenopus laevis	8355	10379	Eukaryotes
13	Xenopus tropicalis (western clawed frog)	xtr	Xenopus (Silurana) tropicalis	8364	7115	Eukaryotes
14	Danio rerio (zebrafish)	dre	Danio rerio	7955	35046	Eukaryotes
15	Strongylocentrotus purpuratus (purple sea urchin)	spu	Strongylocentrotus purpuratus	7668	28905	Eukaryotes
16	Nematostella vectensis (sea anemone)	nve	Nematostella vectensis	45351	24773	Eukaryotes
17	Drosophila melanogaster (fruit fly)	dme	Drosophila melanogaster	7227	14168	Eukaryotes
18	Drosophila pseudoobscura	dpo	Drosophila pseudoobscura pseudoobscura	46245	9869	Eukaryotes
19	Anopheles gambiae (mosquito)	aga	Anopheles gambiae str. PEST	180454	13011	Eukaryotes
20	Tribolium castaneum (red flour beetle)	tca	Tribolium castaneum	7070	9476	Eukaryotes
21	Caenorhabditis elegans (nematode)	cel	Caenorhabditis elegans	6239	20101	Eukaryotes
22	Ostreococcus lucimarinus	olu	Ostreococcus lucimarinus CCE9901	436017	7603	Eukaryotes
23	Chlamydomonas reinhardtii	cre	Chlamydomonas reinhardtii	3055	14473	Eukaryotes
24	Oryza sativa japonica (Japanese rice)	osa	Oryza sativa Japonica Group	39947	28662	Eukaryotes
25	Physcomitrella patens subsp. patens	ppp	Physcomitrella patens subsp. patens	145481	36127	Eukaryotes
26	Arabidopsis thaliana (thale cress)	ath	Arabidopsis thaliana	3702	27079	Eukaryotes
27	Saccharomyces cerevisiae	sce	Saccharomyces cerevisiae	4932	6298	Eukaryotes
28	Ashbya gossypii (Eremothecium gossypii)	ago	Ashbya gossypii ATCC 10895	284811	4750	Eukaryotes
29	Kluyveromyces lactis	kla	Kluyveromyces lactis NRRL Y-1140	284590	5360	Eukaryotes
30	Debaryomyces hansenii	dha	Debaryomyces hansenii CBS767	284592	6351	Eukaryotes
31	Pichia stipitis	pic	Pichia stipitis CBS 6054	322104	5816	Eukaryotes
32	Vanderwaltozyma polyspora	vpo	Vanderwaltozyma polyspora DSM 70294	436907	5359	Eukaryotes
33	Candida glabrata	cgr	Candida glabrata CBS 138	284593	5217	Eukaryotes
34	Yarrowia lipolytica	yli	Yarrowia lipolytica CLIB122	284591	6572	Eukaryotes
35	Schizosaccharomyces pombe	spo	Schizosaccharomyces pombe 972h-	284812	5031	Eukaryotes
36	Neurospora crassa	ncr	Neurospora crassa OR74A	367110	9824	Eukaryotes
37	Podospora anserina	pan	Podospora anserina DSM 980	515849	10200	Eukaryotes
38	Magnaporthe grisea	mgr	Magnaporthe grisea 70-15	242507	14010	Eukaryotes
39	Fusarium graminearum	fgr	Gibberella zeae PH-1	229533	11686	Eukaryotes
40	Aspergillus nidulans	ani	Aspergillus nidulans FGSC A4	227321	9541	Eukaryotes
41	Aspergillus fumigatus	afm	Aspergillus fumigatus Af293	330879	9630	Eukaryotes
42	Aspergillus oryzae	aor	Aspergillus oryzae RIB40	510516	12099	Eukaryotes
43	Aspergillus niger	ang	Aspergillus niger CBS 513.88	425011	14129	Eukaryotes
44	Cryptococcus neoformans JEC21	cne	Cryptococcus neoformans var. neoformans JEC21	214684	6273	Eukaryotes
45	Cryptococcus neoformans B-3501A	cnb	Cryptococcus neoformans var. neoformans B-3501A	283643	6500	Eukaryotes
46	Laccaria bicolor	lbc	Laccaria bicolor S238N-H82	486041	18215	Eukaryotes
47	Ustilago maydis	uma	Ustilago maydis 521	237631	6562	Eukaryotes
48	Malassezia globosa	mgl	Malassezia globosa CBS 7966	425265	4286	Eukaryotes
49	Encephalitozoon cuniculi	ecu	Encephalitozoon cuniculi GB-M1	284813	1996	Eukaryotes
50	Monosiga brevicollis	mbr	Monosiga brevicollis MX1	431895	9230	Eukaryotes
51	Giardia lamblia	gla	Giardia lamblia ATCC 50803	184922	6500	Eukaryotes
52	Dictyostelium discoideum	ddi	Dictyostelium discoideum AX4	352472	13458	Eukaryotes
53	Plasmodium falciparum	pfa	Plasmodium falciparum 3D7	36329	5261	Eukaryotes
54	Plasmodium yoelii	pyo	Plasmodium yoelii yoelii str. 17XNL	352914	7353	Eukaryotes
55	Cryptosporidium parvum	cpv	Cryptosporidium parvum Iowa II	353152	3805	Eukaryotes
56	Cryptosporidium hominis	cho	Cryptosporidium hominis TU502	353151	3885	Eukaryotes
57	Theileria annulata	tan	Theileria annulata strain Ankara	353154	3795	Eukaryotes
58	Theileria parva	tpv	Theileria parva strain Muguga	333668	4087	Eukaryotes

59	Tetrahymena thermophila	tet	Tetrahymena thermophila	5911	26066	Eukaryotes
60	Trypanosoma brucei	tbr	Trypanosoma brucei TREU927	185431	8712	Eukaryotes
61	Trypanosoma cruzi	tcr	Trypanosoma cruzi strain CL Brener	353153	19607	Eukaryotes
62	Leishmania major	lma	Leishmania major strain Friedlin	347515	8264	Eukaryotes
63	Entamoeba histolytica	ehi	Entamoeba histolytica HM-1:IMSS	294381	11065	Eukaryotes
64	Escherichia coli K-12 MG1655	eco	Escherichia coli str. K-12 substr. MG1655	511145	4467	Bacteria
65	Escherichia coli K-12 W3110	ecj	Escherichia coli str. K-12 substr. W3110	316407	4494	Bacteria
66	Escherichia coli K-12 DH10B	ecd	Escherichia coli str. K-12 substr. DH10B	316385	4357	Bacteria
67	Escherichia coli O157 EDL933 (EHEC)	ece	Escherichia coli O157:H7 EDL933	155864	5554	Bacteria
68	Escherichia coli O157 Sakai (EHEC)	ecs	Escherichia coli O157:H7 str. Sakai	386585	5483	Bacteria
69	Escherichia coli CFT073 (UPEC)	ecc	Escherichia coli CFT073	199310	5589	Bacteria
70	Escherichia coli UTI89 (UPEC)	eci	Escherichia coli UTI89	364106	5299	Bacteria
71	Escherichia coli 536 (UPEC)	ecp	Escherichia coli 536	362663	4789	Bacteria
72	Escherichia coli APEC O1	ecv	Escherichia coli APEC O1	405955	4996	Bacteria
73	Escherichia coli E24377A	ecw	Escherichia coli E24377A	331111	5264	Bacteria
74	Escherichia coli HS	ecx	Escherichia coli HS	331112	4636	Bacteria
75	Escherichia coli SECEC	ecm	Escherichia coli SMS-3-5	439855	5128	Bacteria
76	Escherichia coli ATCC 8739	ecl	Escherichia coli ATCC 8739	481805	4409	Bacteria
77	Salmonella enterica serovar Typhi CT18	sty	Salmonella enterica subsp. enterica serovar Typhi str. CT18	220341	5086	Bacteria
78	Salmonella enterica serovar Typhi Ty2	stt	Salmonella enterica subsp. enterica serovar Typhi str. Ty2	209261	4645	Bacteria
79	Salmonella enterica serovar Paratyphi A	spt	Salmonella enterica subsp. enterica serovar Paratyphi A str. ATCC 9150	295319	4403	Bacteria
80	Salmonella enterica serovar Paratyphi B	spq	Salmonella enterica subsp. enterica serovar Paratyphi B str. SPB7	272994	5779	Bacteria
81	Salmonella typhimurium LT2	stm	Salmonella enterica subsp. enterica serovar Typhimurium str. LT2	99287	4734	Bacteria
82	Salmonella enterica subsp. arizonae	ses	Salmonella enterica subsp. arizonae serovar 62:z4,z23:--	41514	4699	Bacteria
83	Yersinia pestis CO92	ype	Yersinia pestis CO92	214092	4331	Bacteria
84	Yersinia pestis KIM	ypk	Yersinia pestis KIM 10	187410	4359	Bacteria
85	Yersinia pestis Mediaevails	ypm	Yersinia pestis biovar Microtus str. 91001	229193	4405	Bacteria
86	Yersinia pestis Antiqua	ypa	Yersinia pestis Antiqua	360102	4471	Bacteria
87	Yersinia pestis Nepal516	ypn	Yersinia pestis Nepal516	377628	4204	Bacteria
88	Yersinia pestis Pestoides	ypp	Yersinia pestis Pestoides F	386656	4263	Bacteria
89	Yersinia pestis Angola	ypg	Yersinia pestis Angola	349746	4469	Bacteria
90	Yersinia pseudotuberculosis IP32953	yps	Yersinia pseudotuberculosis IP 32953	273123	4238	Bacteria
91	Yersinia pseudotuberculosis IP31758	ypi	Yersinia pseudotuberculosis IP 31758	349747	4494	Bacteria
92	Yersinia pseudotuberculosis YPIII	ypy	Yersinia pseudotuberculosis YPIII	502800	4305	Bacteria
93	Yersinia enterocolitica	yen	Yersinia enterocolitica subsp. enterocolitica 8081	393305	4281	Bacteria
94	Shigella flexneri 301 (serotype 2a)	sfl	Shigella flexneri 2a str. 301	198214	4835	Bacteria
95	Shigella flexneri 2457T (serotype 2a)	sfx	Shigella flexneri 2a str. 2457T	198215	4577	Bacteria
96	Shigella flexneri 8401 (serotype 5b)	sfv	Shigella flexneri 5 str. 8401	373384	4434	Bacteria
97	Shigella sonnei	ssn	Shigella sonnei Ss046	300269	4809	Bacteria
98	Shigella boydii	sbo	Shigella boydii Sb227	300268	4617	Bacteria
99	Shigella boydii CDC 3083-94	sbc	Shigella boydii CDC 3083-94	344609	5336	Bacteria
100	Shigella dysenteriae	sdy	Shigella dysenteriae Sd197	300267	4897	Bacteria
101	Erwinia carotovora	eca	Pectobacterium atrosepticum SCRI1043	218491	4614	Bacteria
102	Photorhabdus luminescens	plu	Photorhabdus luminescens subsp. laumondii TTO1	243265	5012	Bacteria
103	Buchnera aphidicola APS	buc	Buchnera aphidicola str. APS (Acyrthosiphon pisum)	107806	618	Bacteria
104	Buchnera aphidicola Sg	bas	Buchnera aphidicola str. Sg (Schizaphis graminum)	198804	619	Bacteria
105	Buchnera aphidicola Bp	bab	Buchnera aphidicola str. Bp (Baizongia pistaciae)	224915	553	Bacteria
106	Buchnera aphidicola str. Cc	bcc	Buchnera aphidicola str. Cc (Cinara cedri)	372461	397	Bacteria
107	Wigglesworthia glossinidia	wbr	Wigglesworthia glossinidia endosymbiont of Glossina brevipalpis	36870	657	Bacteria
108	Sodalis glossinidius	sgl	Sodalis glossinidius str. 'morsitans'	343509	2607	Bacteria
109	Enterobacter sp. 638	ent	Enterobacter sp. 638	399742	4385	Bacteria
110	Enterobacter sakazakii	esa	Cronobacter sakazakii ATCC BAA-894	290339	4571	Bacteria
111	Klebsiella pneumoniae	kpn	Klebsiella pneumoniae subsp. pneumoniae MGH 78578	272620	5306	Bacteria
112	Citrobacter koseri ATCC BAA-895	cko	Citrobacter koseri ATCC BAA-895	290338	5171	Bacteria
113	Serratia proteamaculans	spe	Serratia proteamaculans 568	399741	5064	Bacteria
114	Candidatus Blochmannia floridanus	bfl	Candidatus Blochmannia floridanus	203907	631	Bacteria
115	Candidatus Blochmannia pennsylvanicus	bpn	Candidatus Blochmannia pennsylvanicus str. BPEN	291272	658	Bacteria
116	Haemophilus influenzae (serotype d)	hin	Haemophilus influenzae Rd KW20	71421	1789	Bacteria

117	Haemophilus influenzae 86-028NP (nontypeable)	hit	Haemophilus influenzae 86-028NP	281310	1899	Bacteria
118	Haemophilus influenzae PittEE	hip	Haemophilus influenzae PittEE	374930	1704	Bacteria
119	Haemophilus influenzae PittGG	hiq	Haemophilus influenzae PittGG	374931	1753	Bacteria
120	Haemophilus ducreyi	hdu	Haemophilus ducreyi 35000HP	233412	1838	Bacteria
121	Haemophilus somnus	hso	Haemophilus somnus 129PT	205914	1879	Bacteria
122	Haemophilus somnus 2336	hsm	Haemophilus somnus 2336	228400	2065	Bacteria
123	Pasteurella multocida	pmu	Pasteurella multocida subsp. multocida str. Pm70	272843	2092	Bacteria
124	Mannheimia succiniciproducens	msu	Mannheimia succiniciproducens MBEL55E	221988	2459	Bacteria
125	Actinobacillus pleuropneumoniae	apl	Actinobacillus pleuropneumoniae L20	416269	2109	Bacteria
126	Actinobacillus pleuropneumoniae serovar 3 JL03	apj	Actinobacillus pleuropneumoniae serovar 3 str. JL03	434271	2147	Bacteria
127	Actinobacillus succinogenes	asu	Actinobacillus succinogenes 130Z	339671	2199	Bacteria
128	Xylella fastidiosa 9a5c	xfa	Xylella fastidiosa 9a5c	160492	2905	Bacteria
129	Xylella fastidiosa Temecula1	xft	Xylella fastidiosa Temecula1	183190	2125	Bacteria
130	Xylella fastidiosa M12	xfm	Xylella fastidiosa M12	405440	2368	Bacteria
131	Xylella fastidiosa M23	xfn	Xylella fastidiosa M23	405441	2320	Bacteria
132	Xanthomonas campestris pv. campestris ATCC 33913	xcc	Xanthomonas campestris pv. campestris str. ATCC 33913	190485	4242	Bacteria
133	Xanthomonas campestris pv. campestris 8004	xcb	Xanthomonas campestris pv. campestris str. 8004	314565	4370	Bacteria
134	Xanthomonas campestris pv. vesicatoria	xcv	Xanthomonas campestris pv. vesicatoria str. 85-10	316273	4854	Bacteria
135	Xanthomonas axonopodis	xac	Xanthomonas axonopodis pv. citri str. 306	190486	4489	Bacteria
136	Xanthomonas oryzae KACC10331	xoo	Xanthomonas oryzae pv. oryzae KACC10331	291331	4362	Bacteria
137	Xanthomonas oryzae MAFF311018	xom	Xanthomonas oryzae pv. oryzae MAFF 311018	342109	4431	Bacteria
138	Vibrio cholerae O1	vch	Vibrio cholerae O1 biovar El Tor str. N16961	243277	4008	Bacteria
139	Vibrio cholerae O395	vco	Vibrio cholerae O395	345073	3998	Bacteria
140	Vibrio vulnificus CMCP6	vvu	Vibrio vulnificus CMCP6	216895	4623	Bacteria
141	Vibrio vulnificus YJ016	vvy	Vibrio vulnificus YJ016	196600	5227	Bacteria
142	Vibrio parahaemolyticus	vpa	Vibrio parahaemolyticus RIMD 2210633	223926	4992	Bacteria
143	Vibrio fischeri	vfi	Vibrio fischeri ES114	312309	3957	Bacteria
144	Vibrio harveyi	vha	Vibrio harveyi ATCC BAA-1116	338187	6253	Bacteria
145	Photobacterium profundum	ppr	Photobacterium profundum SS9	298386	5702	Bacteria
146	Pseudomonas aeruginosa PAO1	pae	Pseudomonas aeruginosa PAO1	208964	5651	Bacteria
147	Pseudomonas aeruginosa UCBPP-PA14	pau	Pseudomonas aeruginosa UCBPP-PA14	208963	5977	Bacteria
148	Pseudomonas aeruginosa PA7	pap	Pseudomonas aeruginosa PA7	381754	6369	Bacteria
149	Pseudomonas putida KT2440	ppu	Pseudomonas putida KT2440	160488	5516	Bacteria
150	Pseudomonas putida F1	ppf	Pseudomonas putida F1	351746	5405	Bacteria
151	Pseudomonas putida GB-1	ppg	Pseudomonas putida GB-1	76869	5530	Bacteria
152	Pseudomonas putida W619	ppw	Pseudomonas putida W619	390235	5309	Bacteria
153	Pseudomonas syringae pv. tomato DC3000	pst	Pseudomonas syringae pv. tomato str. DC3000	223283	5810	Bacteria
154	Pseudomonas syringae pv. syringae B728a	psb	Pseudomonas syringae pv. syringae B728a	205918	5220	Bacteria
155	Pseudomonas syringae pv. phaseolicola 1448A	psp	Pseudomonas syringae pv. phaseolicola 1448A	264730	5436	Bacteria
156	Pseudomonas fluorescens Pf-5	pfl	Pseudomonas fluorescens Pf-5	220664	6233	Bacteria
157	Pseudomonas fluorescens PfO-1	pfo	Pseudomonas fluorescens Pf0-1	205922	5833	Bacteria
158	Pseudomonas entomophila	pen	Pseudomonas entomophila L48	384676	5275	Bacteria
159	Pseudomonas mendocina	pmy	Pseudomonas mendocina ymp	399739	4704	Bacteria
160	Pseudomonas stutzeri	psa	Pseudomonas stutzeri A1501	379731	4210	Bacteria
161	Psychrobacter arcticum	par	Psychrobacter arcticus 273-4	259536	2211	Bacteria
162	Psychrobacter cryohalolentis	pcr	Psychrobacter cryohalolentis K5	335284	2581	Bacteria
163	Psychrobacter sp. PRwf-1	prw	Psychrobacter sp. PRwf-1	349106	2477	Bacteria
164	Acinetobacter sp. ADP1	aci	Acinetobacter sp. ADP1	62977	3425	Bacteria
165	Acinetobacter baumannii	acb	Acinetobacter baumannii ATCC 17978	400667	3470	Bacteria
166	Acinetobacter baumannii SDF	abm	Acinetobacter baumannii SDF	509170	3664	Bacteria
167	Acinetobacter baumannii AYE	aby	Acinetobacter baumannii AYE	509173	3890	Bacteria
168	Shewanella oneidensis	son	Shewanella oneidensis MR-1	211586	4745	Bacteria
169	Shewanella denitrificans	sdn	Shewanella denitrificans OS217	318161	3905	Bacteria
170	Shewanella frigidimarina	sfr	Shewanella frigidimarina NCIMB 400	318167	4199	Bacteria
171	Shewanella amazonensis	saz	Shewanella amazonensis SB2B	326297	3785	Bacteria
172	Shewanella baltica OS155	sbl	Shewanella baltica OS155	325240	4721	Bacteria
173	Shewanella baltica OS185	sbm	Shewanella baltica OS185	402882	4596	Bacteria
174	Shewanella baltica OS195	sbn	Shewanella baltica OS195	399599	4859	Bacteria
175	Shewanella loihica	slo	Shewanella loihica PV-4	323850	3993	Bacteria
176	Shewanella putrefaciens	spc	Shewanella putrefaciens CN-32	319224	4134	Bacteria
177	Shewanella sediminis	sse	Shewanella sediminis HAW-EB3	425104	4666	Bacteria
178	Shewanella pealeana	spl	Shewanella pealeana ATCC 700345	398579	4438	Bacteria
179	Shewanella sp. MR-4	she	Shewanella sp. MR-4	60480	4084	Bacteria

180	Shewanella sp. MR-7	shm	Shewanella sp. MR-7	60481	4186	Bacteria
181	Shewanella sp. ANA-3	shn	Shewanella sp. ANA-3	94122	4519	Bacteria
182	Shewanella sp. W3-18-1	shw	Shewanella sp. W3-18-1	351745	4217	Bacteria
183	Shewanella halifaxensis	shl	Shewanella halifaxensis HAW-EB4	458817	4464	Bacteria
184	Shewanella woodyi ATCC51908	swd	Shewanella woodyi ATCC 51908	392500	5096	Bacteria
185	Idiomarina loihiensis	ilo	Idiomarina loihiensis L2TR	283942	2707	Bacteria
186	Colwellia psychrerythraea	cps	Colwellia psychrerythraea 34H	167879	5054	Bacteria
187	Pseudoalteromonas haloplanktis	pha	Pseudoalteromonas haloplanktis TAC125	326442	3621	Bacteria
188	Pseudoalteromonas atlantica	pat	Pseudoalteromonas atlantica T6c	342610	4397	Bacteria
189	Saccharophagus degradans	sde	Saccharophagus degradans 2-40	203122	4067	Bacteria
190	Psychromonas ingrahamii	pin	Psychromonas ingrahamii 37	357804	3863	Bacteria
191	Marinobacter aquaeolei	maq	Marinobacter aquaeolei VT8	351348	4390	Bacteria
192	Coxiella burnetii RSA 493	cbu	Coxiella burnetii RSA 493	227377	2183	Bacteria
193	Coxiella burnetii RSA 331	cbs	Coxiella burnetii RSA 331	360115	2278	Bacteria
194	Coxiella burnetii Dugway 5J108-111	cbd	Coxiella burnetii Dugway 5J108-111	434922	2265	Bacteria
195	Legionella pneumophila Philadelphia 1	lpn	Legionella pneumophila subsp. pneumophila str. Philadelphia 1	272624	3002	Bacteria
196	Legionella pneumophila Lens	lpf	Legionella pneumophila str. Lens	297245	3058	Bacteria
197	Legionella pneumophila Paris	lpp	Legionella pneumophila str. Paris	297246	3278	Bacteria
198	Legionella pneumophila Corby	lpc	Legionella pneumophila str. Corby	400673	3259	Bacteria
199	Methylococcus capsulatus	mca	Methylococcus capsulatus str. Bath	243233	3052	Bacteria
200	Francisella tularensis subsp. tularensis SCHU S4	ftu	Francisella tularensis subsp. tularensis SCHU S4	177416	1852	Bacteria
201	Francisella tularensis subsp. tularensis FSC 198	ftf	Francisella tularensis subsp. tularensis FSC198	393115	1852	Bacteria
202	Francisella tularensis subsp. tularensis WY96-3418	ftw	Francisella tularensis subsp. tularensis WY96-3418	418136	1872	Bacteria
203	Francisella tularensis subsp. holarctica LVS	ftl	Francisella tularensis subsp. holarctica	119857	2020	Bacteria
204	Francisella tularensis subsp. holarctica OSU18	fth	Francisella tularensis subsp. holarctica OSU18	393011	1932	Bacteria
205	Francisella tularensis subsp. holarctica FTNF002-00	fta	Francisella tularensis subsp. holarctica FTNF002-00	458234	1887	Bacteria
206	Francisella tularensis subsp. novicida U112	ftn	Francisella novicida U112	401614	1781	Bacteria
207	Francisella tularensis subsp. mediasiatica FSC147	ftm	Francisella tularensis subsp. mediasiatica FSC147	441952	1750	Bacteria
208	Francisella philomiragia	fph	Francisella philomiragia subsp. philomiragia ATCC 25017	484022	1970	Bacteria
209	Thiomicrospira crunogena	tcx	Thiomicrospira crunogena XCL-2	317025	2259	Bacteria
210	Nitrosococcus oceani	noc	Nitrosococcus oceani ATCC 19707	323261	3186	Bacteria
211	Alkalilimnicola ehrlichei	aeh	Alkalilimnicola ehrlichii MLHE-1	187272	2940	Bacteria
212	Halorhodospira halophila	hha	Halorhodospira halophila SL1	349124	2493	Bacteria
213	Hahella chejuensis	hch	Hahella chejuensis KCTC 2396	349521	6863	Bacteria
214	Chromohalobacter salexigens	csa	Chromohalobacter salexigens DSM 3043	290398	3409	Bacteria
215	Alcanivorax borkumensis	abo	Alcanivorax borkumensis SK2	393595	2806	Bacteria
216	Marinomonas sp. MWYL1	mmw	Marinomonas sp. MWYL1	400668	4598	Bacteria
217	Aeromonas hydrophila	aha	Aeromonas hydrophila subsp. hydrophila ATCC 7966	380703	4287	Bacteria
218	Aeromonas salmonicida	asa	Aeromonas salmonicida subsp. salmonicida A449	382245	4758	Bacteria
219	Dichelobacter nodosus	dno	Dichelobacter nodosus VCS1703A	246195	1351	Bacteria
220	Baumannia cicadellinicola	bci	Baumannia cicadellinicola str. Hc (Homalodisca coagulata)	374463	651	Bacteria
221	Candidatus Carsonella ruddii	crp	Candidatus Carsonella ruddii PV	387662	213	Bacteria
222	Candidatus Ruthia magnifica	rma	Candidatus Ruthia magnifica str. Cm (Calyptogena magnifica)	413404	1117	Bacteria
223	Candidatus Vesicomyosocius okutanii	vok	Candidatus Vesicomyosocius okutanii HA	412965	978	Bacteria
224	Neisseria meningitidis MC58 (serogroup B)	nme	Neisseria meningitidis MC58	122586	2225	Bacteria
225	Neisseria meningitidis Z2491 (serogroup A)	nma	Neisseria meningitidis Z2491	122587	2208	Bacteria
226	Neisseria meningitidis FAM18	nmc	Neisseria meningitidis FAM18	272831	2046	Bacteria
227	Neisseria meningitidis 053442	nmn	Neisseria meningitidis 053442	374833	2122	Bacteria
228	Neisseria gonorrhoeae	ngo	Neisseria gonorrhoeae FA 1090	242231	2069	Bacteria
229	Chromobacterium violaceum	cvi	Chromobacterium violaceum ATCC 12472	243365	4530	Bacteria
230	Ralstonia solanacearum	rso	Ralstonia solanacearum GMI1000	267608	5193	Bacteria
231	Ralstonia eutropha JMP134	reu	Ralstonia eutropha JMP134	264198	6616	Bacteria
232	Ralstonia eutropha H16	reh	Ralstonia eutropha H16	381666	6279	Bacteria
233	Ralstonia metallidurans	rme	Ralstonia metallidurans CH34	266264	6426	Bacteria
234	Cupriavidus taiwanensis	cti	Cupriavidus taiwanensis	164546	1623	Bacteria
235	Burkholderia mallei ATCC 23344	bma	Burkholderia mallei ATCC 23344	243160	5508	Bacteria
236	Burkholderia mallei SAVP1	bmv	Burkholderia mallei SAVP1	320388	5295	Bacteria

237	Burkholderia mallei NCTC 10229	bml	Burkholderia mallei NCTC 10229	412022	5624	Bacteria
238	Burkholderia mallei NCTC 10247	bmn	Burkholderia mallei NCTC 10247	320389	5966	Bacteria
239	Burkholderia xenovorans	bxe	Burkholderia xenovorans LB400	266265	9044	Bacteria
240	Burkholderia vietnamiensis	bvi	Burkholderia vietnamiensis G4	269482	7863	Bacteria
241	Burkholderia sp. 383	bur	Burkholderia sp. 383	269483	7826	Bacteria
242	Burkholderia cenocepacia AU1054	bcn	Burkholderia cenocepacia AU 1054	331271	6632	Bacteria
243	Burkholderia cenocepacia HI2424	bch	Burkholderia cenocepacia HI2424	331272	7032	Bacteria
244	Burkholderia cenocepacia MC0-3	bcm	Burkholderia cenocepacia MC0-3	406425	7177	Bacteria
245	Burkholderia cepacia	bam	Burkholderia ambifaria AMMD	339670	6724	Bacteria
246	Burkholderia ambifaria MC40-6	bac	Burkholderia ambifaria MC40-6	398577	6886	Bacteria
247	Burkholderia multivorans	bmu	Burkholderia multivorans ATCC 17616	395019	6373	Bacteria
248	Burkholderia pseudomallei K96243	bps	Burkholderia pseudomallei K96243	272560	5935	Bacteria
249	Burkholderia pseudomallei 1710b	bpm	Burkholderia pseudomallei 1710b	320372	6420	Bacteria
250	Burkholderia pseudomallei 1106a	bpl	Burkholderia pseudomallei 1106a	357348	7263	Bacteria
251	Burkholderia pseudomallei 668	bpd	Burkholderia pseudomallei 668	320373	7307	Bacteria
252	Burkholderia thailandensis	bte	Burkholderia thailandensis E264	271848	5714	Bacteria
253	Burkholderia phymatum	bph	Burkholderia phymatum STM815	391038	7899	Bacteria
254	Polynucleobacter sp. QLW-P1DMWA-1	pnu	Polynucleobacter necessarius subsp. asymbioticus QLW-P1DMWA-1	312153	2132	Bacteria
255	Polynucleobacter necessarius	pne	Polynucleobacter necessarius subsp. necessarius STIR1	452638	1783	Bacteria
256	Bordetella pertussis	bpe	Bordetella pertussis Tohama I	257313	3867	Bacteria
257	Bordetella parapertussis	bpa	Bordetella parapertussis 12822	257311	4467	Bacteria
258	Bordetella bronchiseptica	bbr	Bordetella bronchiseptica RB50	257310	5072	Bacteria
259	Bordetella petrii	bpt	Bordetella petrii DSM 12804	340100	5091	Bacteria
260	Bordetella avium	bav	Bordetella avium 197N	360910	3510	Bacteria
261	Rhodoferax ferrireducens	rfr	Rhodoferax ferrireducens T118	338969	4555	Bacteria
262	Polaromonas sp. JS666	pol	Polaromonas sp. JS666	296591	5626	Bacteria
263	Polaromonas naphthalenivorans	pna	Polaromonas naphthalenivorans CJ2	365044	5064	Bacteria
264	Acidovorax avenae	aav	Acidovorax citrulli AAC00-1	397945	4858	Bacteria
265	Acidovorax sp. JS42	ajs	Acidovorax sp. JS42	232721	4383	Bacteria
266	Verminephrobacter eiseniae	vei	Verminephrobacter eiseniae EF01-2	391735	5109	Bacteria
267	Delftia acidovorans	dac	Delftia acidovorans SPH-1	398578	6153	Bacteria
268	Methylibium petroleiphilum	mpt	Methylibium petroleiphilum PM1	420662	4546	Bacteria
269	Herminiimonas arsenicoxydans	har	Herminiimonas arsenicoxydans	204773	3391	Bacteria
270	Minibacterium massiliensis	mms	Janthinobacterium sp. Marseille	375286	3750	Bacteria
271	Leptothrix cholodnii	lch	Leptothrix cholodnii SP-6	395495	4445	Bacteria
272	Nitrosomonas europaea	neu	Nitrosomonas europaea ATCC 19718	228410	2620	Bacteria
273	Nitrosomonas eutropha	net	Nitrosomonas eutropha C91	335283	2687	Bacteria
274	Nitrosospira multiformis	nmu	Nitrosospira multiformis ATCC 25196	323848	2876	Bacteria
275	Azoarcus sp. EbN1	eba	Aromatoleum aromaticum EbN1	76114	4673	Bacteria
276	Azoarcus sp. BH72	azo	Azoarcus sp. BH72	62928	4058	Bacteria
277	Dechloromonas aromatica	dar	Dechloromonas aromatica RCB	159087	4283	Bacteria
278	Thiobacillus denitrificans	tbd	Thiobacillus denitrificans ATCC 25259	292415	2879	Bacteria
279	Methylobacillus flagellatus	mfa	Methylobacillus flagellatus KT	265072	2822	Bacteria
280	Helicobacter pylori 26695	hpy	Helicobacter pylori 26695	85962	1630	Bacteria
281	Helicobacter pylori J99	hpj	Helicobacter pylori J99	85963	1535	Bacteria
282	Helicobacter pylori HPAG1	hpa	Helicobacter pylori HPAG1	357544	1586	Bacteria
283	Helicobacter hepaticus	hhe	Helicobacter hepaticus ATCC 51449	235279	1915	Bacteria
284	Helicobacter acinonychis	hac	Helicobacter acinonychis str. Sheeba	382638	1660	Bacteria
285	Wolinella succinogenes	wsu	Wolinella succinogenes DSM 1740	273121	2091	Bacteria
286	Thiomicrospira denitrificans	tdn	Sulfurimonas denitrificans DSM 1251	326298	2163	Bacteria
287	Campylobacter jejuni NCTC11168	cje	Campylobacter jejuni subsp. jejuni NCTC 11168	192222	1708	Bacteria
288	Campylobacter jejuni RM1221	cjr	Campylobacter jejuni RM1221	195099	1940	Bacteria
289	Campylobacter jejuni 81-176	cjj	Campylobacter jejuni subsp. jejuni 81-176	354242	1812	Bacteria
290	Campylobacter jejuni 81116	cju	Campylobacter jejuni subsp. jejuni 81116	407148	1681	Bacteria
291	Campylobacter jejuni subsp. doylei 269.97	cjd	Campylobacter jejuni subsp. doylei 269.97	360109	2040	Bacteria
292	Campylobacter fetus	cff	Campylobacter fetus subsp. fetus 82-40	360106	1820	Bacteria
293	Campylobacter curvus	ccv	Campylobacter curvus 525.92	360105	2114	Bacteria
294	Campylobacter hominis ATCC BAA-381	cha	Campylobacter hominis ATCC BAA-381	360107	1804	Bacteria
295	Campylobacter concisus 13826	cco	Campylobacter concisus 13826	360104	2195	Bacteria
296	Arcobacter butzleri	abu	Arcobacter butzleri RM4018	367737	2333	Bacteria
297	Nitratiruptor sp. SB155-2	nis	Nitratiruptor sp. SB155-2	387092	1911	Bacteria
298	Sulfurovum sp. NBC37-1	sun	Sulfurovum sp. NBC37-1	387093	2519	Bacteria
299	Geobacter sulfurreducens	gsu	Geobacter sulfurreducens PCA	243231	3528	Bacteria
300	Geobacter metallireducens	gme	Geobacter metallireducens GS-15	269799	3635	Bacteria
301	Geobacter uraniumreducens	gur	Geobacter uraniireducens Rf4	351605	4489	Bacteria
302	Pelobacter carbinolicus	pca	Pelobacter carbinolicus DSM 2380	338963	3450	Bacteria
303	Pelobacter propionicus	ppd	Pelobacter propionicus DSM 2379	338966	3913	Bacteria
304	Desulfovibrio vulgaris Hildenborough	dvu	Desulfovibrio vulgaris str. Hildenborough	882	3634	Bacteria

305	Desulfovibrio vulgaris DP4	dvl	Desulfovibrio vulgaris DP4	391774	3190	Bacteria
306	Desulfovibrio desulfuricans	dde	Desulfovibrio desulfuricans subsp. desulfuricans str. G20	207559	3865	Bacteria
307	Lawsonia intracellularis	lip	Lawsonia intracellularis PHE/MN1-00	363253	1388	Bacteria
308	Bdellovibrio bacteriovorus	bba	Bdellovibrio bacteriovorus HD100	264462	3629	Bacteria
309	Desulfotalea psychrophila	dps	Desulfotalea psychrophila LSv54	177439	3322	Bacteria
310	Candidatus Desulfococcus oleovorans	dol	Desulfococcus oleovorans Hxd3	96561	3323	Bacteria
311	Anaeromyxobacter dehalogenans	ade	Anaeromyxobacter dehalogenans 2CP-C	290397	4419	Bacteria
312	Anaeromyxobacter sp. Fw109-5	afw	Anaeromyxobacter sp. Fw109-5	404589	4562	Bacteria
313	Myxococcus xanthus	mxa	Myxococcus xanthus DK 1622	246197	7456	Bacteria
314	Sorangium cellulosum	scl	Sorangium cellulosum 'So ce 56'	448385	9703	Bacteria
315	Syntrophus aciditrophicus	sat	Syntrophus aciditrophicus SB	56780	3219	Bacteria
316	Syntrophobacter fumaroxidans	sfu	Syntrophobacter fumaroxidans MPOB	335543	4162	Bacteria
317	Rickettsia prowazekii	rpr	Rickettsia prowazekii str. Madrid E	272947	886	Bacteria
318	Rickettsia typhi	rty	Rickettsia typhi str. Wilmington	257363	919	Bacteria
319	Rickettsia conorii	rco	Rickettsia conorii str. Malish 7	272944	1414	Bacteria
320	Rickettsia felis	rfe	Rickettsia felis URRWXCal2	293614	1551	Bacteria
321	Rickettsia bellii	rbe	Rickettsia bellii RML369-C	336407	1469	Bacteria
322	Rickettsia akari Hartford	rak	Rickettsia akari str. Hartford	293614	1294	Bacteria
323	Rickettsia bellii OSU 85-389	rbo	Rickettsia bellii OSU 85-389	391896	1512	Bacteria
324	Rickettsia canadensis McKiel	rcm	Rickettsia canadensis str. McKiel	293613	1129	Bacteria
325	Rickettsia rickettsii	rri	Rickettsia rickettsii str. 'Sheila Smith'	392021	1381	Bacteria
326	Rickettsia rickettsii Iowa	rrj	Rickettsia rickettsii str. Iowa	452659	1495	Bacteria
327	Orientia tsutsugamushi	ots	Orientia tsutsugamushi str. Boryong	357244	2216	Bacteria
328	Wolbachia wMel	wol	Wolbachia endosymbiont of Drosophila melanogaster	163164	1309	Bacteria
329	Wolbachia wBm	wbm	Wolbachia endosymbiont strain TRS of Brugia malayi	292805	940	Bacteria
330	Anaplasma marginale	ama	Anaplasma marginale str. St. Maries	234826	1005	Bacteria
331	Anaplasma phagocytophilum	aph	Anaplasma phagocytophilum HZ	212042	1411	Bacteria
332	Ehrlichia ruminantium Welgevonden (South Africa)	eru	Ehrlichia ruminantium str. Welgevonden	254945	961	Bacteria
333	Ehrlichia ruminantium Welgevonden (France)	erw	Ehrlichia ruminantium str. Welgevonden	254945	997	Bacteria
334	Ehrlichia ruminantium Gardel	erg	Ehrlichia ruminantium str. Gardel	302409	989	Bacteria
335	Ehrlichia canis	ecn	Ehrlichia canis str. Jake	269484	984	Bacteria
336	Ehrlichia chaffeensis	ech	Ehrlichia chaffeensis str. Arkansas	205920	1158	Bacteria
337	Neorickettsia sennetsu	nse	Neorickettsia sennetsu str. Miyayama	222891	973	Bacteria
338	Candidatus Pelagibacter ubique	pub	Candidatus Pelagibacter ubique HTCC1062	335992	1389	Bacteria
339	Mesorhizobium loti	mlo	Mesorhizobium loti MAFF303099	266835	7333	Bacteria
340	Mesorhizobium sp. BNC1	mes	Chelativorans sp. BNC1	266779	4684	Bacteria
341	Parvibaculum lavamentivorans	pla	Parvibaculum lavamentivorans DS-1	402881	3707	Bacteria
342	Sinorhizobium medicae	smd	Sinorhizobium medicae WSM419	366394	6587	Bacteria
343	Agrobacterium tumefaciens C58 (UWash/Dupont)	atu	Agrobacterium tumefaciens str. C58	176299	5459	Bacteria
344	Agrobacterium tumefaciens C58 (Cereon)	atc	Agrobacterium tumefaciens str. C58	176299	5360	Bacteria
345	Rhizobium etli	ret	Rhizobium etli CFN 42	347834	6093	Bacteria
346	Rhizobium leguminosarum	rle	Rhizobium leguminosarum bv. viciae 3841	216596	7348	Bacteria
347	Brucella melitensis	bme	Brucella melitensis bv. 1 str. 16M	224914	3264	Bacteria
348	Brucella melitensis biovar Abortus	bmf	Brucella melitensis biovar Abortus 2308	359391	3418	Bacteria
349	Brucella suis	bms	Brucella suis 1330	204722	3451	Bacteria
350	Brucella abortus 9-941	bmb	Brucella abortus bv. 1 str. 9-941	262698	3356	Bacteria
351	Brucella ovis	bov	Brucella ovis ATCC 25840	444178	3198	Bacteria
352	Ochrobactrum anthropi	oan	Ochrobactrum anthropi ATCC 49188	439375	4932	Bacteria
353	Bradyrhizobium japonicum	bja	Bradyrhizobium japonicum USDA 110	224911	8373	Bacteria
354	Bradyrhizobium sp. ORS278	bra	Bradyrhizobium sp. ORS278	114615	6818	Bacteria
355	Bradyrhizobium sp. BTAi1	bbt	Bradyrhizobium sp. BTAi1	288000	7810	Bacteria
356	Rhodopseudomonas palustris CGA009	rpa	Rhodopseudomonas palustris CGA009	258594	4898	Bacteria
357	Rhodopseudomonas palustris HaA2	rpb	Rhodopseudomonas palustris HaA2	316058	4772	Bacteria
358	Rhodopseudomonas palustris BisB18	rpc	Rhodopseudomonas palustris BisB18	316056	5016	Bacteria
359	Rhodopseudomonas palustris BisB5	rpd	Rhodopseudomonas palustris BisB5	316057	4492	Bacteria
360	Rhodopseudomonas palustris BisA53	rpe	Rhodopseudomonas palustris BisA53	316055	4972	Bacteria
361	Nitrobacter winogradskyi	nwi	Nitrobacter winogradskyi Nb-255	323098	3198	Bacteria
362	Nitrobacter hamburgensis	nha	Nitrobacter hamburgensis X14	323097	4741	Bacteria
363	Bartonella henselae	bhe	Bartonella henselae str. Houston-1	283166	1665	Bacteria
364	Bartonella quintana	bqu	Bartonella quintana str. Toulouse	283165	1356	Bacteria
365	Bartonella bacilliformis	bbk	Bartonella bacilliformis KC583	360095	1375	Bacteria
366	Bartonella tribocorum	btr	Bartonella tribocorum CIP 105476	382640	2171	Bacteria
367	Xanthobacter autotrophicus	xau	Xanthobacter autotrophicus Py2	78245	5155	Bacteria
368	Methylobacterium extorquens	mex	Methylobacterium extorquens PA1	419610	4956	Bacteria
369	Methylobacterium radiotolerans	mrd	Methylobacterium radiotolerans JCM 2831	426355	6626	Bacteria

370	Methylobacterium sp. 4-46	met	Methylobacterium sp. 4-46	426117	7145	Bacteria
371	Beijerinckia indica	bid	Beijerinckia indica subsp. indica ATCC 9039	395963	3982	Bacteria
372	Caulobacter crescentus	ccr	Caulobacter crescentus CB15	190650	3819	Bacteria
373	Caulobacter sp. K31	cak	Caulobacter sp. K31	366602	5515	Bacteria
374	Silicibacter pomeroyi	sil	Ruegeria pomeroyi DSS-3	246200	4348	Bacteria
375	Silicibacter sp. TM1040	sit	Ruegeria sp. TM1040	292414	3956	Bacteria
376	Rhodobacter sphaeroides 2.4.1	rsp	Rhodobacter sphaeroides 2.4.1	272943	4370	Bacteria
377	Rhodobacter sphaeroides ATCC 17029	rsh	Rhodobacter sphaeroides ATCC 17029	349101	4253	Bacteria
378	Rhodobacter sphaeroides ATCC 17025	rsq	Rhodobacter sphaeroides ATCC 17025	349102	4465	Bacteria
379	Jannaschia sp. CCS1	jan	Jannaschia sp. CCS1	290400	4336	Bacteria
380	Roseobacter denitrificans	rde	Roseobacter denitrificans OCh 114	375451	4191	Bacteria
381	Paracoccus denitrificans	pde	Paracoccus denitrificans PD1222	318586	5177	Bacteria
382	Dinoroseobacter shibae	dsh	Dinoroseobacter shibae DFL 12	398580	4271	Bacteria
383	Maricaulis maris	mmr	Maricaulis maris MCS10	394221	3133	Bacteria
384	Hyphomonas neptunium	hne	Hyphomonas neptunium ATCC 15444	228405	3568	Bacteria
385	Zymomonas mobilis	zmo	Zymomonas mobilis subsp. mobilis ZM4	264203	2058	Bacteria
386	Novosphingobium aromaticivorans	nar	Novosphingobium aromaticivorans DSM 12444	279238	4031	Bacteria
387	Sphingopyxis alaskensis	sal	Sphingopyxis alaskensis RB2256	317655	3260	Bacteria
388	Sphingomonas wittichii	swi	Sphingomonas wittichii RW1	392499	5455	Bacteria
389	Erythrobacter litoralis	eli	Erythrobacter litoralis HTCC2594	314225	3056	Bacteria
390	Gluconobacter oxydans	gox	Gluconobacter oxydans 621H	290633	2731	Bacteria
391	Granulobacter bethesdensis	gbe	Granulibacter bethesdensis CGDNIH1	391165	2498	Bacteria
392	Acidiphilium cryptum JF-5	acr	Acidiphilium cryptum JF-5	349163	3692	Bacteria
393	Rhodospirillum rubrum	rru	Rhodospirillum rubrum ATCC 11170	269796	3920	Bacteria
394	Magnetospirillum magneticum	mag	Magnetospirillum magneticum AMB-1	342108	4611	Bacteria
395	Magnetococcus sp. MC-1	mgm	Magnetococcus sp. MC-1	156889	3815	Bacteria
396	Acidobacteria bacterium	aba	Candidatus Koribacter versatilis Ellin345	204669	4834	Bacteria
397	Solibacter usitatus	sus	Candidatus Solibacter usitatus Ellin6076	234267	8002	Bacteria
398	Syntrophomonas wolfei	swo	Syntrophomonas wolfei subsp. wolfei str. Goettingen	335541	2642	Bacteria
399	Caldicellulosiruptor saccharolyticus	csc	Caldicellulosiruptor saccharolyticus DSM 8903	351627	2829	Bacteria
400	Bacillus subtilis	bsu	Bacillus subtilis subsp. subtilis str. 168	224308	4225	Bacteria
401	Bacillus halodurans	bha	Bacillus halodurans C-125	272558	4171	Bacteria
402	Bacillus anthracis Ames	ban	Bacillus anthracis str. Ames	198094	5630	Bacteria
403	Bacillus anthracis Ames 0581	bar	Bacillus anthracis str. 'Ames Ancestor'	261594	5965	Bacteria
404	Bacillus anthracis A2012	baa	Bacillus anthracis str. A2012	191218	5874	Bacteria
405	Bacillus anthracis Sterne	bat	Bacillus anthracis str. Sterne	260799	5415	Bacteria
406	Bacillus cereus ATCC 14579	bce	Bacillus cereus ATCC 14579	226900	5502	Bacteria
407	Bacillus cereus ATCC 10987	bca	Bacillus cereus ATCC 10987	222523	6014	Bacteria
408	Bacillus cereus ZK	bcz	Bacillus cereus E33L	288681	5796	Bacteria
409	Bacillus cereus subsp. cytotoxis NVH 391-98	bcy	Bacillus cytotoxicus NVH 391-98	315749	4176	Bacteria
410	Bacillus thuringiensis 97-27	btk	Bacillus thuringiensis serovar konkukian str. 97-27	281309	5343	Bacteria
411	Bacillus thuringiensis Al Hakam	btl	Bacillus thuringiensis str. Al Hakam	412694	4945	Bacteria
412	Bacillus weihenstephanensis	bwe	Bacillus weihenstephanensis KBAB4	315730	5983	Bacteria
413	Bacillus licheniformis ATCC 14580	bli	Bacillus licheniformis ATCC 14580	279010	4337	Bacteria
414	Bacillus licheniformis DSM13	bld	Bacillus licheniformis ATCC 14580	279010	4289	Bacteria
415	Bacillus clausii	bcl	Bacillus clausii KSM-K16	66692	4204	Bacteria
416	Bacillus amyloliquefaciens	bay	Bacillus amyloliquefaciens FZB42	326423	3813	Bacteria
417	Bacillus pumilus	bpu	Bacillus pumilus SAFR-032	315750	3825	Bacteria
418	Oceanobacillus iheyensis	oih	Oceanobacillus iheyensis HTE831	221109	3594	Bacteria
419	Geobacillus thermodenitrificans	gtn	Geobacillus thermodenitrificans NG80-2	420246	3590	Bacteria
420	Lysinibacillus sphaericus	lsp	Lysinibacillus sphaericus C3-41	444177	4972	Bacteria
421	Exiguobacterium sibiricum	esi	Exiguobacterium sibiricum 255-15	262543	3155	Bacteria
422	Staphylococcus aureus N315	sau	Staphylococcus aureus subsp. aureus N315	158879	2700	Bacteria
423	Staphylococcus aureus Mu50	sav	Staphylococcus aureus subsp. aureus Mu50	158878	2809	Bacteria
424	Staphylococcus aureus MW2	sam	Staphylococcus aureus subsp. aureus MW2	196620	2712	Bacteria
425	Staphylococcus aureus MRSA252	sar	Staphylococcus aureus subsp. aureus MRSA252	282458	2845	Bacteria
426	Staphylococcus aureus MSSA476	sas	Staphylococcus aureus subsp. aureus MSSA476	282459	2747	Bacteria
427	Staphylococcus aureus COL	sac	Staphylococcus aureus subsp. aureus COL	93062	2727	Bacteria
428	Staphylococcus aureus RF122	sab	Staphylococcus aureus RF122	273036	2663	Bacteria
429	Staphylococcus aureus USA300	saa	Staphylococcus aureus subsp. aureus USA300_FPR3757	451515	2692	Bacteria
430	Staphylococcus aureus USA300 TCH1516	sax	Staphylococcus aureus subsp. aureus USA300_TCH1516	451516	2835	Bacteria
431	Staphylococcus aureus NCTC8325	sao	Staphylococcus aureus subsp. aureus NCTC 8325	93061	2969	Bacteria
432	Staphylococcus aureus JH9	saj	Staphylococcus aureus subsp. aureus JH9	359786	2847	Bacteria

433	Staphylococcus aureus JH1	sah	Staphylococcus aureus subsp. aureus JH1	359787	2906	Bacteria
434	Staphylococcus aureus Newman	sae	Staphylococcus aureus subsp. aureus str. Newman	426430	2687	Bacteria
435	Staphylococcus epidermidis ATCC 12228	sep	Staphylococcus epidermidis ATCC 12228	176280	2561	Bacteria
436	Staphylococcus epidermidis RP62A	ser	Staphylococcus epidermidis RP62A	176279	2668	Bacteria
437	Staphylococcus haemolyticus	sha	Staphylococcus haemolyticus JCSC1435	279808	2753	Bacteria
438	Staphylococcus saprophyticus	ssp	Staphylococcus saprophyticus subsp. saprophyticus ATCC 15305	342451	2595	Bacteria
439	Listeria monocytogenes EGD-e	lmo	Listeria monocytogenes EGD-e	169963	2940	Bacteria
440	Listeria monocytogenes F2365	lmf	Listeria monocytogenes str. 4b F2365	265669	2934	Bacteria
441	Listeria innocua	lin	Listeria innocua Clip11262	272626	3145	Bacteria
442	Listeria welshimeri SLCC5334	lwe	Listeria welshimeri serovar 6b str. SLCC5334	386043	2864	Bacteria
443	Lactococcus lactis subsp. lactis IL1403	lla	Lactococcus lactis subsp. lactis Il1403	272623	2425	Bacteria
444	Lactococcus lactis subsp. cremoris SK11	llc	Lactococcus lactis subsp. cremoris SK11	272622	2742	Bacteria
445	Lactococcus lactis subsp. cremoris MG1363	llm	Lactococcus lactis subsp. cremoris MG1363	416870	2585	Bacteria
446	Streptococcus pyogenes SF370 (serotype M1)	spy	Streptococcus pyogenes M1 GAS	160490	1811	Bacteria
447	Streptococcus pyogenes MGAS5005 (serotype M1)	spz	Streptococcus pyogenes MGAS5005	293653	1950	Bacteria
448	Streptococcus pyogenes MGAS8232 (serotype M18)	spm	Streptococcus pyogenes MGAS8232	186103	1863	Bacteria
449	Streptococcus pyogenes MGAS315 (serotype M3)	spg	Streptococcus pyogenes MGAS315	198466	1951	Bacteria
450	Streptococcus pyogenes SSI-1 (serotype M3)	sps	Streptococcus pyogenes SSI-1	193567	1933	Bacteria
451	Streptococcus pyogenes MGAS10270 (serotype M3)	sph	Streptococcus pyogenes MGAS10270	370552	2067	Bacteria
452	Streptococcus pyogenes MGAS10750 (serotype M3)	spi	Streptococcus pyogenes MGAS10750	370554	2060	Bacteria
453	Streptococcus pyogenes MGAS2096 (serotype M3)	spj	Streptococcus pyogenes MGAS2096	370553	1979	Bacteria
454	Streptococcus pyogenes MGAS9429 (serotype M3)	spk	Streptococcus pyogenes MGAS9429	370551	1962	Bacteria
455	Streptococcus pyogenes Manfredo (serotype M5)	spf	Streptococcus pyogenes str. Manfredo	160491	1907	Bacteria
456	Streptococcus pyogenes MGAS10394 (serotype M6)	spa	Streptococcus pyogenes MGAS10394	286636	1971	Bacteria
457	Streptococcus pyogenes MGAS6180 (serotype M28)	spb	Streptococcus pyogenes MGAS6180	319701	1977	Bacteria
458	Streptococcus pneumoniae TIGR4	spn	Streptococcus pneumoniae TIGR4	170187	2303	Bacteria
459	Streptococcus pneumoniae R6	spr	Streptococcus pneumoniae R6	171101	2116	Bacteria
460	Streptococcus pneumoniae D39	spd	Streptococcus pneumoniae D39	373153	2069	Bacteria
461	Streptococcus pneumoniae Hungary19A 6	spv	Streptococcus pneumoniae Hungary19A-6	487214	2402	Bacteria
462	Streptococcus agalactiae 2603 (serotype V)	sag	Streptococcus agalactiae 2603V/R	208435	2276	Bacteria
463	Streptococcus agalactiae NEM316 (serotype III)	san	Streptococcus agalactiae NEM316	211110	2235	Bacteria
464	Streptococcus agalactiae A909 (serotype Ia)	sak	Streptococcus agalactiae A909	205921	2136	Bacteria
465	Streptococcus mutans	smu	Streptococcus mutans UA159	210007	2042	Bacteria
466	Streptococcus thermophilus CNRZ1066	stc	Streptococcus thermophilus CNRZ1066	299768	2000	Bacteria
467	Streptococcus thermophilus LMG18311	stl	Streptococcus thermophilus LMG 18311	264199	1974	Bacteria
468	Streptococcus thermophilus LMD-9	ste	Streptococcus thermophilus LMD-9	322159	2009	Bacteria
469	Streptococcus sanguinis	ssa	Streptococcus sanguinis SK36Links	388919	2348	Bacteria
470	Streptococcus suis 05ZYH33	ssu	Streptococcus suis 05ZYH33	391295	2254	Bacteria
471	Streptococcus suis 98HAH33	ssv	Streptococcus suis 98HAH33	391296	2253	Bacteria
472	Streptococcus gordonii	sgo	Streptococcus gordonii str. Challis substr. CH1	467705	2150	Bacteria
473	Lactobacillus plantarum	lpl	Lactobacillus plantarum WCFS1	220668	3185	Bacteria
474	Lactobacillus johnsonii	ljo	Lactobacillus johnsonii NCC 533	257314	1918	Bacteria
475	Lactobacillus acidophilus	lac	Lactobacillus acidophilus NCFM	272621	1936	Bacteria
476	Lactobacillus sakei	lsa	Lactobacillus sakei subsp. sakei 23K	314315	1963	Bacteria
477	Lactobacillus salivarius	lsl	Lactobacillus salivarius UCC118	362948	2184	Bacteria
478	Lactobacillus delbrueckii ATCC 11842	ldb	Lactobacillus delbrueckii subsp. bulgaricus ATCC 11842	390333	2217	Bacteria
479	Lactobacillus delbrueckii ATCC BAA-365	lbu	Lactobacillus delbrueckii subsp. bulgaricus ATCC BAA-365	321956	2040	Bacteria
480	Lactobacillus brevis	lbr	Lactobacillus brevis ATCC 367	387344	2351	Bacteria

481	Lactobacillus casei	lca	Lactobacillus casei ATCC 334	321967	2929	Bacteria
482	Lactobacillus gasseri	lga	Lactobacillus gasseri ATCC 33323	324831	1898	Bacteria
483	Lactobacillus reuteri	lre	Lactobacillus reuteri DSM 20016	557436	2027	Bacteria
484	Pediococcus pentosaceus	ppe	Pediococcus pentosaceus ATCC 25745	278197	1847	Bacteria
485	Enterococcus faecalis	efa	Enterococcus faecalis V583	226185	3413	Bacteria
486	Oenococcus oeni	ooe	Oenococcus oeni PSU-1	203123	1864	Bacteria
487	Leuconostoc mesenteroides	lme	Leuconostoc mesenteroides subsp. mesenteroides ATCC 8293	203120	2108	Bacteria
488	Leuconostoc citreum	lci	Leuconostoc citreum KM20	349519	1905	Bacteria
489	Symbiobacterium thermophilum	sth	Symbiobacterium thermophilum IAM 14863	292459	3455	Bacteria
490	Clostridium acetobutylicum	cac	Clostridium acetobutylicum ATCC 824	272562	4022	Bacteria
491	Clostridium perfringens 13	cpe	Clostridium perfringens str. 13	195102	2849	Bacteria
492	Clostridium perfringens ATCC 13124	cpf	Clostridium perfringens ATCC 13124	195103	3017	Bacteria
493	Clostridium perfringens SM101	cpr	Clostridium perfringens SM101	289380	2722	Bacteria
494	Clostridium tetani E88	ctc	Clostridium tetani E88	212717	2506	Bacteria
495	Clostridium novyi	cno	Clostridium novyi NT	386415	2427	Bacteria
496	Clostridium thermocellum	cth	Clostridium thermocellum ATCC 27405	203119	3305	Bacteria
497	Clostridium difficile	cdf	Clostridium difficile 630	272563	3982	Bacteria
498	Clostridium botulinum A	cbo	Clostridium botulinum A str. ATCC 3502	413999	3795	Bacteria
499	Clostridium botulinum A ATCC 19397	cba	Clostridium botulinum A str. ATCC 19397	441770	3696	Bacteria
500	Clostridium botulinum A Hall	cbh	Clostridium botulinum A str. Hall	441771	3569	Bacteria
501	Clostridium botulinum A3 Loch Maree	cbl	Clostridium botulinum A3 str. Loch Maree	498214	4105	Bacteria
502	Clostridium botulinum B1	cbb	Clostridium botulinum B1 str. Okra	498213	3975	Bacteria
503	Clostridium botulinum F	cbf	Clostridium botulinum F str. Langeland	441772	3856	Bacteria
504	Clostridium beijerinckii	cbe	Clostridium beijerinckii NCIMB 8052	290402	5243	Bacteria
505	Clostridium kluyveri	ckl	Clostridium kluyveri DSM 555	431943	3994	Bacteria
506	Clostridium phytofermentans	cpy	Clostridium phytofermentans ISDg	357809	4023	Bacteria
507	Alkaliphilus metalliredigens	amt	Alkaliphilus metalliredigens QYMF	293826	4941	Bacteria
508	Alkaliphilus oremlandii	aoe	Alkaliphilus oremlandii OhILAs	350688	2991	Bacteria
509	Carboxydothermus hydrogenoformans	chy	Carboxydothermus hydrogenoformans Z-2901	246194	2707	Bacteria
510	Desulfitobacterium hafniense	dsy	Desulfitobacterium hafniense Y51	138119	5137	Bacteria
511	Desulfotomaculum reducens	drm	Desulfotomaculum reducens MI-1	349161	3423	Bacteria
512	Pelotomaculum thermopropionicum	pth	Pelotomaculum thermopropionicum SI	370438	2977	Bacteria
513	Candidatus Desulforudis audaxviator	dau	Candidatus Desulforudis audaxviator MP104C	477974	2295	Bacteria
514	Thermoanaerobacter tengcongensis	tte	Thermoanaerobacter tengcongensis MB4	273068	2721	Bacteria
515	Thermoanaerobacter sp. X514	tex	Thermoanaerobacter sp. X514	399726	2479	Bacteria
516	Thermoanaerobacter pseudethanolicus	tpd	Thermoanaerobacter pseudethanolicus ATCC 33223	340099	2366	Bacteria
517	Moorella thermoacetica	mta	Moorella thermoacetica ATCC 39073	264732	2615	Bacteria
518	Mycoplasma genitalium	mge	Mycoplasma genitalium G37	243273	525	Bacteria
519	Mycoplasma pneumoniae	mpn	Mycoplasma pneumoniae M129	272634	733	Bacteria
520	Mycoplasma pulmonis	mpu	Mycoplasma pulmonis UAB CTIP	272635	815	Bacteria
521	Mycoplasma penetrans	mpe	Mycoplasma penetrans HF-2	272633	1069	Bacteria
522	Mycoplasma gallisepticum	mga	Mycoplasma gallisepticum R	233150	781	Bacteria
523	Mycoplasma mycoides	mmy	Mycoplasma mycoides subsp. mycoides SC str. PG1	272632	1052	Bacteria
524	Mycoplasma mobile	mmo	Mycoplasma mobile 163K	267748	667	Bacteria
525	Mycoplasma hyopneumoniae 232	mhy	Mycoplasma hyopneumoniae 232	295358	727	Bacteria
526	Mycoplasma hyopneumoniae J	mhj	Mycoplasma hyopneumoniae J	262719	709	Bacteria
527	Mycoplasma hyopneumoniae 7448	mhp	Mycoplasma hyopneumoniae 7448	262722	711	Bacteria
528	Mycoplasma synoviae	msy	Mycoplasma synoviae 53	262723	728	Bacteria
529	Mycoplasma capricolum	mcp	Mycoplasma capricolum subsp. capricolum ATCC 27343	340047	867	Bacteria
530	Ureaplasma urealyticum	uur	Ureaplasma parvum serovar 3 str. ATCC 700970	273119	653	Bacteria
531	Ureaplasma parvum	upa	Ureaplasma parvum serovar 3 str. ATCC 27815	505682	641	Bacteria
532	Phytoplasma OY	poy	Onion yellows phytoplasma OY-M	262768	793	Bacteria
533	Phytoplasma AYWB	ayw	Aster yellows witches'-broom phytoplasma AYWB	322098	730	Bacteria
534	Acholeplasma laidlawii	acl	Acholeplasma laidlawii PG-8A	441768	1433	Bacteria
535	Mesoplasma florum	mfl	Mesoplasma florum L1	265311	717	Bacteria
536	Mycobacterium tuberculosis H37Rv	mtu	Mycobacterium tuberculosis H37Rv	83332	4048	Bacteria
537	Mycobacterium tuberculosis CDC1551	mtc	Mycobacterium tuberculosis CDC1551	83331	4293	Bacteria
538	Mycobacterium tuberculosis H37Ra	mra	Mycobacterium tuberculosis H37Ra	419947	4084	Bacteria
539	Mycobacterium tuberculosis F11	mtf	Mycobacterium tuberculosis F11	336982	3998	Bacteria
540	Mycobacterium bovis AF2122/97	mbo	Mycobacterium bovis AF2122/97	233413	4003	Bacteria
541	Mycobacterium bovis BCG Pasteur 1173P2	mbb	Mycobacterium bovis BCG str. Pasteur 1173P2	410289	4036	Bacteria
542	Mycobacterium leprae	mle	Mycobacterium leprae TN	272631	2770	Bacteria

543	Mycobacterium avium paratuberculosis	mpa	Mycobacterium avium subsp. paratuberculosis K-10	262316	4399	Bacteria
544	Mycobacterium avium 104	mav	Mycobacterium avium 104	243243	5313	Bacteria
545	Mycobacterium smegmatis	msm	Mycobacterium smegmatis str. MC2 155	246196	6938	Bacteria
546	Mycobacterium ulcerans	mul	Mycobacterium ulcerans Agy99	362242	4981	Bacteria
547	Mycobacterium vanbaalenii	mva	Mycobacterium vanbaalenii PYR-1	350058	6136	Bacteria
548	Mycobacterium gilvum	mgi	Mycobacterium gilvum PYR-GCK	350054	5669	Bacteria
549	Mycobacterium sp. MCS	mmc	Mycobacterium sp. MCS	164756	5698	Bacteria
550	Mycobacterium sp. KMS	mkm	Mycobacterium sp. KMS	189918	6079	Bacteria
551	Mycobacterium sp. JLS	mjl	Mycobacterium sp. JLS	164757	5845	Bacteria
552	Corynebacterium glutamicum ATCC 13032 (Kyowa Hakko)	cgl	Corynebacterium glutamicum ATCC 13032	196627	3073	Bacteria
553	Corynebacterium glutamicum ATCC 13032 (Bielefeld)	cgb	Corynebacterium glutamicum ATCC 13032	196627	3138	Bacteria
554	Corynebacterium glutamicum R	cgt	Corynebacterium glutamicum R	340322	3156	Bacteria
555	Corynebacterium efficiens	cef	Corynebacterium efficiens YS-314	196164	3020	Bacteria
556	Corynebacterium diphtheriae	cdi	Corynebacterium diphtheriae NCTC 13129	257309	2389	Bacteria
557	Corynebacterium jeikeium	cjk	Corynebacterium jeikeium K411	306537	2181	Bacteria
558	Corynebacterium urealyticum	cur	Corynebacterium urealyticum DSM 7109	504474	2084	Bacteria
559	Nocardia farcinica	nfa	Nocardia farcinica IFM 10152	247156	6000	Bacteria
560	Rhodococcus sp. RHA1	rha	Rhodococcus jostii RHA1	101510	9221	Bacteria
561	Streptomyces coelicolor	sco	Streptomyces coelicolor A3(2)	100226	8301	Bacteria
562	Streptomyces avermitilis	sma	Streptomyces avermitilis MA-4680	227882	7762	Bacteria
563	Tropheryma whipplei Twist	twh	Tropheryma whipplei str. Twist	203267	862	Bacteria
564	Tropheryma whipplei TW08/27	tws	Tropheryma whipplei TW08/27	218496	839	Bacteria
565	Leifsonia xyli xyli CTCB07	lxx	Leifsonia xyli subsp. xyli str. CTCB07	281090	2376	Bacteria
566	Arthrobacter sp. FB24	art	Arthrobacter sp. FB24	290399	4605	Bacteria
567	Arthrobacter aurescens	aau	Arthrobacter aurescens TC1	290340	4780	Bacteria
568	Renibacterium salmoninarum	rsa	Renibacterium salmoninarum ATCC 33209	288705	3558	Bacteria
569	Kocuria rhizophila	krh	Kocuria rhizophila DC2201	378753	2414	Bacteria
570	Propionibacterium acnes	pac	Propionibacterium acnes KPA171202	267747	2368	Bacteria
571	Nocardioides sp. JS614	nca	Nocardioides sp. JS614	196162	5026	Bacteria
572	Thermobifida fusca	tfu	Thermobifida fusca YX	269800	3184	Bacteria
573	Frankia sp. CcI3	fra	Frankia sp. CcI3	106370	4618	Bacteria
574	Frankia sp. EAN1pec	fre	Frankia sp. EAN1pec	298653	7377	Bacteria
575	Frankia alni	fal	Frankia alni ACN14a	326424	6786	Bacteria
576	Acidothermus cellulolyticus	ace	Acidothermus cellulolyticus 11B	351607	2217	Bacteria
577	Kineococcus radiotolerans	kra	Kineococcus radiotolerans SRS30216	266940	4778	Bacteria
578	Saccharopolyspora erythraea	sen	Saccharopolyspora erythraea NRRL 2338	405948	7264	Bacteria
579	Salinispora tropica	stp	Salinispora tropica CNB-440	369723	4654	Bacteria
580	Salinispora arenicola	saq	Salinispora arenicola CNS-205	391037	5172	Bacteria
581	Bifidobacterium longum	blo	Bifidobacterium longum NCC2705	206672	1800	Bacteria
582	Bifidobacterium adolescentis	bad	Bifidobacterium adolescentis ATCC 15703	367928	1701	Bacteria
583	Rubrobacter xylanophilus	rxy	Rubrobacter xylanophilus DSM 9941	266117	3281	Bacteria
584	Fusobacterium nucleatum	fnu	Fusobacterium nucleatum subsp. nucleatum ATCC 25586	190304	2129	Bacteria
585	Rhodopirellula baltica	rba	Rhodopirellula baltica SH 1	243090	7405	Bacteria
586	Opitutus terrae	ote	Opitutus terrae PB90-1	452637	4705	Bacteria
587	Chlamydia trachomatis serovar D	ctr	Chlamydia trachomatis D/UW-3/CX	272561	940	Bacteria
588	Chlamydia trachomatis serovar A	cta	Chlamydia trachomatis A/HAR-13	315277	963	Bacteria
589	Chlamydia muridarum	cmu	Chlamydia muridarum Nigg	243161	962	Bacteria
590	Chlamydophila pneumoniae CWL029	cpn	Chlamydophila pneumoniae CWL029	115713	1122	Bacteria
591	Chlamydophila pneumoniae AR39	cpa	Chlamydophila pneumoniae AR39	115711	1167	Bacteria
592	Chlamydophila pneumoniae J138	cpj	Chlamydophila pneumoniae J138	138677	1110	Bacteria
593	Chlamydophila pneumoniae TW183	cpt	Chlamydophila pneumoniae TW-183	182082	1155	Bacteria
594	Chlamydophila caviae	cca	Chlamydophila caviae GPIC	227941	1060	Bacteria
595	Chlamydophila abortus	cab	Chlamydophila abortus S26/3	218497	1003	Bacteria
596	Chlamydophila felis	cfe	Chlamydophila felis Fe/C-56	264202	1054	Bacteria
597	Candidatus Protochlamydia amoebophila	pcu	Candidatus Protochlamydia amoebophila UWE25	264201	2075	Bacteria
598	Borrelia burgdorferi	bbu	Borrelia burgdorferi B31	224326	1701	Bacteria
599	Borrelia garinii	bga	Borrelia garinii PBi	290434	970	Bacteria
600	Borrelia afzelii	baf	Borrelia afzelii PKo	390236	1253	Bacteria
601	Treponema pallidum	tpa	Treponema pallidum subsp. pallidum str. Nichols	243276	1095	Bacteria
602	Treponema denticola	tde	Treponema denticola ATCC 35405	243275	2838	Bacteria
603	Leptospira interrogans serovar lai	lil	Leptospira interrogans serovar Lai str. 56601	189518	4768	Bacteria
604	Leptospira interrogans serovar Copenhageni	lic	Leptospira interrogans serovar Copenhageni str. Fiocruz L1-130	267671	3753	Bacteria
605	Leptospira borgpetersenii JB197	lbj	Leptospira borgpetersenii serovar Hardjo-bovis JB197	355277	3242	Bacteria
606	Leptospira borgpetersenii L550	lbl	Leptospira borgpetersenii serovar Hardjo-bovis L550	355276	3273	Bacteria

607	Leptospira biflexa serovar Patoc Patoc 1 (Paris)	lbi	Leptospira biflexa serovar Patoc strain 'Patoc 1 (Paris)'	456481	3440	Bacteria
608	Synechocystis sp. PCC6803	syn	Synechocystis sp. PCC 6803	1148	3314	Bacteria
609	Synechococcus sp. WH8102	syw	Synechococcus sp. WH 8102	84588	2581	Bacteria
610	Synechococcus elongatus PCC6301	syc	Synechococcus elongatus PCC 6301	269084	2582	Bacteria
611	Synechococcus elongatus PCC7942	syf	Synechococcus elongatus PCC 7942	1140	2715	Bacteria
612	Synechococcus sp. CC9605	syd	Synechococcus sp. CC9605	110662	2756	Bacteria
613	Synechococcus sp. CC9902	sye	Synechococcus sp. CC9902	316279	2358	Bacteria
614	Synechococcus sp. CC9311	syg	Synechococcus sp. CC9311	64471	2944	Bacteria
615	Synechococcus sp. RCC307	syr	Synechococcus sp. RCC307	316278	2583	Bacteria
616	Synechococcus sp. WH7803	syx	Synechococcus sp. WH 7803	32051	2586	Bacteria
617	Synechococcus sp. PCC7002	syp	Synechococcus sp. PCC 7002	32049	3237	Bacteria
618	Cyanobacteria Yellowstone A-Prime	cya	Synechococcus sp. JA-3-3Ab	321327	2897	Bacteria
619	Cyanobacteria Yellowstone B-Prime	cyb	Synechococcus sp. JA-2-3B'a(2-13)	321332	2942	Bacteria
620	Thermosynechococcus elongatus	tel	Thermosynechococcus elongatus BP-1	197221	2525	Bacteria
621	Gloeobacter violaceus	gvi	Gloeobacter violaceus PCC 7421	251221	4482	Bacteria
622	Anabaena sp. PCC7120	ana	Nostoc sp. PCC 7120	103690	6214	Bacteria
623	Anabaena variabilis	ava	Anabaena variabilis ATCC 29413	240292	5764	Bacteria
624	Prochlorococcus marinus SS120	pma	Prochlorococcus marinus subsp. marinus str. CCMP1375	167539	1930	Bacteria
625	Prochlorococcus marinus MED4	pmm	Prochlorococcus marinus subsp. pastoris str. CCMP1986	59919	1763	Bacteria
626	Prochlorococcus marinus MIT9313	pmt	Prochlorococcus marinus str. MIT 9313	74547	2330	Bacteria
627	Prochlorococcus marinus NATL2A	pmn	Prochlorococcus marinus str. NATL2A	59920	2229	Bacteria
628	Prochlorococcus marinus MIT9312	pmi	Prochlorococcus marinus str. MIT 9312	74546	1856	Bacteria
629	Prochlorococcus marinus AS9601	pmb	Prochlorococcus marinus str. AS9601	146891	1984	Bacteria
630	Prochlorococcus marinus MIT 9515	pmc	Prochlorococcus marinus str. MIT 9515	167542	1965	Bacteria
631	Prochlorococcus marinus MIT 9303	pmf	Prochlorococcus marinus str. MIT 9303	59922	3136	Bacteria
632	Prochlorococcus marinus MIT 9301	pmg	Prochlorococcus marinus str. MIT 9301	167546	1963	Bacteria
633	Prochlorococcus marinus MIT 9215	pmh	Prochlorococcus marinus str. MIT 9215	93060	2055	Bacteria
634	Prochlorococcus marinus MIT 9211	pmj	Prochlorococcus marinus str. MIT 9211	93059	1901	Bacteria
635	Prochlorococcus marinus NATL1A	pme	Prochlorococcus marinus str. NATL1A	167555	2250	Bacteria
636	Trichodesmium erythraeum	ter	Trichodesmium erythraeum IMS101	203124	5126	Bacteria
637	Bacteroides thetaiotaomicron	bth	Bacteroides thetaiotaomicron VPI-5482	226186	4902	Bacteria
638	Bacteroides fragilis YCH46	bfr	Bacteroides fragilis YCH46	295405	4717	Bacteria
639	Bacteroides fragilis NCTC9343	bfs	Bacteroides fragilis NCTC 9343	272559	4395	Bacteria
640	Bacteroides vulgatus	bvu	Bacteroides vulgatus ATCC 8482	435590	4183	Bacteria
641	Porphyromonas gingivalis	pgi	Porphyromonas gingivalis W83	242619	2015	Bacteria
642	Parabacteroides distasonis	pdi	Parabacteroides distasonis ATCC 8503	435591	3965	Bacteria
643	Salinibacter ruber	sru	Salinibacter ruber DSM 13855	309807	2898	Bacteria
644	Cytophaga hutchinsonii	chu	Cytophaga hutchinsonii ATCC 33406	269798	3837	Bacteria
645	Gramella forsetii	gfo	Gramella forsetii KT0803	411154	3637	Bacteria
646	Flavobacterium johnsoniae	fjo	Flavobacterium johnsoniae UW101	376686	5137	Bacteria
647	Flavobacterium psychrophilum	fps	Flavobacterium psychrophilum JIP02/86	402612	2499	Bacteria
648	Chlorobaculum tepidum	cte	Chlorobium tepidum TLS	194439	2344	Bacteria
649	Chlorobium chlorochromatii	cch	Chlorobium chlorochromatii CaD3	340177	2050	Bacteria
650	Chlorobium phaeobacteroides	cph	Chlorobium phaeobacteroides DSM 266	290317	2799	Bacteria
651	Prosthecochloris vibrioformis	pvi	Chlorobium phaeovibrioides DSM 265	290318	1824	Bacteria
652	Pelodictyon luteolum	plt	Chlorobium luteolum DSM 273	319225	2137	Bacteria
653	Dehalococcoides ethenogenes	det	Dehalococcoides ethenogenes 195	243164	1642	Bacteria
654	Dehalococcoides sp. CBDB1	deh	Dehalococcoides sp. CBDB1	255470	1510	Bacteria
655	Dehalococcoides sp. BAV1	deb	Dehalococcoides sp. BAV1	216389	1436	Bacteria
656	Roseiflexus sp. RS-1	rrs	Roseiflexus sp. RS-1	357808	4682	Bacteria
657	Roseiflexus castenholzii DSM13941	rca	Roseiflexus castenholzii DSM 13941	383372	4509	Bacteria
658	Chloroflexus aurantiacus	cau	Chloroflexus aurantiacus J-10-fl	324602	3990	Bacteria
659	Herpetosiphon aurantiacus	hau	Herpetosiphon aurantiacus ATCC 23779	316274	5363	Bacteria
660	Deinococcus radiodurans	dra	Deinococcus radiodurans R1	243230	3244	Bacteria
661	Deinococcus geothermalis	dge	Deinococcus geothermalis DSM 11300	319795	3150	Bacteria
662	Thermus thermophilus HB27	tth	Thermus thermophilus HB27	262724	2263	Bacteria
663	Thermus thermophilus HB8	ttj	Thermus thermophilus HB8	300852	2291	Bacteria
664	Aquifex aeolicus	aae	Aquifex aeolicus VF5	224324	1611	Bacteria
665	Thermotoga maritima	tma	Thermotoga maritima MSB8	243274	1928	Bacteria
666	Thermotoga petrophila	tpt	Thermotoga petrophila RKU-1	390874	1864	Bacteria
667	Thermotoga lettingae	tle	Thermotoga lettingae TMO	416591	2110	Bacteria
668	Thermosipho melanesiensis	tme	Thermosipho melanesiensis BI429	391009	1992	Bacteria
669	Fervidobacterium nodosum	fno	Fervidobacterium nodosum Rt17-B1	381764	1847	Bacteria
670	Petrotoga mobilis	pmo	Petrotoga mobilis SJ95	403833	2015	Bacteria
671	Methanococcus jannaschii	mja	Methanocaldococcus jannaschii DSM 2661	243232	1830	Archaea
672	Methanococcus maripaludis S2	mmp	Methanococcus maripaludis S2	267377	1772	Archaea
673	Methanococcus maripaludis C5	mmq	Methanococcus maripaludis C5	402880	1889	Archaea
674	Methanococcus maripaludis C6	mmx	Methanococcus maripaludis C6	444158	1888	Archaea
675	Methanococcus maripaludis C7	mmz	Methanococcus maripaludis C7	426368	1855	Archaea
676	Methanococcus aeolicus	mae	Methanococcus aeolicus Nankai-3	419665	1552	Archaea

677	Methanococcus vannielii	mvn	Methanococcus vannielii SB	406327	1752	Archaea
678	Methanosarcina acetivorans	mac	Methanosarcina acetivorans C2A	188937	4721	Archaea
679	Methanosarcina barkeri	mba	Methanosarcina barkeri str. Fusaro	269797	3831	Archaea
680	Methanosarcina mazei	mma	Methanosarcina mazei Go1	192952	3436	Archaea
681	Methanococcoides burtonii	mbu	Methanococcoides burtonii DSM 6242	259564	2497	Archaea
682	Methanosaeta thermophila	mtp	Methanosaeta thermophila PT	349307	1781	Archaea
683	Methanospirillum hungatei	mhu	Methanospirillum hungatei JF-1	323259	3304	Archaea
684	Methanocorpusculum labreanum	mla	Methanocorpusculum labreanum Z	410358	1819	Archaea
685	Methanoculleus marisnigri	mem	Methanoculleus marisnigri JR1	368407	2555	Archaea
686	Candidatus Methanoregula boonei	mbn	Candidatus Methanoregula boonei 6A8	456442	2513	Archaea
687	Methanobacterium thermoautotrophicum	mth	Methanothermobacter thermautotrophicus str. Delta H	187420	1921	Archaea
688	Methanosphaera stadtmanae	mst	Methanosphaera stadtmanae DSM 3091	339860	1588	Archaea
689	Methanobrevibacter smithii ATCC 35061	msi	Methanobrevibacter smithii ATCC 35061	420247	1835	Archaea
690	Methanopyrus kandleri	mka	Methanopyrus kandleri AV19	190192	1729	Archaea
691	Archaeoglobus fulgidus	afu	Archaeoglobus fulgidus DSM 4304	224325	2486	Archaea
692	Halobacterium salinarum R1	hsl	Halobacterium salinarum R1	478009	2934	Archaea
693	Haloarcula marismortui	hma	Haloarcula marismortui ATCC 43049	272569	4301	Archaea
694	Thermoplasma acidophilum	tac	Thermoplasma acidophilum DSM 1728	273075	1530	Archaea
695	Thermoplasma volcanium	tvo	Thermoplasma volcanium GSS1	273116	1548	Archaea
696	Picrophilus torridus	pto	Picrophilus torridus DSM 9790	263820	1582	Archaea
697	Pyrococcus horikoshii	pho	Pyrococcus horikoshii OT3	70601	2005	Archaea
698	Pyrococcus abyssi	pab	Pyrococcus abyssi GE5	272844	1995	Archaea
699	Pyrococcus furiosus	pfu	Pyrococcus furiosus DSM 3638	186497	2228	Archaea
700	Thermococcus kodakaraensis	tko	Thermococcus kodakarensis KOD1	69014	2358	Archaea
701	Uncultured methanogenic archaeon RC-I	rci	uncultured methanogenic archaeon RC-I	351160	3166	Archaea
702	Aeropyrum pernix	ape	Aeropyrum pernix K1	272557	1752	Archaea
703	Staphylothermus marinus	smr	Staphylothermus marinus F1	399550	1646	Archaea
704	Ignicoccus hospitalis	iho	Ignicoccus hospitalis KIN4/I	453591	1492	Archaea
705	Hyperthermus butylicus	hbu	Hyperthermus butylicus DSM 5456	415426	1672	Archaea
706	Sulfolobus solfataricus	sso	Sulfolobus solfataricus P2	273057	3033	Archaea
707	Sulfolobus tokodaii	sto	Sulfolobus tokodaii str. 7	273063	2874	Archaea
708	Sulfolobus acidocaldarius	sai	Sulfolobus acidocaldarius DSM 639	330779	2329	Archaea
709	Metallosphaera sedula	mse	Metallosphaera sedula DSM 5348	399549	2342	Archaea
710	Pyrobaculum aerophilum	pai	Pyrobaculum aerophilum str. IM2	178306	2706	Archaea
711	Pyrobaculum islandicum	pis	Pyrobaculum islandicum DSM 4184	384616	2062	Archaea
712	Pyrobaculum arsenaticum	pas	Pyrobaculum arsenaticum DSM 13514	340102	2408	Archaea
713	Caldivirga maquilingensis	cma	Caldivirga maquilingensis IC-167	397948	2045	Archaea
714	Thermoproteus neutrophilus	tne	Thermoproteus neutrophilus V24Sta	444157	2053	Archaea
715	Thermofilum pendens	tpe	Thermofilum pendens Hrk 5	368408	1931	Archaea
716	Nitrosopumilus maritimus	nmr	Nitrosopumilus maritimus SCM1	436308	1840	Archaea
717	Nanoarchaeum equitans	neq	Nanoarchaeum equitans Kin4-M	228908	582	Archaea
718	Candidatus Korarchaeum cryptofilum	kcr	Candidatus Korarchaeum cryptofilum OPF8	374847	1661	Archaea
